# Feralisation targets different genomic loci to domestication in the chicken

**DOI:** 10.1038/ncomms12950

**Published:** 2016-09-30

**Authors:** M. Johnsson, E. Gering, P. Willis, S. Lopez, L. Van Dorp, G. Hellenthal, R. Henriksen, U. Friberg, D. Wright

**Affiliations:** 1 AVIAN Behavioural Genomics and Physiology Group, IFM Biology, Department of Zoology, Linköping University, 58183 Linköping, Sweden; 2 Department of Zoology, Michigan University, Michigan 48824, USA; 3 Department of Biology, University of Victoria, Victoria, British Columbia, Canada V8P 5C2; 4 UCL Genetics Institute, Department of Genetics, Evolution and Environment, University College London, London WC1E 6BT, UK; 5 Centre for Mathematics, Physics and Engineering in the Life Sciences and EXperimental Biology (CoMPLEX), University College London, London WC1E 6BT, UK

## Abstract

Feralisation occurs when a domestic population recolonizes the wild, escaping its previous restricted environment, and has been considered as the reverse of domestication. We have previously shown that Kauai Island's feral chickens are a highly variable and admixed population. Here we map selective sweeps in feral Kauai chickens using whole-genome sequencing. The detected sweeps were mostly unique to feralisation and distinct to those selected for during domestication. To ascribe potential phenotypic functions to these genes we utilize a laboratory-controlled equivalent to the Kauai population—an advanced intercross between Red Junglefowl and domestic layer birds that has been used previously for both QTL and expression QTL studies. Certain sweep genes exhibit significant correlations with comb mass, maternal brooding behaviour and fecundity. Our analyses indicate that adaptations to feral and domestic environments involve different genomic regions and feral chickens show some evidence of adaptation at genes associated with sexual selection and reproduction.

## Abstract (web summary)

Feralisation or the reintroduction of domestic animals into the wild population requires adaptation to survive. Here, the authors analyse the genetics of domestic chickens released into the wild and identify genomic regions that are selected for during feralisation.

## Graphical abstract

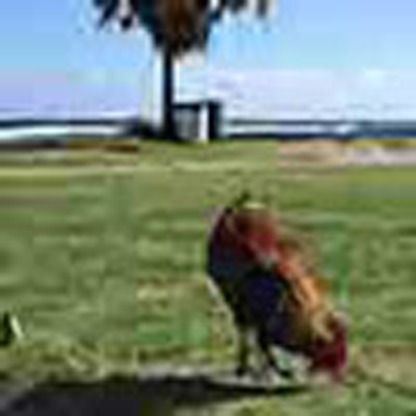

Feralisation occurs when a domestic population is returned to the wild and has been considered as the reverse of domestication^1^. Domestication itself has been an area of intense study ever since Darwin^2^, and has been used as a model for evolution and the effects of strong directional selection. It has been used to identify genes affecting a number of traits that change with selection^3^,^4^, while in contrast, almost nothing is known about the genomic changes associated with feralisation. The process of feralisation involves a massive increase in both natural and sexual selection, with predation, foraging requirements and mate competition once more exerting strong effects on a once domesticated, now feral, population. Feralisation thus offers a unique opportunity to observe how natural and sexual selection acts on a domestic population returned to natural conditions, and especially how the genome responds to the reintroduction of such strong selective forces. The genomic changes induced by feralisation can therefore identify genes affecting sexual selection and life history traits in the wild. While domestication has taught us about evolution under artificial conditions, feralisation thus provides an equal opportunity to study evolution under natural conditions. Studies of feralisation, in addition, also promise to answer how lasting the effects of domestication are, as well as the degree to which evolution is reversible.

On the Hawaiian island of Kauai, the domestic chicken was accidentally released to the wild by the tropical storms Iniki and Ewa in the 1980s and 1990, where these birds bred with a small reservoir of birds of Polynesian origin, likely Red Junglefowl that have been present on the island since ∼1,200AD (ref. 5). Ever since, these chickens have roamed freely on the island, where they yet again are fully exposed to predation, mate choice, parasitic load and the host of other pressures associated with natural environments. The hybridization between wild and domestic birds has therefore led to a large increase in genomic diversity, giving a large degree of variation on which selection can act. Hence, the Kauai chickens are a case study of feralisation and admixture, and the ensuing genomic and phenotypic alterations. This population will have the return of natural selection and sexual selection pressures once again, while having access to Red Junglefowl alleles that may have been lost in domestication, and therefore offers a unique opportunity to study the effects such as feralisation selection has on the genome, and to contrast it with the effects of domestication. In the case of the wild Red Junglefowl (the progenitor of the modern domestic chicken), a wide variety of both natural and sexual selection has been shown to act on this species, with effects on multiple diverse traits ranging from behaviour^6^,^7^ and morphology^8^,^9^ to comb mass and plumage^10^,^11^,^12^,^13^. Plumage is sexually dimorphic in this species, ranging from highly conspicuous reds and metallic greens in male birds, to cryptic browns in females. Comb mass is under strong sexual selection, with the chicken considered a classical model for such selection pressures^14^, and has been linked genetically with both fecundity^9^,^15^ and bone allocation^16^,^17^. In contrast, domestic chickens display a variety of colour variants (Dominant White, Dun, Smoky, barring and so on), show much increased fecundity and growth, and also modify a variety of behaviours, from reduced brooding (to help maximize egg production) to decreased fear responses^18^,^19^,^20^.

We have previously shown that the Kauai chicken population is admixed between domestic chickens and wild Red Junglefowl, using evidence from mitochondrial genotypes, vocalizations and plumage measurements from birds sampled at eight different locations^21^. Birds were found to possess two distinct Mt haplogroups—the domestic ‘E' haplogroup (*n*=20), and the Pacific ‘D' haplogroup (*n*=3). The ‘E' haplogroup is found in domestic chickens of recent European origin, whereas the ‘D' haplogroup is exclusively found in Pacific birds. The Mt control regions (CRs) of these ‘D' haplogroup birds were found to be highly similar to ancient Hawaiian birds that predate European contact^21^. Similarly, vocalizations (considered a classic distinguishing feature of wild Red Junglefowl birds—the last syllable is shortened) were found to be highly variable (encompassing both domestic and Red Junglefowl values), with third and fourth syllables significantly different to both wild and domestic birds^21^. Finally, plumage traits were found to be variable, with birds ranging from the classic Red Junglefowl plumage (red and green feathers), to those with white flecks and some other more unique patterning in a small number of individuals (for example, mostly white or black). These results all indicate that the feral chickens present in Kauai are primarily of domestic origin, but with admixture from Red Junglefowl population(s).

Selective sweep mapping can be used to identify genomic regions that have been fixed by selection, and that are therefore of potential relevance to such selective regimes. Notably, this technique has been used to identify putative signatures of selection caused by domestication in chickens^22^, dogs^23^ and pigs^24^, amongst others. Here we map selective sweeps in the feral Kauai chickens by means of massively parallel sequencing of 23 feral birds (114 × coverage in total for all birds) to identify putative regions of selection under feralisation and compare them with sweep regions previously found in domestic chickens (see Supplementary Table 1 for the sequencing depth and location bird samples were obtained from). Regions that show extremely low heterozygosity, have high Tajima's D or *F*_ST_ relative to domestic or Red Junglefowl populations, are considered putative selective sweep regions^25^. Here we identify potential selective sweeps with multiple population genetic approaches, and use the index of fixation (*F*_ST_) and chromosome painting to compare the putatively selected regions with domestic chickens. An important caveat with this analysis is that without data from before the selective forces, and ideally at numerous points during the selection, it is almost impossible to guarantee that the observed ‘sweep' regions do truly represent a selective sweep. In the case of the study presented here therefore this must always been born in mind when putative sweeps are identified, as such additional data are yet to be available.

## Results

### Heterozygosity sweep mapping

**Figure 1:**
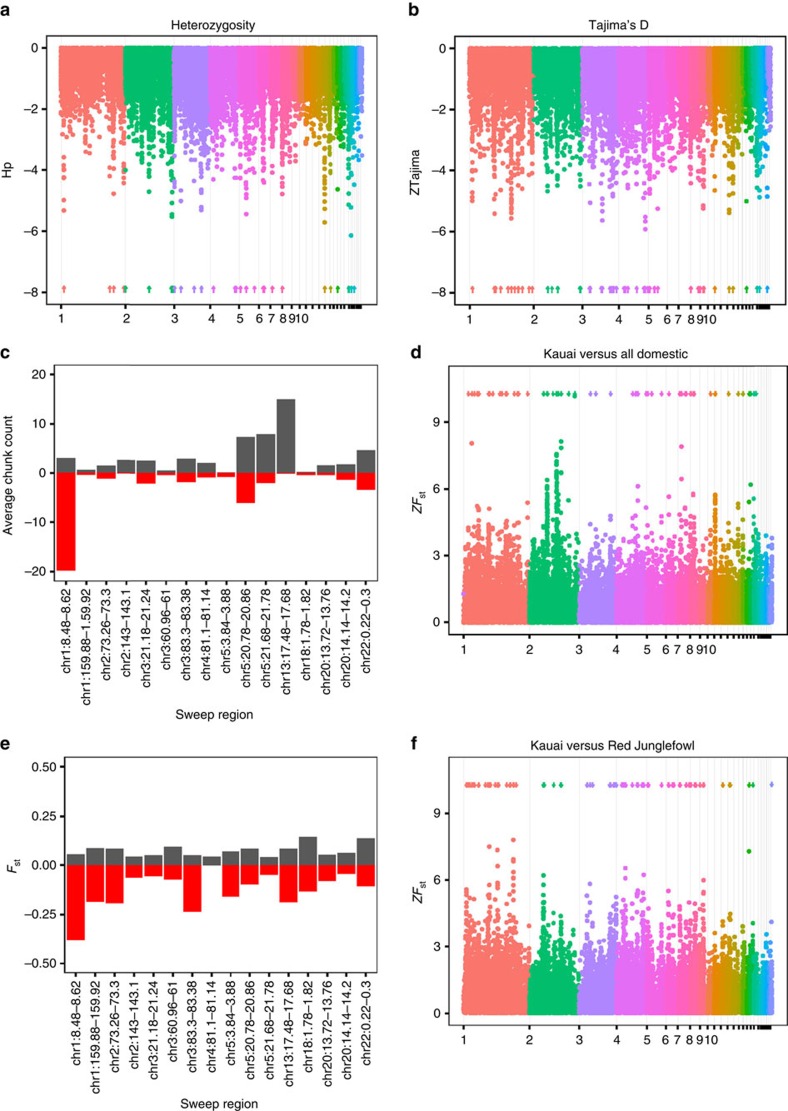
Sweep mapping in the Kauai feral chicken. Manhattan plots of (**a**) standardized pooled heterozygosity and (**b**) Tajima's D in the Kauai population. Chromosomes have alternating colours. For clarity, the labels on microchromosomes have been suppressed. (**c**) Average number of inferred haplotype segments shared with each pooled domestic chicken (black; averaged across domestics) and pooled Red Jungefowl (red) in each sweep region inferred by Chromopainter. (**e**) Average *F*_ST_ in sweep regions, comparing Kauai with the all domestic and Red Junglefowl pool. Black bars represent Kauai versus All domestic, and Red negative bars Kauai versus Red Junglefowl. Thus the greater the bar, the greater the differentiation between the Kauai population and the Red Junglefowl population (red, negative bars) or the domestic population (black, positive bars). Manhattan plots of standardized *F*_ST_ in **d** Kauai versus All domestic and (**f**) Kauai versus Red Junglefowl.

Selective sweeps were called using three separate techniques based on heterozygosity deficiency, Tajima's D and *F*_ST_. For the heterozygosity mapping, we calculated expected heterozygosity in sliding windows of 40 kb throughout the genome of the Kauai population sample, using the same method as applied by ref. 22 to domestic chickens. As a control, we applied the same analysis to the layer, broiler and combined domestic pools used in ref. 22 to detect selective sweeps in domestic chickens. Overall, the Kauai sample had higher pooled heterozygosity (mean 0.36 and s.d. 0.036, based on 23 Kauai genomes) than the domestic pools (mean 0.29 and s.d. 0.031 in the all domestics pool, based on eight domestic pool samples; see Supplementary Fig. 1). This is consistent with previous analyses of genetic and phenotypic variation in Kauai chickens, all of which suggest an admixed population of both wild and domestic ancestry^21^. We standardized the heterozygosity to *Z*-scores, and looked at windows in the lower tail of the distribution. One window on chromosome 22 had a standardized heterozygosity (ZH_p_) less than −6 in the Kauai sample, while at the −4 threshold, 76 windows formed 37 putative sweep regions (Table 1 and Supplementary Table 2). In comparison, the All domestic pool had 38 windows at the −6 threshold and 235 windows reaching a standardized heterozygosity of −4, which formed 91 putative sweeps. Figure 1a shows a Manhattan plot of standardized heterozygosity in the Kauai population, while Supplementary Table 2 shows the ZH_p_ scores of these sweep regions in the Kauai population, with the corresponding ZH_p_ scores for these regions in the layer, broiler and all domestic samples used in the study by Rubin *et al*.^22^. We therefore find fewer and weaker signals of selection using this method in the Kauai feral population than in the domestic chicken. Most sweep regions are made up of a single 40 kb window. The median sweep length in Kauai data set is also 40 kb, and the longest, on chromosome 13, is 200 kb. In the domestic pool, the median sweep length is 60 kb. The longest, on chromosome 2, is 440 kb.

### Tajima's D sweep mapping

We also mapped sweeps using Tajima's D and the population genetic software analysis of next-generation sequencing data (ANGSD), which uses genotype likelihoods instead of SNP calls^26^ to avoid bias from genotype calling in low sequencing depth. Similar to the heterozygosity mapping, we estimated Tajima's D in 40 kb windows across the genome (Fig. 1b), and standardized the resulting distribution to *Z*-scores. We detected 128 windows of standardized Tajima's D (ZTajima) less than −4 in the Kauai sample, forming 62 putative sweep regions. Out of the initial 37 regions found by low heterozygosity, 17 also overlapped a region of low Tajima's D, suggesting a relatively good accordance between the two methods (Table 1).

### Overlap between sweep mapping techniques

With any of the above two mapping approaches, the potential sweep regions in the Kauai population are mostly distinct from the ones detected in domestic chickens. Of the 37 putative Kauai sweeps regions detected by heterozygosity mapping, eight of them overlap regions that are also detected in the All domestic pool. There were also another three overlapping regions found in the layer pool and one additional region in the broiler pool (Table 1 and Supplementary Table 2), therefore 26 unique feralisation sweeps were detected in total. When looking at regions with Tajima's D in the Kauai data, 8 of the 62 regions overlapped a domestication sweep. Out of the 17 regions detected by both heterozygosity and Tajima's D, 5 overlapped a domestication sweep.

### *F*_ST_ sweep mapping

Finally, we estimated *F*_ST_ between Kauai and the All domestic pool and a pool of eight Red Junglefowl, also from Rubin's data set (Fig. 1d,f). We standardized *F*_ST_, as above, and took the upper tail of the *ZF*_ST_ distribution. There were 204 windows with *ZF*_ST_ >4 between Kauai and the All domestic pool, forming 84 potentially differentiated regions. There were 181 windows between Kauai and the Red Junglefowl pool, forming 89 regions. There was little overlap with the putative sweeps in the Kauai population; only two sweeps overlap highly differentiated regions in the Red Junglefowl comparison. These are the chromosome 1 sweep containing *SEMA3A*, and the chromosome 4 sweep close to *ADRA2C*, both of which also overlap potential domestication sweeps. Similarly, there was once again little overlap between the sweeps detected by *F*_ST_ in the Kauai population and the domestication sweeps (those identified by ref. 22). A total of 13 out of 84 sweeps overlapped the domestication sweeps in the Kauai × domestic *F*_ST_ comparison, and 5 out of 89 sweeps overlapped the domestication sweeps in the Kauai × Red Junglefowl *F*_ST_ scan.

### GO Terms of sweep genes

The sweep regions detected by both approaches contained 30 genes, based on the Ensembl genes database. Including genes 40 kb away from the sweeps gives a set of 50 genes. The most common Gene Ontology categories were very broad terms such as protein binding, cytoplasm, nucleus and membrane. However, among common categories were also the terms related to DNA binding and extracellular exosome (Supplementary Table 3). This indicates that genes involved both in intracellular transcriptional regulation and intercellular signalling may have been selected.

### Assessment of the phenotypic function of sweep genes

**Figure 2:**
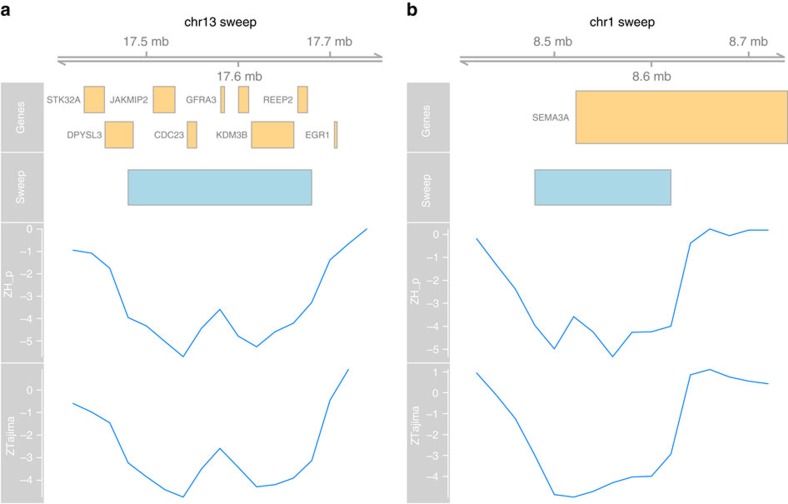
Breakdown of the chromosome 13 sweep region. (**a**) The putative sweep on chromosome 13 with standardized pooled heterozygosity and Tajima's D of the windows around the sweep, as well as gene locations based on the Ensembl gene database. (**b**) The putative sweep on chromosome 1 containing *SEMA3A*.

To ascribe potential phenotypic functions to these genes, we utilized a laboratory-controlled equivalent to the Kauai population—an eighth generation advanced intercross between Red Junglefowl and domestic layer birds that has been utilized previously for both quantitative trait loci (QTL) and expression QTL (eQTL) studies to examine comb mass and fecundity traits. Comb mass is under intense sexual selection in the Red Junglefowl^14^, but during chicken domestication, comb mass has actually increased, possibly due to correlated responses to selection on egg production (observed in the advanced intercross previously)^16^,^27^. Therefore, we assessed correlations between the candidate genes and comb mass and fecundity traits in particular in the advanced intercross. Initially, we observed that two of the genes present in the 200 kb sweep on chromosome 13, *STK32A* and *DPYSL3* (Fig. 2a) were previously identified as strong candidates for comb mass. Not only do these genes overlap a QTL for comb mass^16^, but when investigated further using gene expression in comb tissue both were found to strongly correlate with comb mass^17^. By then assessing all sweep genes for an association with comb mass, we identified an additional candidate, *ARHGAP25*, as also being significantly associated (Table 2). We then continued the search in an eQTL data set of bone tissue, and found a further 10 genes had significant associations with one or more fecundity traits (Table 2). Strong natural selection will potentially act on fecundity traits (both potentially to limit or increase, depending on lifetime fitness gains), however one trait that should certainly be under strong selection in a feral environment is the ability to brood. Without brooding behaviour eggs cannot be incubated, and will not hatch. Expression of the gene *SEM3A* (Fig. 2b) correlated with brooding behaviour (Methods), and one of four QTL identified for broodiness in the advanced intercross also overlapped with selective sweeps on chromosomes 4 (ref. 28).

We find 20 exonic SNPs located in putative sweep regions that cause amino-acid substitutions, based on the Ensembl genes database. They affect seven genes. Nine of these occur in *HERV-H LTR-associating 1* (*HHLA1*), all synonymous substitutions. One of the genes in the chromosome 13 sweep, *lysine (K)-specific demethylase 3B* (*KDM3B*), has three substitutions. Eleven of the substitutions, including six in *HHLA1* and one in *KDM3B* are already described in the Ensembl variation database.

### Sweep origin assessment

To identify the origin (that is, wild or domestic) of the selective sweep regions we used Chromopainter^29^, a haplotype-based approach, to ‘paint' these regions in the feral chickens using the Red Junglefowl and domestic haplotypes as donors. Considering the 17 common sweeps (that is, detected by both heterozygosity and Tajima's D methods), three sweeps (two on chromosome 5 and one on chromosome 13) displayed a haplotype that appears to be more closely related to one or more of the domestic breeds, while only one sweep region (on chromosome 1 at 8.48 Mb, overlapping the gene *SEMA3A*) was inferred to be more closely related to the Red Junglefowl (see Fig. 1c and Supplementary Fig. 2). We also estimated pairwise *F*_ST_ between Kauai and the all domestic and Red Junglefowl pools. Figure 1e shows the average *F*_ST_ in the putative sweeps detected by both heterozygosity and Tajima's D. For most sweeps, pairwise *F*_ST_ showed greater divergence between the Kauai versus Red Junglefowl comparison than the Kauai versus Domestic comparison. Further, several more of the sweeps appeared to be unique to the Kauai population, in that they showed similar *F*_ST_ values in each of the pairwise comparisons. Of the 37 sweeps detected by heterozygosity mapping, ∼25 of the sweep regions appear to be domestic in origin, while 6 appear derived from Red Junglefowl, using either Chromopainter or *F*_ST_ measures (Table 1). Frequently the strength of the difference (that is, the difference between the pairwise *F*_ST_ comparisons) is not extreme (Table 1 and Fig. 1e). Although the statistical significance cannot be estimated, these analyses do provide an overview of the wild or domestic relatedness of these sweep regions. Taken together the results for both *F*_ST_ and Chromopainter analyses of the sweep regions show the same overall pattern, with sweeps being closer to the domestic donor population. Despite this, there are certain occasions where the two techniques do not agree. In the case of the sweep on chromosome 1, Chromopainter placed the sweep as being derived from the Red Junglefowl donor, whereas *F*_ST_ analysis placed the sweep as being closer to the domestic population.

**Figure 3:**
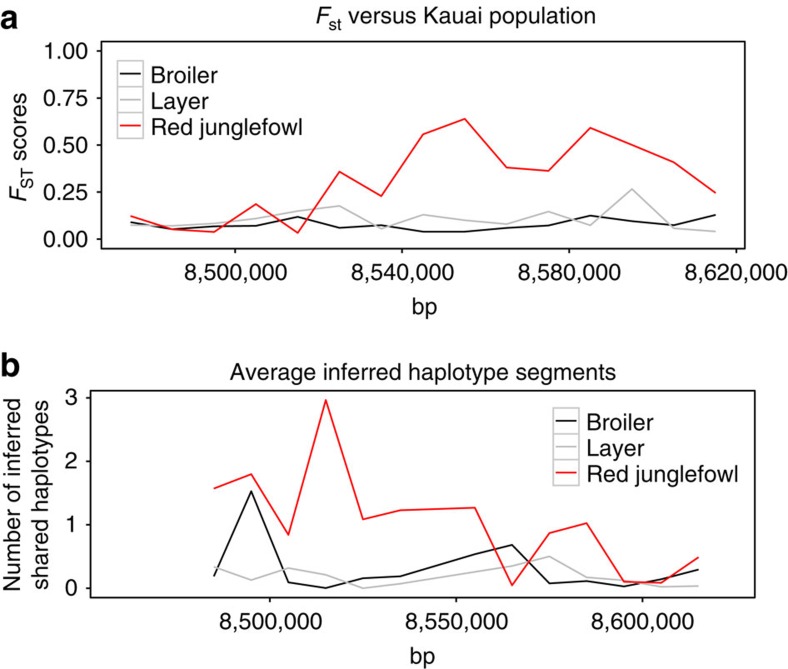
Breakdown of the sweep region on chromosome 1 sweep region at 8,500,000 bp. (**a**) *F*_ST_ estimates between Kauai, Red Junglefowl and Layer and Broiler pools, on a sliding scale along the sweep region. (**b**) Chromopainter average number of inferred haplotype segments shared in the same region, using Red Junglefowl, Layer and Broiler pools. Note that in the case of **a**, increased values indicate increased *F*_ST_ and therefore increased divergence, whereas in **b** increased number of shared segments indicates greater similarity to the population in question.

To attempt to ascertain the origin of the above region on chromosome 1, a more fine-scale Chromopainter and *F*_ST_ analysis was performed on the region using 10 kb windows. In both instances, the domestic birds were separated into layer breeds (using the two most diverse Layer pools from ref. 22) and broiler breeds (using the two most diverse broiler pools from ref. 22), see Fig. 3. This figure illustrates that Chromopainter indicates a Red Junglefowl-derived haplotype at the beginning of the sweep region (as well as spike in similarity with broiler at the start of the region), whilst the *F*_ST_ estimates are largely indistinguishable between Red Junglefowl, broiler and layer samples (that is, populations are not strongly differentiated between each other at this point). Conversely, towards the middle and end of the region *F*_ST_ estimates indicate differentiation between Kauai and Red Junglefowl samples, while Chromopainter indicates no strong similarity to any of the populations. In both *F*_ST_ and Chromopainter analyses, broiler samples appear more similar to Kauai samples on average. Thus the two methods, while not in full agreement, are not as contradictory as first appeared, with different parts of the putative sweep having potentially different origins, dependant on the method used. The putative sweep is strongest at the start of the region in question.

## Discussion

In this work, we investigate genome-wide heterozygosity and differentiation in the Kauai chicken population. We find this population to have high heterozygosity, consistent with its probable origin as an admixed population of wild and domestic chickens. We show the extent of the genomic changes that can result from the feralisation process, through the identification of sweep regions that are unique to this feral population. By combining these with a laboratory wild by domestic hybrid cross we not only identify genes in sweep regions, but tie-in actual putative functions beyond simple hypotheses based on previously ascribed gene functions, a common stumbling block with selective sweep studies. In particular, we find potential sweep regions with genes that correlate with comb mass and fecundity traits.

The majority of the sweeps detected in this feral population were unique, and displayed little overlap with sweeps previously detected for domestication^22^, regardless of the method used to detect them. However, despite the relatively low overlap with previously discovered domestication sweeps, it is still a possibility that the sweeps that have been detected are in fact due to selection that has acted on the ancestral Red Junglefowl population, before the hybridization event with the domestic birds and the resulting feralisation process. The sweeps themselves were haplotyped using both Chromopainter and *F*_ST_ measures of each area. In both cases, the major contributors to sweeps seem to be domestic chickens. This suggests that the sweeps represent regions of domestic origin that feralisation has gone on to fix in the population. Similarly, it is also adds weight to the assertion that the sweeps detected are due to feralisation selection as opposed to prior selection on the Red Junglefowl reservoir population. Furthermore, it appears that the loci under selection in the wild and in domestication are largely separate. Therefore selection on loci that are beneficial in the wild is relaxed under domestic conditions, so that they effectively evolve neutrally, and then are restored in feral conditions. Conversely, loci that are beneficial in domestic conditions are not simply selected against in feral conditions. For example, the *TSHR* and *BCDO2* mutations, known to be fixed in recent domestic populations^22^,^30^, are both freely segregating in the Kauai population. In essence, simply reversing the loci fixed during domestication does not appear to be required to adapt an individual to a feral environment in this population. Rather, separate loci (that are still segregating in domestic populations) are swept in the Kauai population. These sweeps are thus likely to be due to the renewed natural or sexual selection imposed on the population.

The question about the reversibility of domestication in feralisation is related to the wider question of the repeatability of evolution. Genomic investigations suggest that complex traits can evolve in a parallel manner by means of independent loci, and thus independent genomic signatures. For instance, two different populations of altitude adapted Tibetan chickens, display largely independent sweep signals^31^. Considering domestication, the crop plants maize and rice have little overlap between potential domestication loci as detected by selective sweep scans^32^. The evolution of weedy strains of plants is similar to feralisation in that it is an instance of natural selection on a domesticated population. Complex traits in two strains of weedy rice show little evidence of shared loci for weediness traits in a QTL mapping study^33^. Viewed in this light, our results may be another example of how quantitative traits can evolve through separate sets of loci. The genomic signature of selection then likely depends on environmental conditions, the genetic architecture before selection and chance.

With any form of selective sweep mapping there is always the issue of which of the detected signals represent an actual sweep, and which arose due to random drift^34^. This is one of the hardest problems to address with sweep mapping and in many ways can only be truly answered when the underlying mutation or polymorphism that is being selected on is identified, or at the least functional genomics can give evidence that a particular gene within the sweep region influences a relevant phenotype. In the case of the study presented here, the birds in some ways do not represent ‘pure' feralisation, as the population is a mixture of both domestic birds that are now feral, and the original wild Red Junglefowl reservoir birds that were already present on the island. As it is, this population is therefore admixed, and any modelling of the population should take this into account. Indeed, to truly model the demographics of this population original samples of the founding Red Junglefowl birds should be used, but are not currently available. Similarly, some care must be taken in interpreting the results from this feral population to other feral populations. On the other hand, this admixture could also allow more rapid response to feralisation selection due to the presence of these Red Junglefowl alleles, while it is also unclear how common such introgressions between wild and domestic individuals are in feral populations. To account for this admixture, additional forms of analysis were used both as a separate sweep mapping analysis and also to investigate the origins of the sweep regions that were detected with heterozygosity and Tajima's D sweep mapping techniques, namely, *F*_ST_ approaches and the Chromopainter software. In terms of the latter, the Chromopainter results indicate that for the common sweeps detected with both heterozygosity and Tajima's D mapping, one locus on chromosome 1 was clearly derived from the Red Junglefowl, with four other loci (on chromosomes 5, 13 and 22) being clearly derived from a domestic haplotype. Of the remainder, the majority were closer to the domestic haplotype, but these similarities were less extreme. The *F*_ST_ results from these same sweep regions broadly agree with the Chromopainter results, with loci in general being more distinct (that is, possessing a greater *F*_ST_ value) from Red Junglefowl than they are from domestic birds. However, some inconsistencies can be seen between the Chromopainter and *F*_ST_ results for these regions. Notably the sweep region on chromosome 1 that is considered of Red Junglefowl origin by Chrompainter is found to be more isolated form the Red Junglefowl by *F*_ST_ analysis. In this instance, the more detailed breakdown of the region indicated that Chromopainter assigned the beginning of the region as Red Junglefowl-derived, while *F*_ST_ estimates indicated that the middle and end of the region were closer to Broiler populations (and more distinct from Red Junglefowl). Where the two methods diverge in their assessment it is possible that where regions are small it is difficult to confidently assign the more similar donor group in the case of Chromopainter, or if the pooled Red Junglefowl samples happen to have low allelic variability in this region it could also distort *F*_ST_ estimates. The sweep itself is strongest at the start of the region, potentially suggesting that the Red Junglefowl origin is more probable (though this evidence is rather circumstantial in determining the sweep origin). To actually prove the origin of the putative sweep it would be necessary to identify the causal elements that are relevant to the phenotype(s) under selection. One possible avenue would be to identify the causal polymorphism affecting brooding behaviour in the laboratory advanced intercross and then assess this polymorphism in the Kauai population. Similarly, a GWAS study on the Kauai population itself that focussed on brooding behaviour could also narrow down the region in question.

One clear advantage of the feral population being hybrid is it enables a comparison between the laboratory hybrid advanced intercross and the Kauai population. Most notably, it allows us to test the candidate genes present in the sweep regions with the wealth of eQTL and QTL data that is possessed for this advanced intercross. This comparison of genes present in the sweep regions with the laboratory-equivalent to the feral population enables us to ascribe functions to these genes through correlations between gene expression in specific tissues with traits that are potentially affected. This is particularly powerful as it allows some functional evidence to be garnered to examine the candidate genes, and to address the largest issue that sweep studies are prone to—the actual dissection of the sweep regions themselves. Domestication in the chicken includes several phenotypic changes that are likely deleterious in a wild environment. One behavioural change that is common to modern domestic chickens, particularly layers, is the reduction in brooding behaviour. Brooding is virtually completely absent in modern layer breeds, but vital for hatching offspring in the wild. We find an overlap between a QTL identified for affecting brooding behaviour and feralisation sweeps, as well as a significant gene expression in bone tissue that correlates with various aspects of brooding and egg production/clutch size. Most notably, the gene *SEMA3A* was found to correlate not only with broodiness, but also total egg production. Chromopainter haplotype analysis indicates that, despite the feralisation sweep overlapping a previous domestication sweep, the region is more highly fixed for the Red Junglefowl or Broiler (rather than Layer) haplotype. The *F*_ST_ analysis indicated more similarity between Kauai and Domestic populations, though notably these *F*_ST_ estimates found the Broiler to be more similar to the Kauai birds on average throughout the region, as compared with the Layer pools. This could then indicate that the haplotype represents selection against the Layer haplotype. Feral chickens are not only subject to restored natural but also sexual selection. Free mate choice will once again be of major influence in this population after thousands of years of controlled breeding. The comb is a sexual ornament, and larger combs are preferred both by female and male Red Junglefowl^12^,^14^. Given this potential for strong sexual selection, it is unsurprising that two of the genes in or adjacent to the detected sweeps (*STK32A* and *DPYSL3*) had previously been identified in the laboratory intercross as affecting comb mass^16^,^17^. Further investigation of the sweep genes revealed another candidate showed an association with comb mass in the laboratory intercross, *ARHGAP25*, though only one of the nine comb mass QTL identified in the intercross overlapped feralisation sweep regions. The sweep *ARHGAP25* was located in appears to be of domestic origin, with domestic birds possessing larger combs. Similarly, several of the sweeps that are present in this feral population appear to be derived from the domestic haplotype, potentially suggesting that certain domestic haplotypes do confer higher fitness in this new environment.

Genomic responses to feral environments have important implications for conservation and food security. Rapid evolution is increasingly seen as a critical determinate of population productivity and viability^35^. Feral populations inhabit a continuum between their wild and captive relatives, and can potentially exert evolutionary influence on both^36^ via geneflow, competition and/or shared enemies and mutualists (for example, pathogens and predators). Unfortunately, we cannot yet predict the net outcomes of these exchanges. On the one hand, movement of feral genes or individuals into wild or farmed relatives could disrupt genetic adaptations to local environments, resulting in maladaptation^37^. At the same time, gene flow might enhance fitness via benefits of genetic variability^38^ and/or by introducing the sort of novel adaptations we report from Kauai's chickens. More work is needed to determine how the competing influences of feral organisms affect natural and cultivated systems, and thereby develop ‘evolutionarily enlightened' management strategies for feral populations^39^. In the case of Kauai chickens, successful colonization of both wild and urban habitats (personal observations) supports the view that feral taxa may harbour genotypes that are well suited to marginal and/or human-altered habitats, an increasingly abundant feature of the Anthropocene era^40^,^41^.

In this study, we demonstrate that feralisation in the Kauai chicken leads to specific signals of selection on the genome, rather than simply reversing those changes fixed during domestication. In particular genes affecting comb mass and fecundity appear to be targets for selection related to feralisation, though there is the potential for many more phenotypes to still be revealed as relevant. We demonstrate that by combining the complementary feral and laboratory populations it is possible to not only identify such sweeps, but also identify both phenotypes and gene functions based on this approach. The admixed nature of the population means that although the population is indeed a model of feralisation, the wild birds that were present on the island and could interbreed with the released domestics means that the population would likely possess greater genetic variation than is normal for a feral population. This can have several potential repercussions. First, it could potentially allow more rapid adaptation to the natural environment for the feral domestic birds, as key polymorphisms will already be available. Second, this may then mean the feral chickens of Kauai will not be as representative as other populations in terms of the speed of feralisation changes. To truly assess such questions, additional populations both from the Hawaiian Island chain and from other entirely distinct feral chicken populations will be required.

## Methods

### Samples and sequencing

A total of 23 chickens were utilized in the study, with these being donated by private individuals living on Kauai. DNA was extracted from blood using salt extraction techniques^42^ in Sweden (samples collected and imported under permit DRN 6.2.18-1361/13, Jordbruksverket, Sweden). These 23 samples were obtained from eight different regions of the island (see Supplementary Table 1 and Supplementary Fig. 3), with these broadly distinguished into the west, central and north of the island. Vocalization and plumage were also recorded for these birds, with these results presented in ref. 21. The same Kauai samples, their mitochondrial genomes and a subset of the nuclear variants have been used to investigate the origins of this population^5^. Approximately 5 × sequence coverage was obtained per individual (see later). Full sequence data have been deposited to the European Nucleotide Archive (BioProject Accession no. PRJNA272379, SRA Accession no. SRP052017). We also downloaded data from a previous sweep mapping effort in domestic chickens^22^ for comparison. This data consisted of eight separate pools of individuals, four of them being pools of between 8 and 11 broiler birds, three being pools of between 8–11 layer birds and one being a pool of RJF samples. Each pool was sequenced to a 5 × coverage using SOLiD sequencing technology (see ref. 22 for further information), with ∼38 × total coverage for all samples.

DNA samples were sequenced using the SOLiD 5500xl platform at Uppsala Genome Center, part of the National Genomics Infrastructure, and were analysed using computational resources provided by the Uppsala Multidisciplinary Center for Advanced Computational Science^43^. Fragment reads of 75 bp were sequenced with one individual run per lane. An average of 5 × coverage/individual was obtained, with approximately 114 × in total obtained for all the Kauai birds. Total sequence coverage for each sample is given in Supplementary Table 1, in addition to the accession numbers and mapping statistics for samples sequenced by Rubin *et al*.^22^. The reads were aligned to the chicken reference genome version Galgal4 with LifeScope Genomic Analysis Software version 2.5.1.

### Variant calling

We aligned sequence reads to the chicken reference genome (version Galgal4) with Lifescope version 2.5 (Life Technologies). Alignments were processed with Picard (http://broadinstitute.github.io/picard/) version 1.92 and GATK^44^,^45^. We removed duplicate reads (Picard MarkDuplicates), performed realignment around potential indels (GATK IndelRealigner) and recalibrated quality scores (GATK BaseRecalibrator). Finally, we called variants from all samples together with the GATK UnifiedGenotyper version 3.1.1. We filtered variants with VCFtools version 0.1.13 (ref. 46). Variants were filtered to include only those supported by at least two sequence reads, and to have at least 80% complete data. We filtered the Hawaii variants and the variants from Rubin 2010 separately.

### Calculations of pooled heterozygosity

We divided the autosomal genome into 40 kb sliding windows and calculated pooled heterozygosity including all called variants in the windows. For comparison, we also mapped and recalculated putative sweeps on the pooled samples sequenced by ref. 22. This data set comprises eight domestic breeds that were combined into a layer pool (LR), a broiler pool (CB) and the combined All domestic pool (AD). We calculated pooled heterozygosity in 40 kb sliding windows with 20 kb overlap across the autosomal genome. Windows with less than three variants were not considered, leaving ∼45,600 windows. The Kauai individuals were sequenced individually while the study by Rubin *et al*. used pools, and we therefore used slightly different formulas for the pooled heterozygosity. The pooled heterozygosity was calculated based on counts of major and minor alleles overall the variants in each window. In the Kauai samples, the number of major and minor alleles were calculated from the genotype calls. In the pooled samples, the number of reads supporting that allele was used as an approximation of major and minor allele counts. We used the R statistical environment for calculations^47^ and ggplot2 for graphics^48^.

### ANGSD analyses

We ran the empirical Bayes folded site frequency spectrum and Tajima's D estimation in ANGSD^26^,^49^. We used a mapping quality threshold of 10, a window size of 40 kb, and used the genotype likelihoods directly from GATK. As above, we standardized the Tajima's D values by subtracting the mean and dividing by the s.d., and use a threshold of −4.

### Sweep annotation

We combined consecutive windows to form candidate sweep regions. We used Ensembl genes version 79 (ref. 50); and the biomaRt package^51^ to find genes located within 40 kb of the sweep region. We used ANNOVAR^52^, again with the Ensembl genes database, to annotate variants with respect to their location and potential effects on protein-coding genes. Figure 2 was made with the Gviz package.

### Haplotype phasing and reconstruction from pooled data

Kauaian chickens' sequences were phased jointly using SHAPEIT^53^ and incorporating the sex-averaged recombination map for all chicken populations combined published in Elferink *et al*.^54^. Before phasing, data were pruned to remove triallelic SNPs, sites with a rate of missing data >0.05 and SNPs with a minor allele frequency <0.01 using PLINK v1.07 (ref. 55), leaving a total of 3,745,889 markers.

In the case of the pooled domestics and pooled Red Junglefowl from Rubin *et al*.^22^, to build the haplotypes required for Chromopainter analyses, we sampled alleles at each SNP based on the pooled data read probabilities and generated a haplotype for each strain. Where a read probability was not given we sampled with 50% probability of being each allele type.

### Chromopainter analyses

We ran Chromopainter v2 (ref. 29) to explore patterns of haplotype sharing among individuals, both at whole-genome and local (for each selective sweep) levels. Chromopainter ‘paints' each haplotype of a sampled recipient individual by identifying at each location of each recipients two haploid genomes, the best matching DNA segment from a set of sampled donor individuals. By employing a Hidden Markov model approach, Chromopainter can be used to infer the donor (or group of donors) most related genetically to any given recipient individual. We employed Chromopainter to paint each Kauaian chicken (the ferals and the Red Junglefowl ugc_610) using the reconstructed domestics and Red Junglefowl haplotypes as donors, to infer the donor haplotypes to which each Kauaian chicken shares most recent ancestry relative to the other donors. We initially estimated the switch rate (Ne) and mutation rate (Mut) by running Chromopainter on all individuals and chromosomes, using 10 steps of the Expectation–Maximization (E–M) algorithm. Then, we averaged the inferred values of each parameter across chromosomes, weighting the average by number of SNPs, and then across individuals. This gave an average Ne of 3,166 and an average Mut of 0.0287574, which were fixed in the subsequent Chromopainter analyses that analysed all individuals and all chromosomes.

To infer whether each identified regions were more closely related to the domestics or the Red Junglefowl of Rubin *et al*., these regions were each independently painted in each feral Kauai chicken using the reconstructed domestics and the Red Junglefowl as donor populations, as above. In this case, we followed the E–M approach to assign local ancestry as described in ref. 56, using 50 steps of E–M inferring the region-wide proportion of DNA matches, and rates of switches and mutation (-i 50 –in –im –ip). For the regions where the recombination rates were 0 (chr2: 73,560,000–73,600,000, chr3: 1,760,000–1,840,000, chr3: 60,960,000–61,000,000 and chr5: 3,840,000–3,880,000), Chromopainter analyses were repeated ignoring the recombination maps.

For Fig. 1c, we used average number of inferred haplotype segments shared with the domestic pools or the Red Junglefowl pool as a measure of similarity. We calculated the average number inferred haplotype segments in Kauai individuals painted with Red Junglefowl or a domestic chromosome, and plotted that in a bar chart.

### *F*_ST_ analysis

We estimated *F*_ST_ within each window with the method of moments estimator of ref. 57, making pairwise comparisons between the Kauai population and the Red Junglefowl and all domestic pools of Rubin 2010 and treating each window as a locus. For Fig. 1e, we calculated the average *F*_ST_ in comparison with domestics and Red Junglefowl in each sweep, and plotted that in a bar chart.

### QTL overlaps

The physical locations of chicken domestication QTL from refs 16, 58, 59 were moved to Galgal4. We extracted the sequences around markers and aligned them to Galgal4 with BLAT^60^. QTL region limits were defined by 1.8 LOD drops expanded to the closest marker. QTL and sweep overlaps were found with GenomicRanges^61^.

### Candidate gene expression

Gene expression had previously been measured using a variety of different tissues as an expression QTL analysis of an eighth generation intercross between White Leghorn layers and Red Junglefowl^9^,^58^. Both bone tissue collected from femoral medullary bone and comb tissue from the base of the comb were combined with genotyping of 768 SNP markers for each individual. 125 female intercross birds were used for bone tissue, with each run on a custom designed 135k probe array from Roche Nimblegen (details of the cross and experiment given in ref. 58), while 39 comb bases from male intercross birds were also run on a custom 135k probe array (details given in ref. 18). Genes identified as being within or ±40 kb from a selective sweep were then tested as potential candidates by correlating gene expression with comb mass and fecundity traits, respectively. The model used for comb mass included weight at 212 days and batch, in addition to gene expression of the candidates (each candidate was tested separately). Fecundity traits included number of eggs produced in a 2-week brooding trial, mean weight of eggs produced in this period, total weight of all eggs produced in this period, and finally a proxy of broodiness. This was calculated by first allowing the birds to lay eggs for 2 weeks, with eggs being removed daily, followed by then giving them a 2-week period, where the eggs were left with the birds. When a bird is brooding, they will reduce egg production (in Red Junglefowl capped at between six and eight eggs) and then cease production to incubate the eggs. To get a measure of this, the difference between the two trials (brooding minus the initial fecundity trial) is therefore considered a proxy to brooding, indicating when a bird has capped their egg production capacity. The model tested was fecundity trait against batch, weight and gene expression.

### Data availability

Full sequence data has been deposited to the European Nucleotide Archive (BioProject Accession no. PRJNA272379, SRA Accession no. SRP052017). Bone microarrays have been uploaded to ArrayExpress under accession number E-MTAB-3141. Advanced intercross genotype and phenotype data are present in the Supplementary Information in ref. 58. Comb microarray data (microarrays, phenotype files) is available in Dryad, doi: 10.5061/dryad.bs275. The authors declare that all other data are contained within the Article and its Supplementary files or available from the author upon request.

## Additional information

**How to cite this article:** Johnsson, M. *et al*. Feralisation targets different genomic loci to domestication in the chicken. *Nat. Commun.* **7,** 12950 doi: 10.1038/ncomms12950 (2016).

## Supplementary Material

Supplementary Information Supplementary Figures 1-3 and Supplementary Tables 1-3.

## Figures and Tables

**Table 1:**
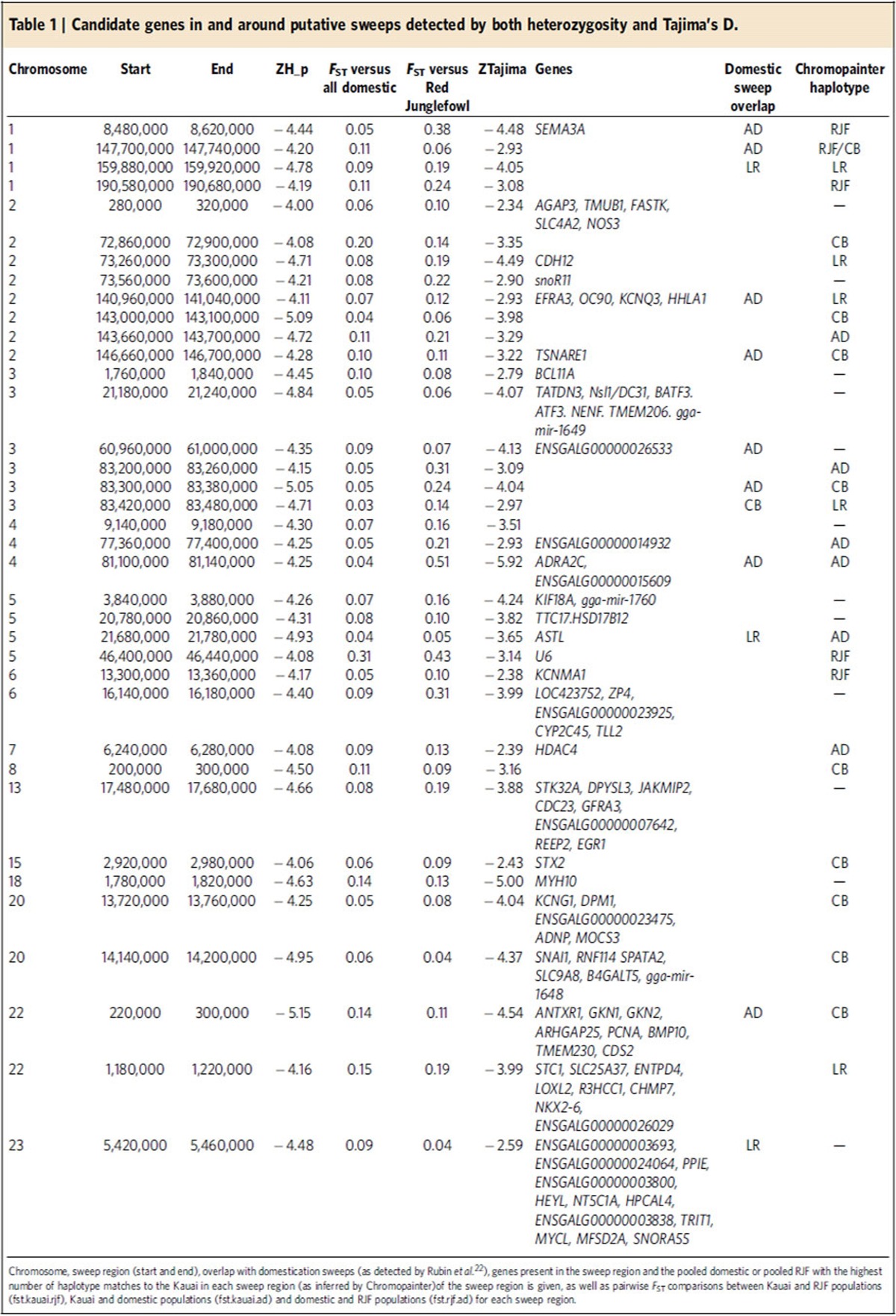
Candidate genes in and around putative sweeps detected by both heterozygosity and Tajima's D.

**Table 2:** Associations between comb and fecundity phenotypes in the wild × domestic intercross of genes in feralisation sweeps in Kauai.

**Trait**	**Genes**	***P*** **value**	**chr**	**Position**	**Probe**
Broodiness	*SEMA3A*	0.0032	1	8,522,175	NM_204977_SEMA3A
Comb mass	*ARHGAP25*	0.0040	13		NM_001030883_ARHGAP25
Comb mass	*DPYSL3*	0.0101	13	17,454,812	NM_204493_DPYSL3*
Comb mass	*STK32A*	0.0016	13	17,432,186	ENSGALT00000012246_STK32A
Egg number (brooding trial)	*EFR3A*	0.0018	2	140,874,788	ENSGALT00000026233_ENSGALG00000016270
Egg number (brooding trial)	*LOC419677*	0.0033	23	5,463,429	ENSGALT00000006094_ENSGALG00000003838
Egg number (brooding trial)	*NSL1*	0.0008	3	21,140,905	NM_001044646_NSL1
Egg number (brooding trial)	*PPIE*	0.0041	23	5,415,385	ENSGALT00000021831_ENSGALG00000013383
Egg number (brooding trial)	*SEMA3A*	0.0017	1	8,522,175	NM_204977_SEMA3A
Egg number (brooding trial)	*STX2*	0.0038	15	2,912,110	ENSGALT00000034743_ENSGALG00000002536
Mean egg weight (fecundity trial)	*NT5C1A*	0.0030	23	5,450,092	ENSGALT00000006051_ENSGALG00000003816
Mean egg weight (fecundity trial)	*PABPC4*	0.0003	23	5,420,069	ENSGALT00000006028_ENSGALG00000003800
Total egg production (brooding trial)	*EFR3A*	0.0041	2	140,874,788	ENSGALT00000026233_ENSGALG00000016270
Total egg production (brooding trial)	*BMP8A*	0.0019	23	5,408,458	ENSGALT00000040607_ENSGALG00000024064
Total egg production (brooding trial)	*LOC419677*	0.0038	23	5,463,429	ENSGALT00000006094_ENSGALG00000003838
Total egg production (brooding trial)	*MOCS3*	0.0033	20	13,729,490	ENSGALT00000012964_ENSGALG00000007986
Total egg production (brooding trial)	*NSL1*	0.0019	3	21,140,905	NM_001044646_NSL1
Total egg production (brooding trial)	*PABPC4*	0.0037	23	5,420,069	ENSGALT00000006028_ENSGALG00000003800
Total egg production (brooding trial)	*PPIE*	0.0026	23	5,415,385	ENSGALT00000021831_ENSGALG00000013383
Total egg production (brooding trial)	*SEMA3A*	0.0039	1	8,522,175	NM_204977_SEMA3A
