# Supplementary material for: Feralisation targets different genomic loci to domestication in the chicken
Source: Nat Commun. 2016 Sep 30;7:12950. doi: 10.1038/ncomms12950 (PMC5056458; doi:10.1038/ncomms12950)
Supplement: Supplementary Information — Supplementary Figures 1-3 and Supplementary Tables 1-3. [file ncomms12950-s1.pdf]

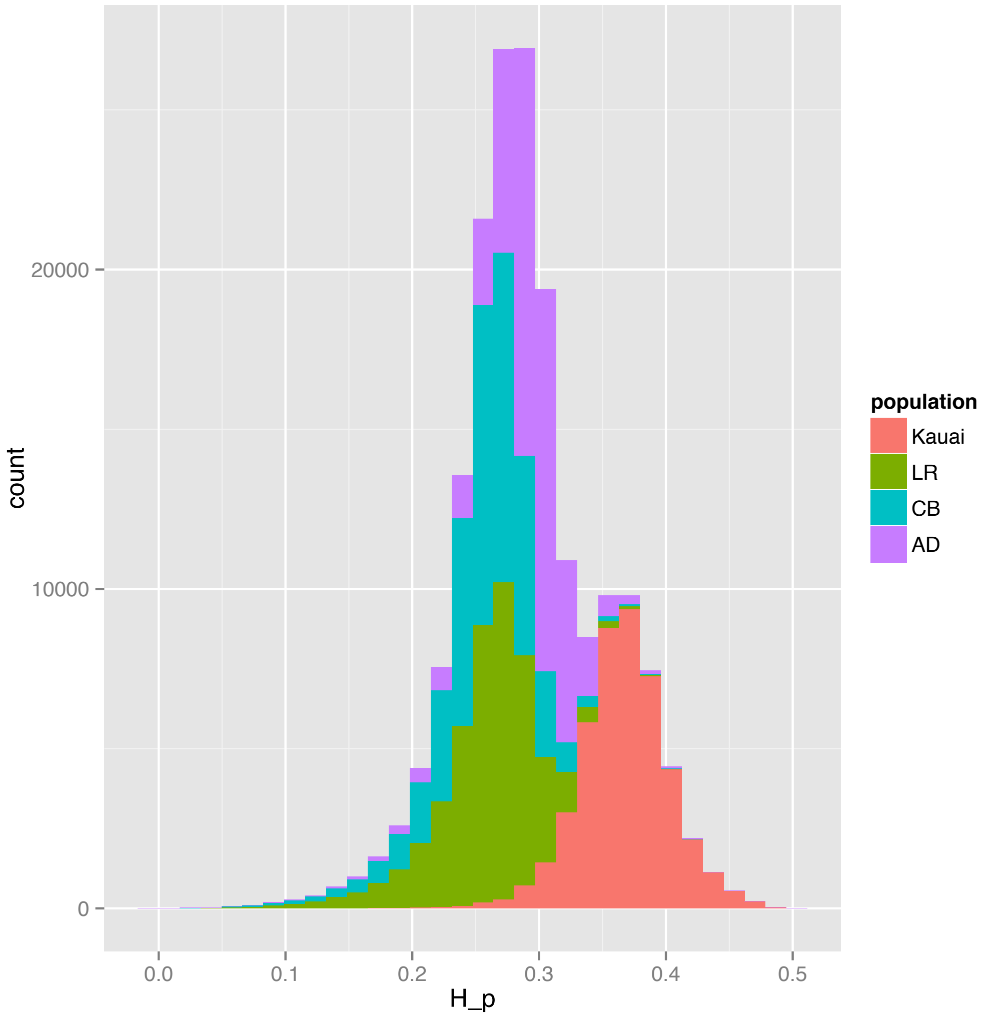

Supplementary Figure 1. Histogram of the distribution pooled heterozygosities in the Kauai sample and the all domestic (AD), layer (LR) and broiler (CB) pools.

Supplementary Figure 2. Number of segments matched to each pooled domestic chicken and pooled RJF for each haplotype of each Kauai chicken for each heterozygosity sweep region, as inferred by Chromopainter. The first page gives the average across all 37 regions.

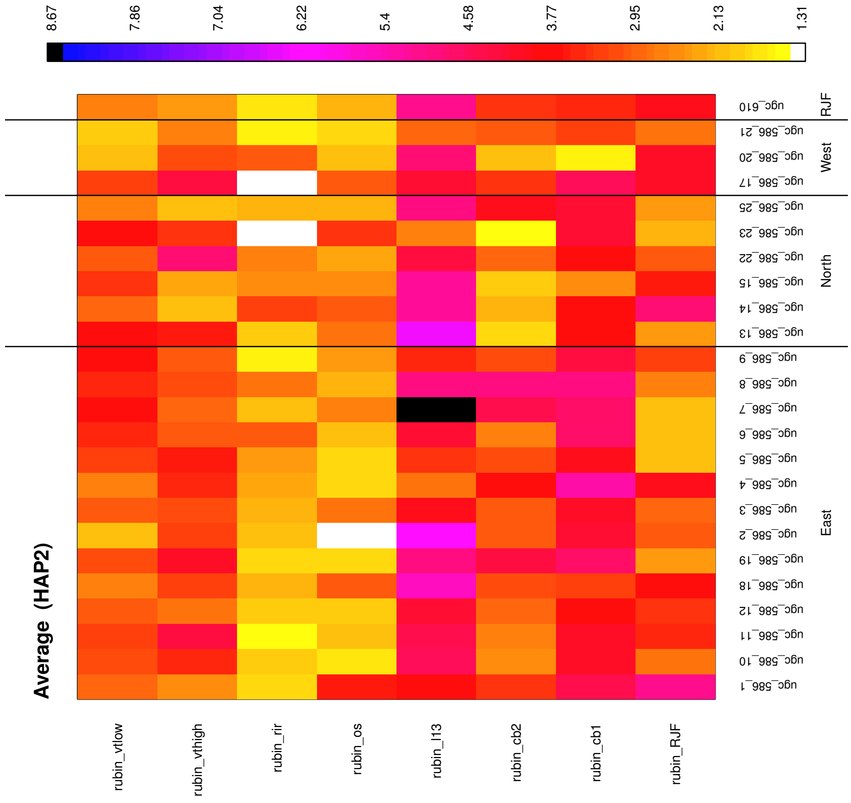

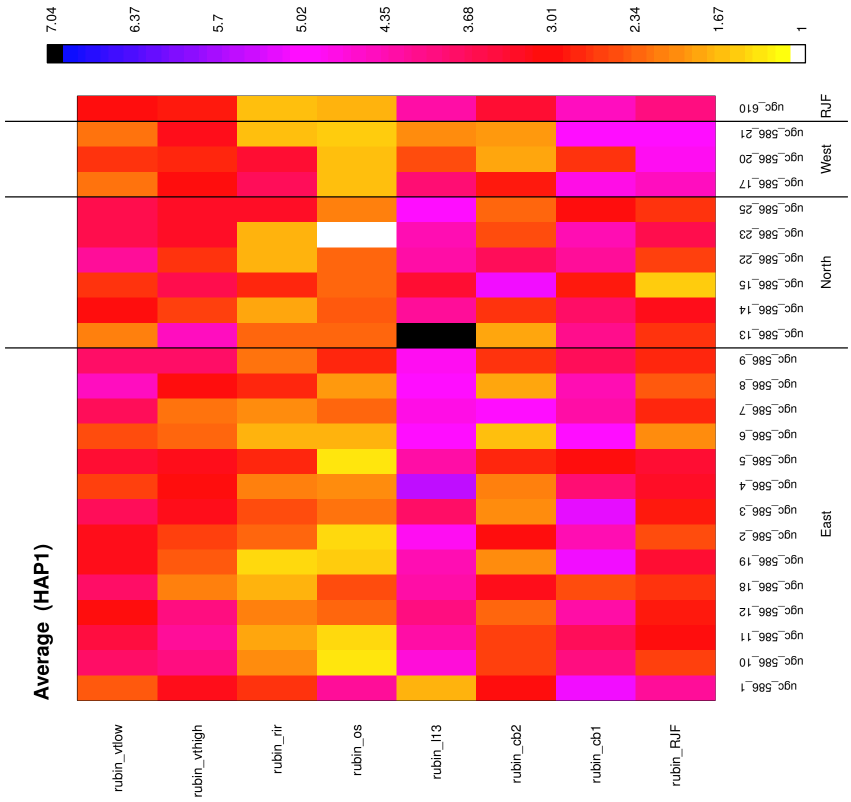

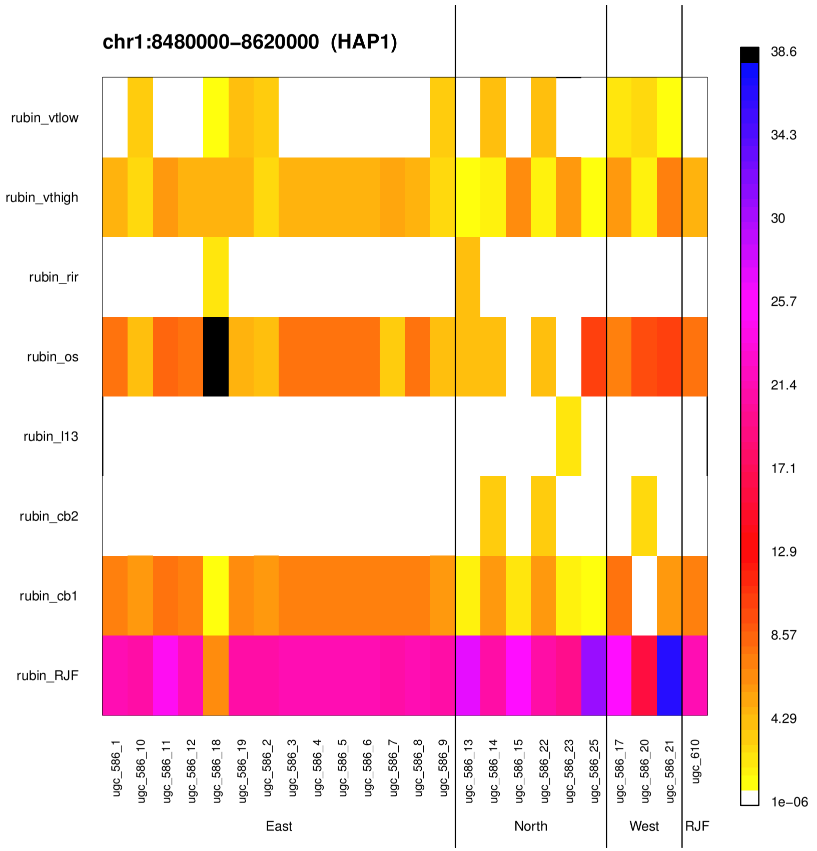
chr1:8480000–8620000 (HAP1)
rubin_vtlow
rubin_vthigh
rubin_rir
rubin_os
rubin_l13
rubin_cb2
rubin_cb1
rubin_RJF
ugc_586_1
ugc_586_10
ugc_586_11
ugc_586_12
ugc_586_18
ugc_586_19
ugc_586_2
ugc_586_3
ugc_586_4
ugc_586_5
ugc_586_6
ugc_586_7
ugc_586_8
ugc_586_9
ugc_586_13
ugc_586_14
ugc_586_15
ugc_586_22
ugc_586_23
ugc_586_25
ugc_586_17
ugc_586_20
ugc_586_21
ugc_610
East
North
West
RJF
38.6
34.3
30
25.7
21.4
17.1
12.9
8.57
4.29
1e–06

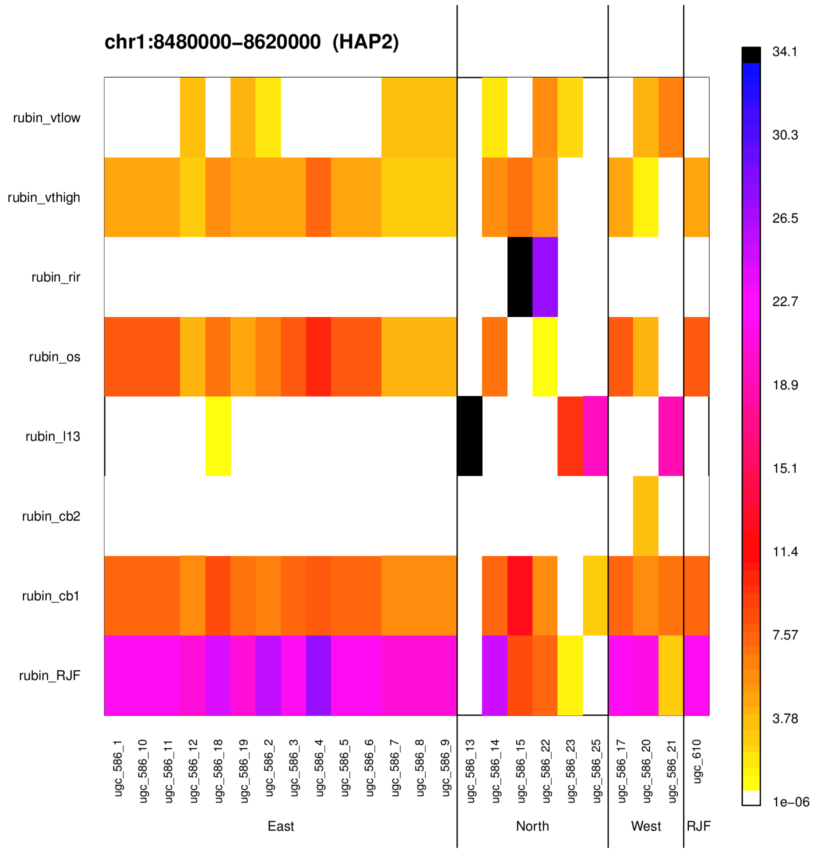
chr1:8480000–8620000 (HAP2)
rubin_vtlow
rubin_vthigh
rubin_rir
rubin_os
rubin_l13
rubin_cb2
rubin_cb1
rubin_RJF
ugc_586_1
ugc_586_10
ugc_586_11
ugc_586_12
ugc_586_18
ugc_586_19
ugc_586_2
ugc_586_3
ugc_586_4
ugc_586_5
ugc_586_6
ugc_586_7
ugc_586_8
ugc_586_9
ugc_586_13
ugc_586_14
ugc_586_15
ugc_586_22
ugc_586_23
ugc_586_25
ugc_586_17
ugc_586_20
ugc_586_21
ugc_610
East
North
West
RJF
34.1
30.3
26.5
22.7
18.9
15.1
11.4
7.57
3.78
1e–06

chr1:147700000–147740000 (HAP2)

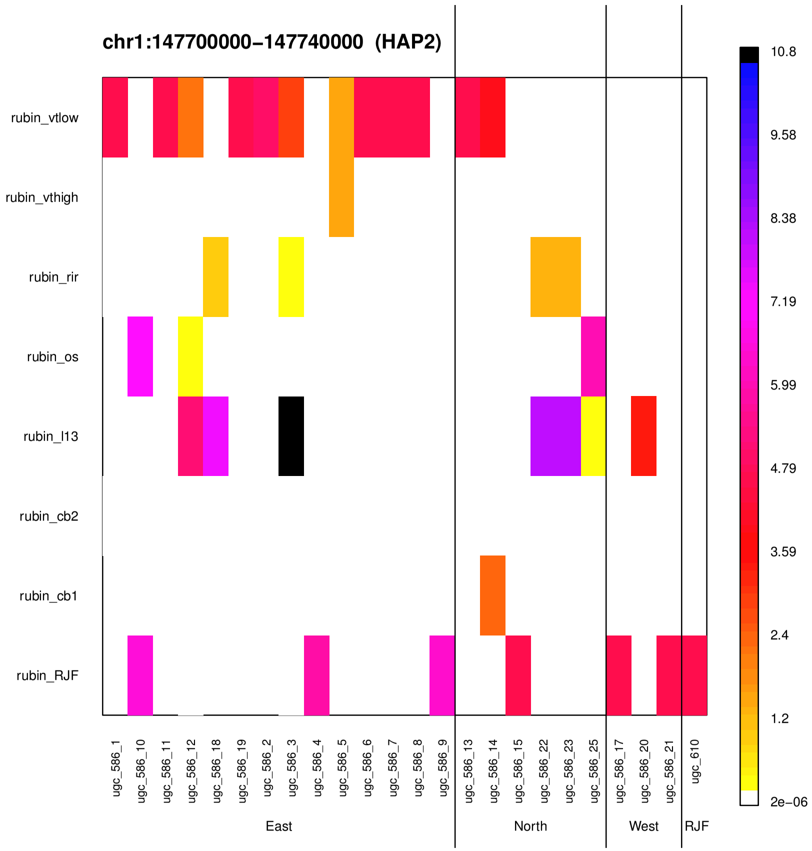

chr1:147700000–147740000 (HAP1)

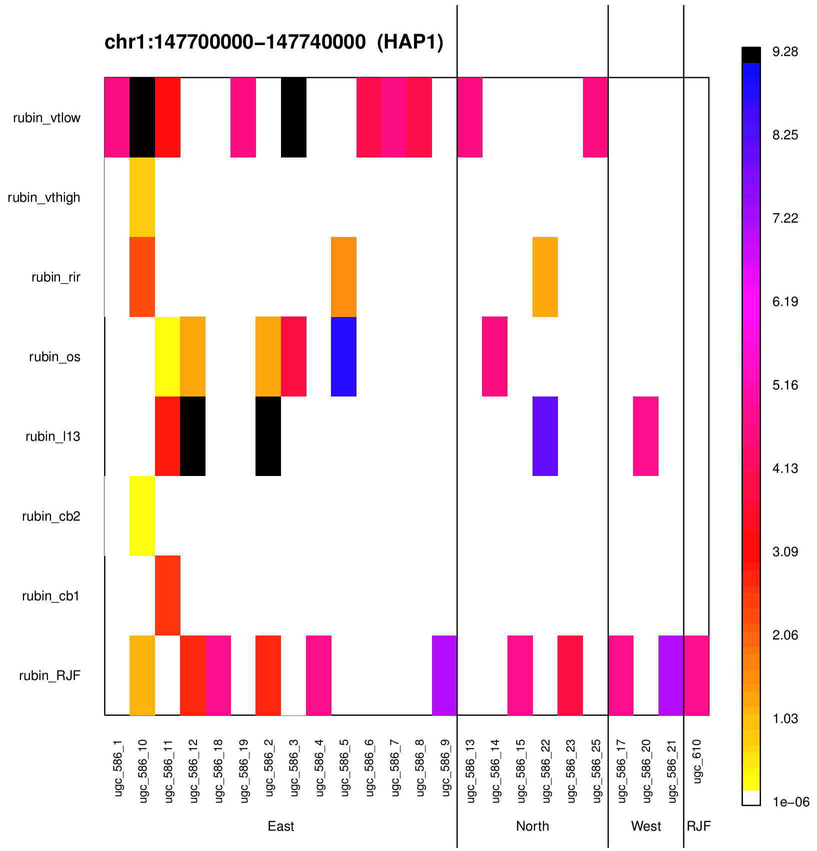

chr1:159880000–159920000 (HAP1)

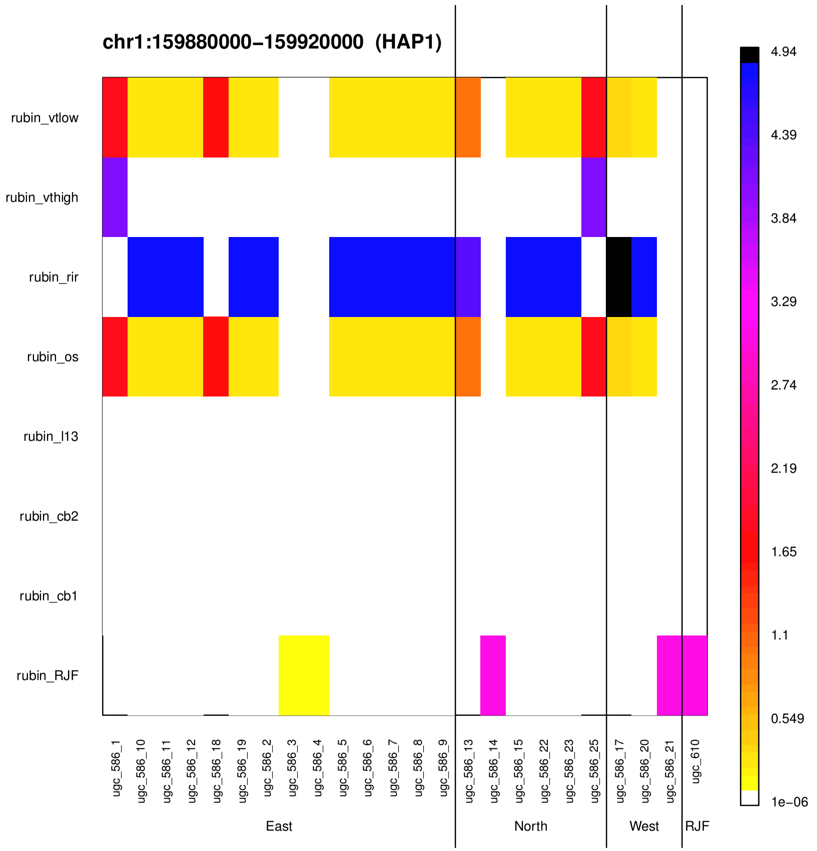

chr1:159880000–159920000 (HAP2)

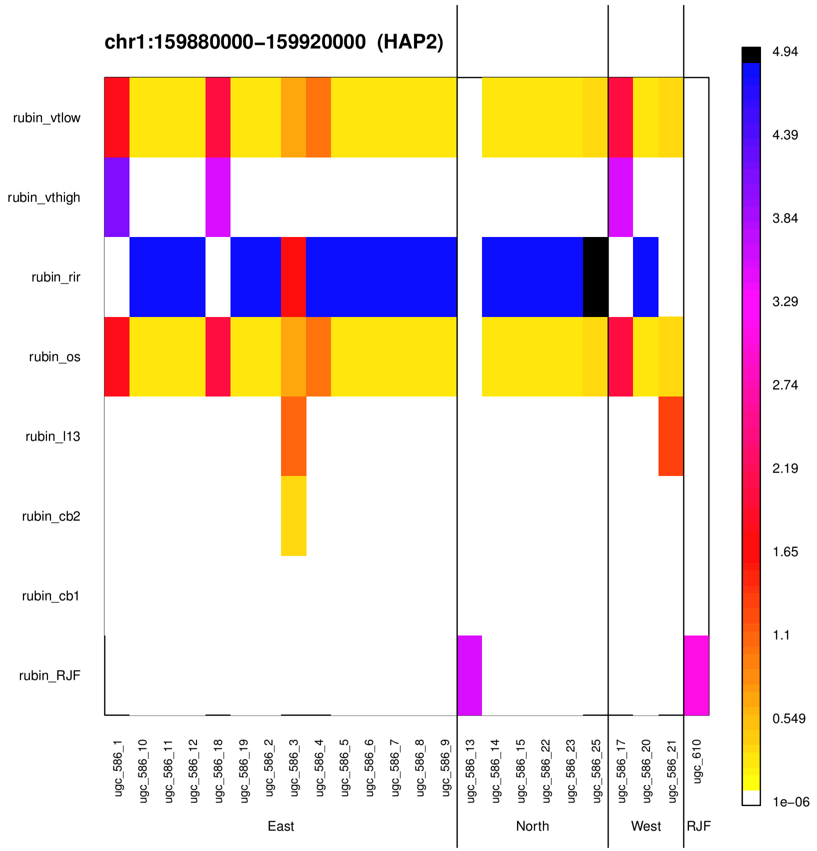

chr1:190580000–190680000 (HAP1)

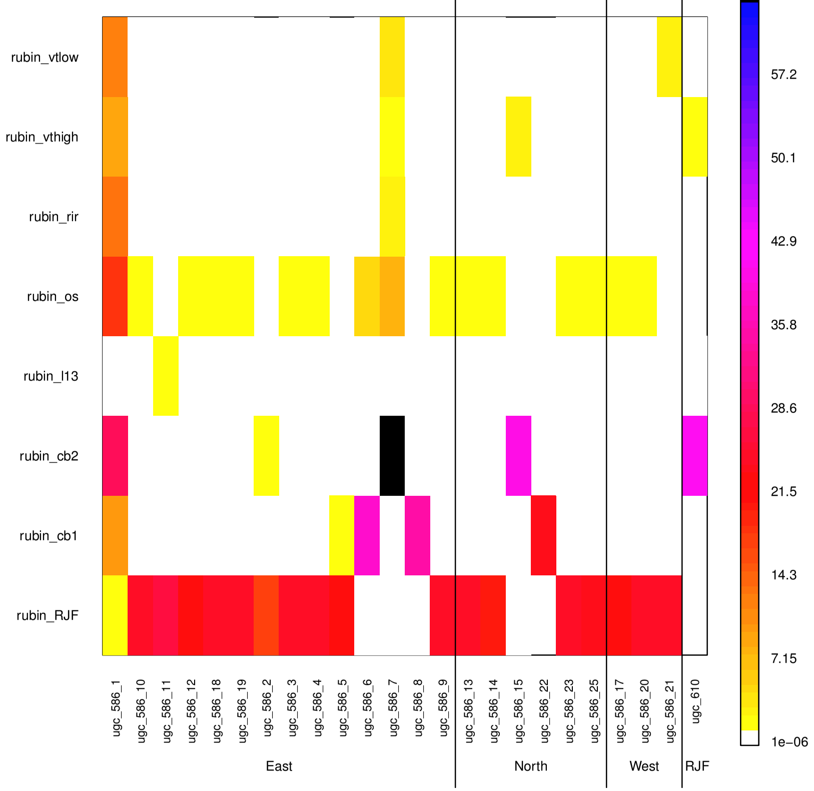

chr1:190580000–190680000 (HAP2)

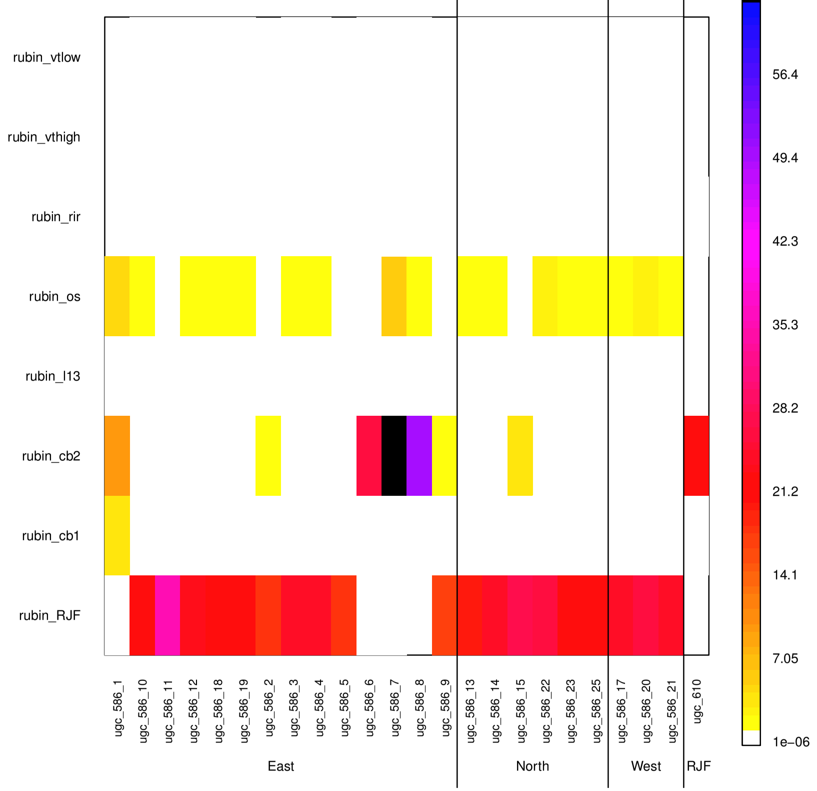

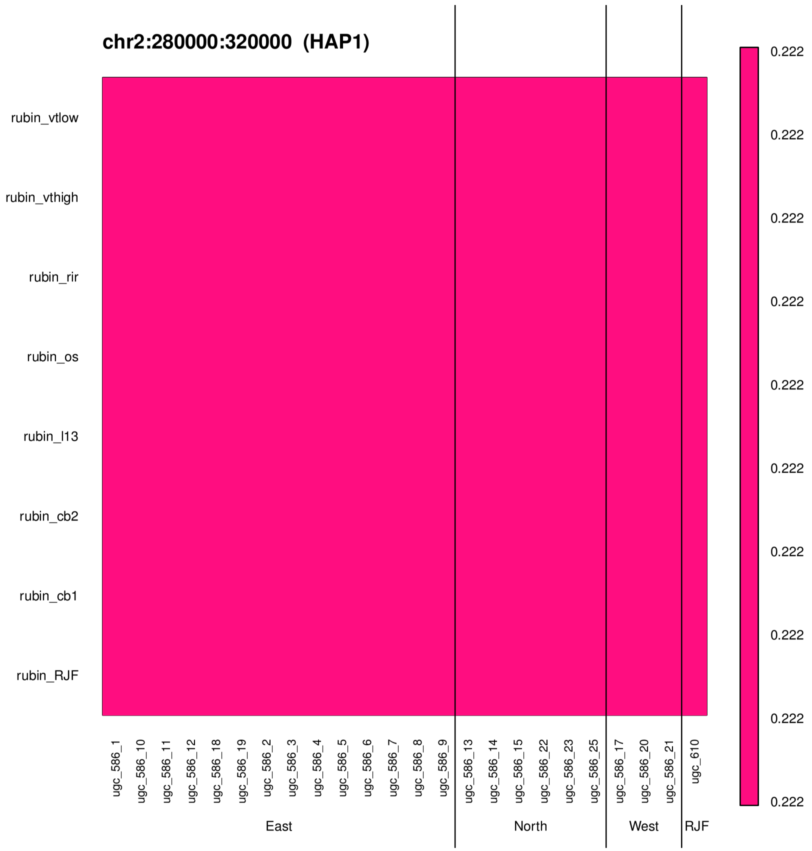

chr2:280000:320000 (HAP1)
rubin_vtlow
rubin_vthigh
rubin_rir
rubin_os
rubin_l13
rubin_cb2
rubin_cb1
rubin_RJF
ugc_586_1
ugc_586_10
ugc_586_11
ugc_586_12
ugc_586_18
ugc_586_19
ugc_586_2
ugc_586_3
ugc_586_4
ugc_586_5
ugc_586_6
ugc_586_7
ugc_586_8
ugc_586_9
ugc_586_13
ugc_586_14
ugc_586_15
ugc_586_22
ugc_586_23
ugc_586_25
ugc_586_17
ugc_586_20
ugc_586_21
ugc_610
East
North
West
RJF
0.222
0.222
0.222
0.222
0.222
0.222
0.222
0.222
0.222
0.222

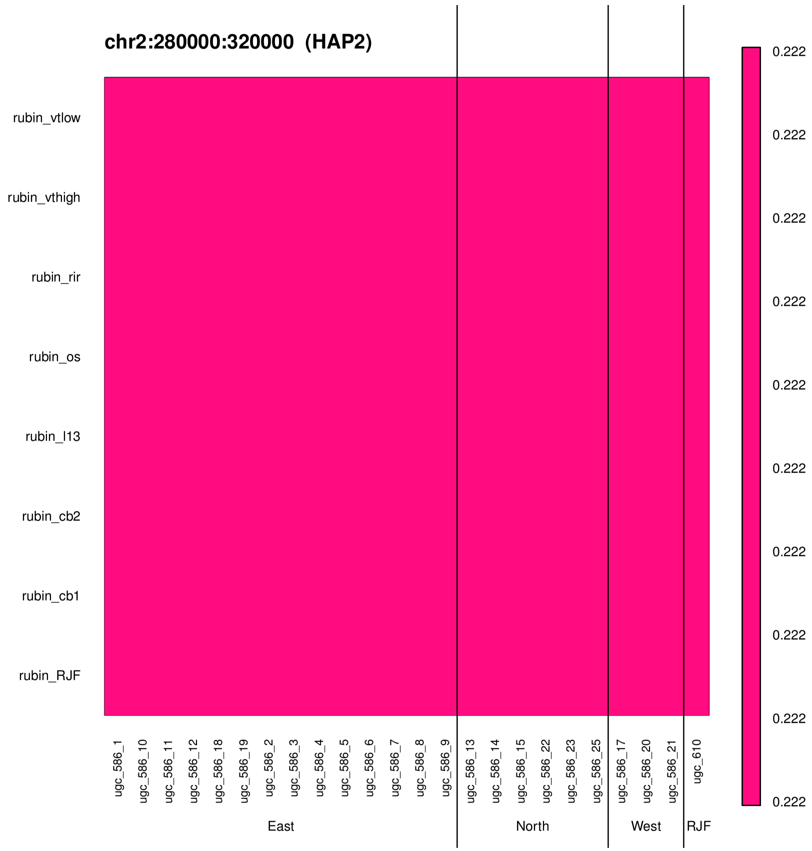

chr2:280000:320000 (HAP2)
rubin_vtlow
rubin_vthigh
rubin_rir
rubin_os
rubin_l13
rubin_cb2
rubin_cb1
rubin_RJF
ugc_586_1
ugc_586_10
ugc_586_11
ugc_586_12
ugc_586_18
ugc_586_19
ugc_586_2
ugc_586_3
ugc_586_4
ugc_586_5
ugc_586_6
ugc_586_7
ugc_586_8
ugc_586_9
ugc_586_13
ugc_586_14
ugc_586_15
ugc_586_22
ugc_586_23
ugc_586_25
ugc_586_17
ugc_586_20
ugc_586_21
ugc_610
East
North
West
RJF
0.222
0.222
0.222
0.222
0.222
0.222
0.222
0.222
0.222
0.222

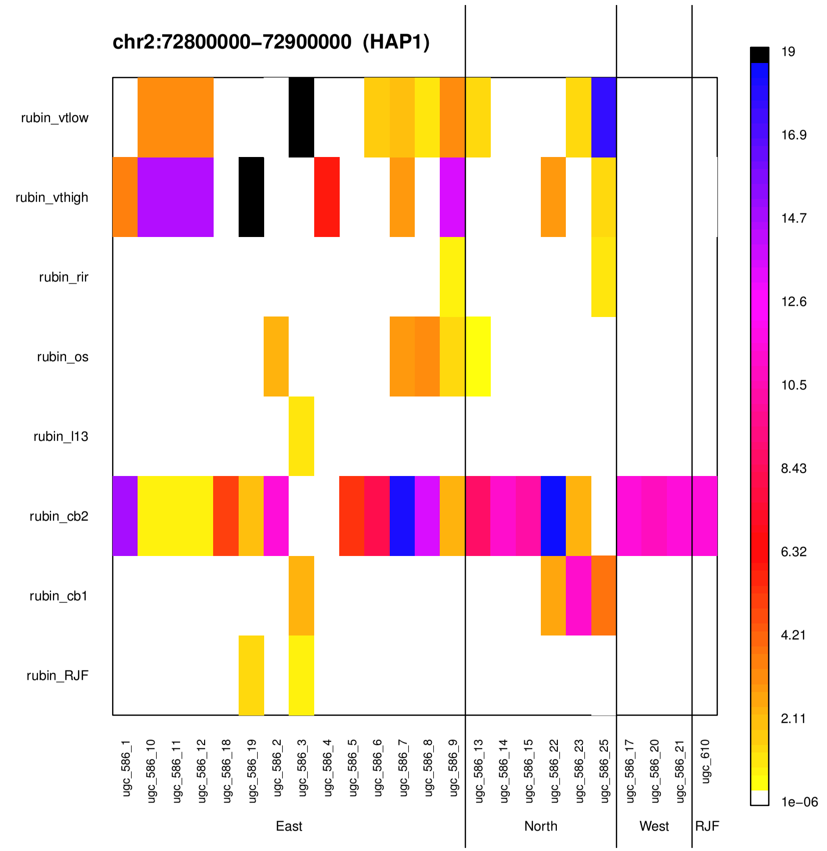
chr2:72800000–72900000 (HAP1)
rubin_vtlow
rubin_vthigh
rubin_rir
rubin_os
rubin_l13
rubin_cb2
rubin_cb1
rubin_RJF
ugc_586_1
ugc_586_10
ugc_586_11
ugc_586_12
ugc_586_18
ugc_586_19
ugc_586_2
ugc_586_3
ugc_586_4
ugc_586_5
ugc_586_6
ugc_586_7
ugc_586_8
ugc_586_9
ugc_586_13
ugc_586_14
ugc_586_15
ugc_586_22
ugc_586_23
ugc_586_25
ugc_586_17
ugc_586_20
ugc_586_21
ugc_610
East
North
West
RJF
19
16.9
14.7
12.6
10.5
8.43
6.32
4.21
2.11
1e–06

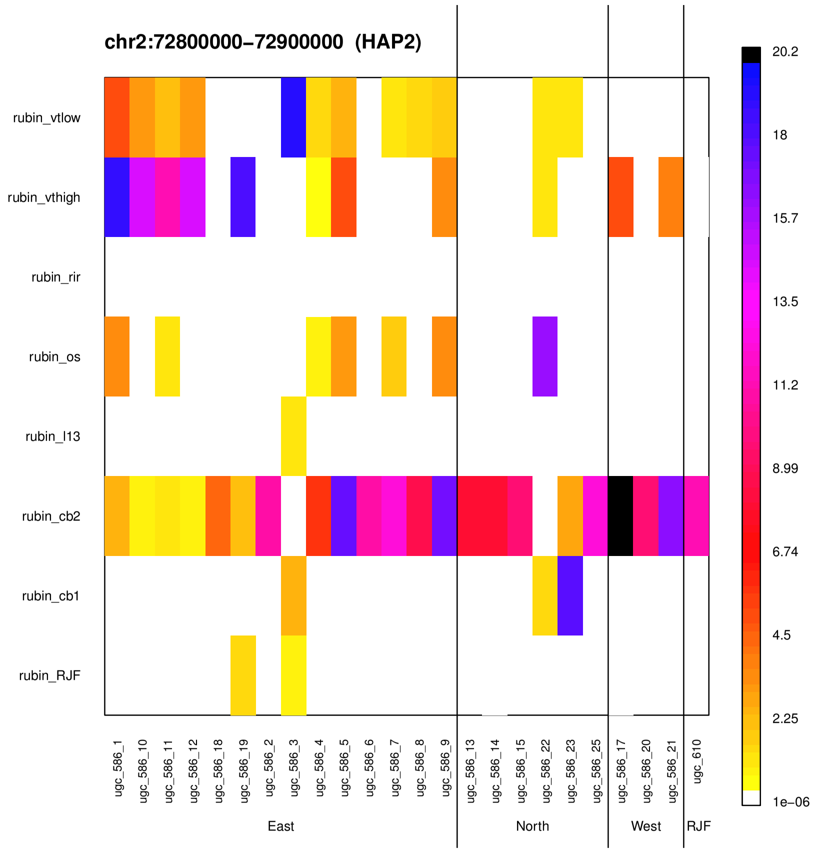
chr2:72800000–72900000 (HAP2)
rubin_vtlow
rubin_vthigh
rubin_rir
rubin_os
rubin_l13
rubin_cb2
rubin_cb1
rubin_RJF
ugc_586_1
ugc_586_10
ugc_586_11
ugc_586_12
ugc_586_18
ugc_586_19
ugc_586_2
ugc_586_3
ugc_586_4
ugc_586_5
ugc_586_6
ugc_586_7
ugc_586_8
ugc_586_9
ugc_586_13
ugc_586_14
ugc_586_15
ugc_586_22
ugc_586_23
ugc_586_25
ugc_586_17
ugc_586_20
ugc_586_21
ugc_610
East
North
West
RJF
20.2
18
15.7
13.5
11.2
8.99
6.74
4.5
2.25
1e–06

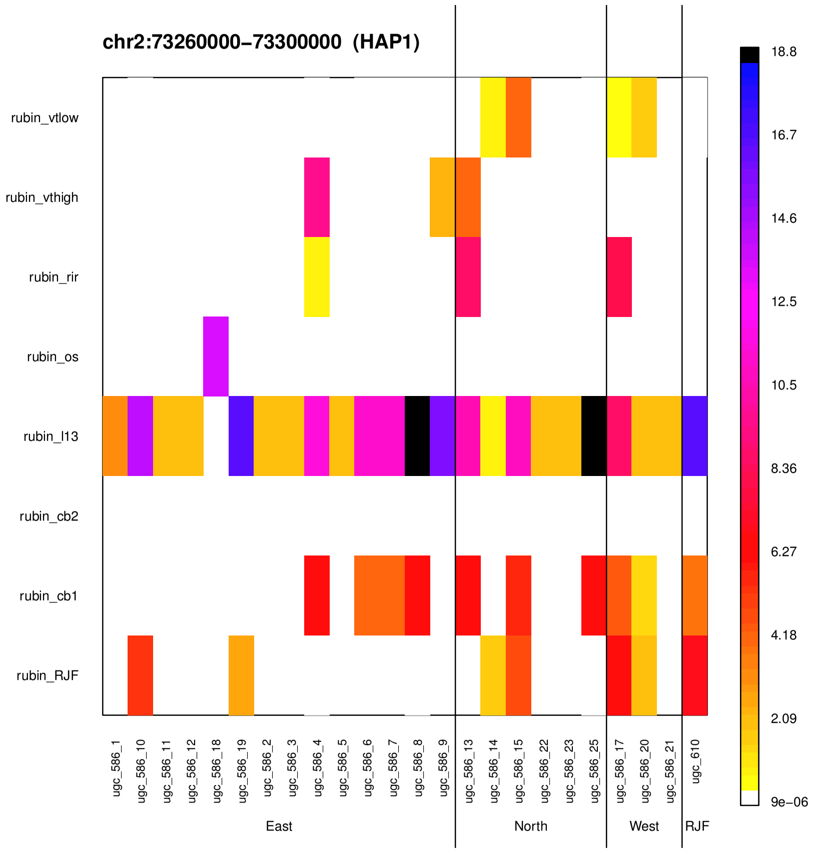
chr2:73260000−73300000 (HAP1)
rubin_vtlow
rubin_vthigh
rubin_rir
rubin_os
rubin_l13
rubin_cb2
rubin_cb1
rubin_RJF
ugc_586_1
ugc_586_10
ugc_586_11
ugc_586_12
ugc_586_18
ugc_586_19
ugc_586_2
ugc_586_3
ugc_586_4
ugc_586_5
ugc_586_6
ugc_586_7
ugc_586_8
ugc_586_9
ugc_586_13
ugc_586_14
ugc_586_15
ugc_586_22
ugc_586_23
ugc_586_25
ugc_586_17
ugc_586_20
ugc_586_21
ugc_610
East
North
West
RJF
18.8
16.7
14.6
12.5
10.5
8.36
6.27
4.18
2.09
9e−06

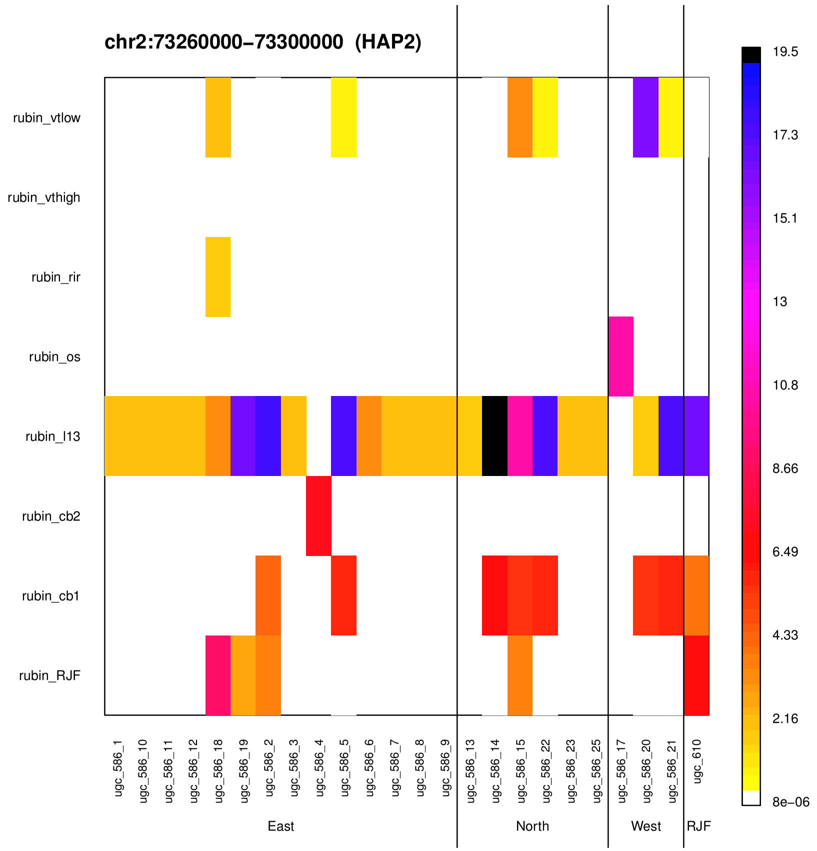
chr2:73260000−73300000 (HAP2)
rubin_vtlow
rubin_vthigh
rubin_rir
rubin_os
rubin_l13
rubin_cb2
rubin_cb1
rubin_RJF
ugc_586_1
ugc_586_10
ugc_586_11
ugc_586_12
ugc_586_18
ugc_586_19
ugc_586_2
ugc_586_3
ugc_586_4
ugc_586_5
ugc_586_6
ugc_586_7
ugc_586_8
ugc_586_9
ugc_586_13
ugc_586_14
ugc_586_15
ugc_586_22
ugc_586_23
ugc_586_25
ugc_586_17
ugc_586_20
ugc_586_21
ugc_610
East
North
West
RJF
19.5
17.3
15.1
13
10.8
8.66
6.49
4.33
2.16
8e−06

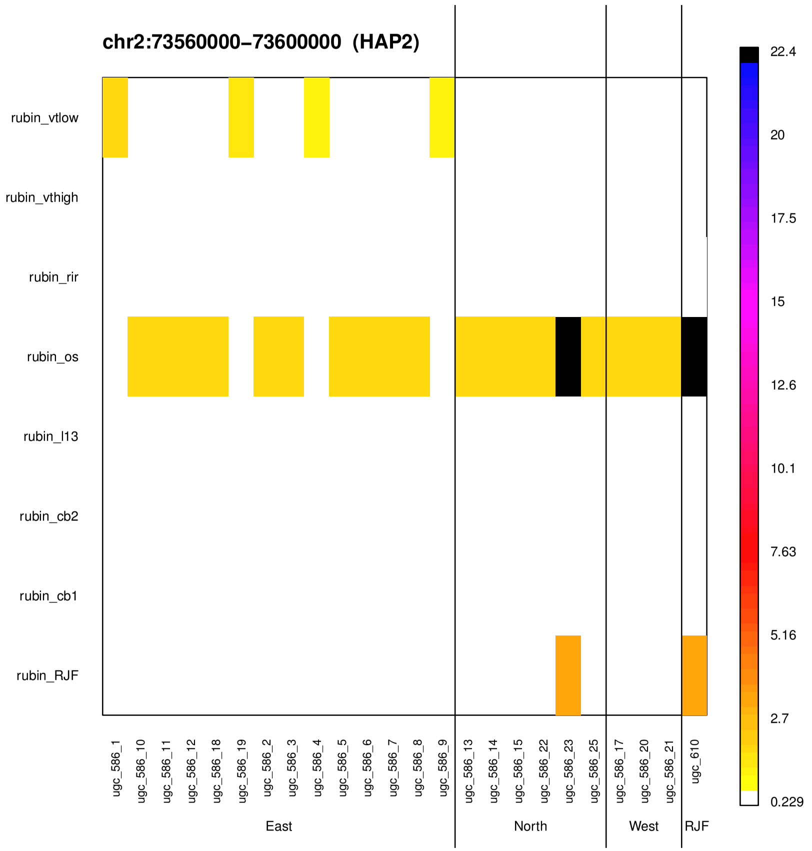
chr2:73560000-73600000 (HAP2)
rubin_vtlow
rubin_vthigh
rubin_rir
rubin_os
rubin_l13
rubin_cb2
rubin_cb1
rubin_RJF
ugc_586_1
ugc_586_10
ugc_586_11
ugc_586_12
ugc_586_18
ugc_586_19
ugc_586_2
ugc_586_3
ugc_586_4
ugc_586_5
ugc_586_6
ugc_586_7
ugc_586_8
ugc_586_9
ugc_586_13
ugc_586_14
ugc_586_15
ugc_586_22
ugc_586_23
ugc_586_25
ugc_586_17
ugc_586_20
ugc_586_21
ugc_610
East
North
West
RJF
22.4
20
17.5
15
12.6
10.1
7.63
5.16
2.7
0.229

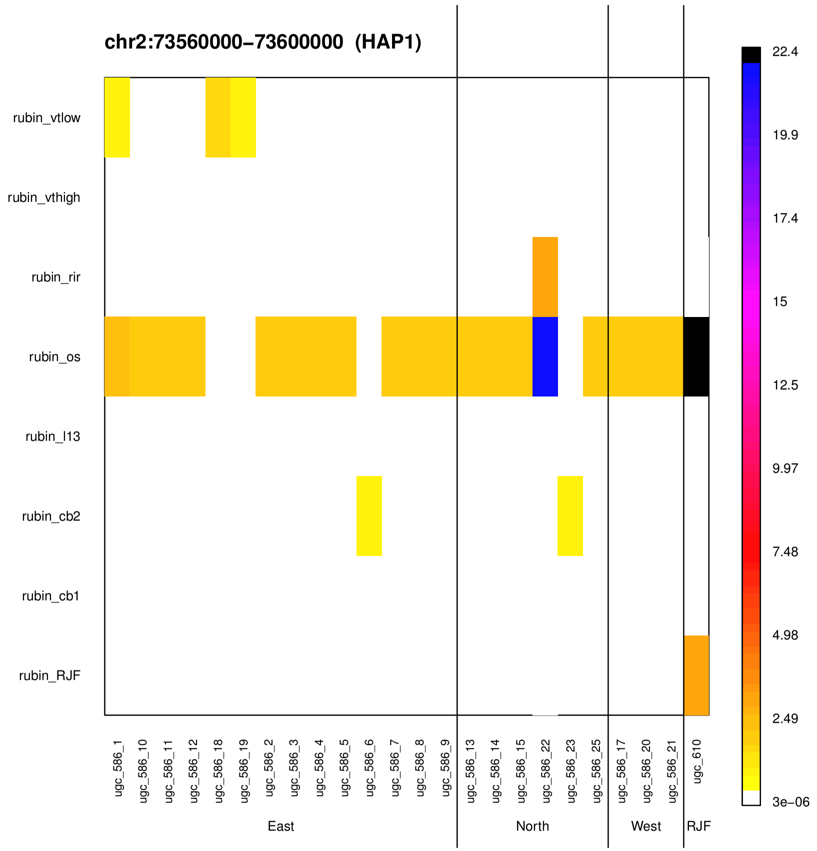
chr2:73560000-73600000 (HAP1)
rubin_vtlow
rubin_vthigh
rubin_rir
rubin_os
rubin_l13
rubin_cb2
rubin_cb1
rubin_RJF
ugc_586_1
ugc_586_10
ugc_586_11
ugc_586_12
ugc_586_18
ugc_586_19
ugc_586_2
ugc_586_3
ugc_586_4
ugc_586_5
ugc_586_6
ugc_586_7
ugc_586_8
ugc_586_9
ugc_586_13
ugc_586_14
ugc_586_15
ugc_586_22
ugc_586_23
ugc_586_25
ugc_586_17
ugc_586_20
ugc_586_21
ugc_610
East
North
West
RJF
22.4
19.9
17.4
15
12.5
9.97
7.48
4.98
2.49
3e-06

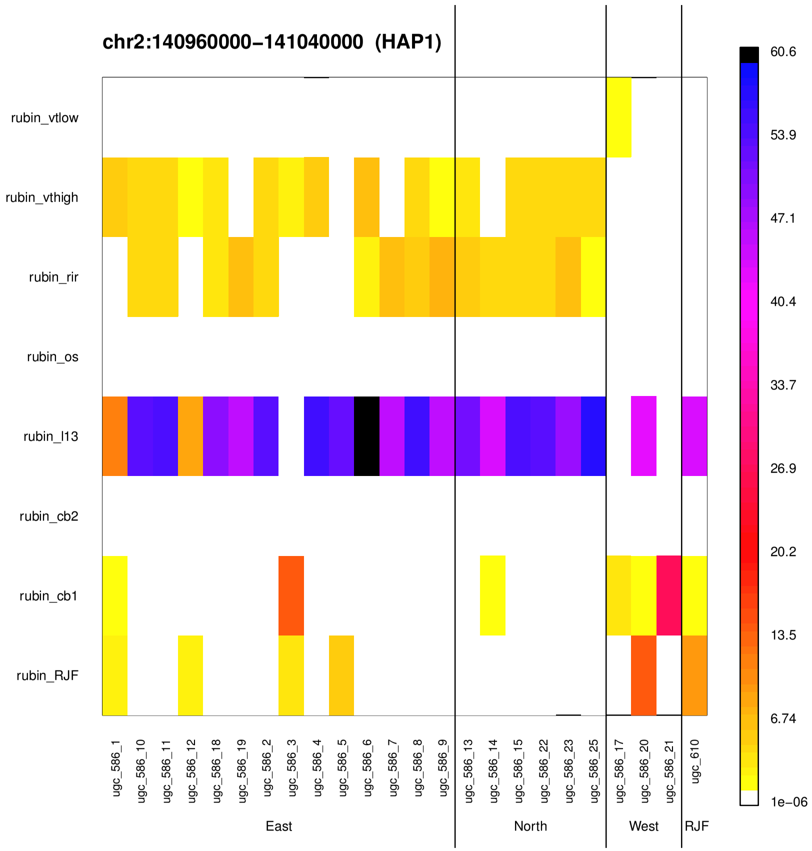
chr2:140960000–141040000 (HAP1)
rubin_vtlow
rubin_vthigh
rubin_rir
rubin_os
rubin_l13
rubin_cb2
rubin_cb1
rubin_RJF
ugc_586_1
ugc_586_10
ugc_586_11
ugc_586_12
ugc_586_18
ugc_586_19
ugc_586_2
ugc_586_3
ugc_586_4
ugc_586_5
ugc_586_6
ugc_586_7
ugc_586_8
ugc_586_9
ugc_586_13
ugc_586_14
ugc_586_15
ugc_586_22
ugc_586_23
ugc_586_25
ugc_586_17
ugc_586_20
ugc_586_21
ugc_610
East
North
West
RJF
60.6
53.9
47.1
40.4
33.7
26.9
20.2
13.5
6.74
1e−06

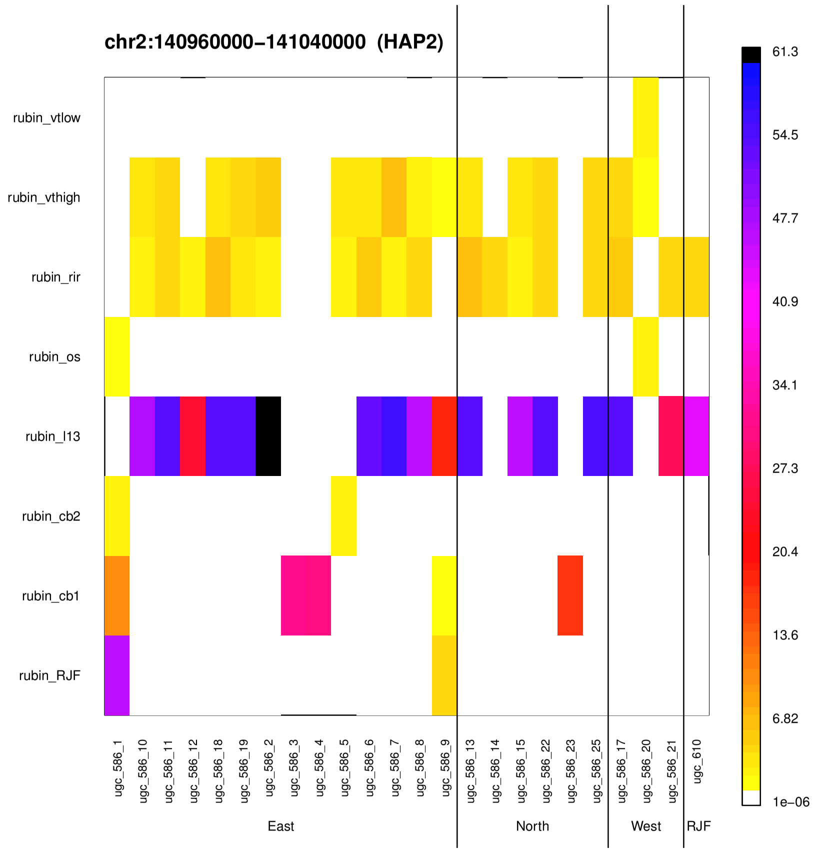
chr2:140960000–141040000 (HAP2)
rubin_vtlow
rubin_vthigh
rubin_rir
rubin_os
rubin_l13
rubin_cb2
rubin_cb1
rubin_RJF
ugc_586_1
ugc_586_10
ugc_586_11
ugc_586_12
ugc_586_18
ugc_586_19
ugc_586_2
ugc_586_3
ugc_586_4
ugc_586_5
ugc_586_6
ugc_586_7
ugc_586_8
ugc_586_9
ugc_586_13
ugc_586_14
ugc_586_15
ugc_586_22
ugc_586_23
ugc_586_25
ugc_586_17
ugc_586_20
ugc_586_21
ugc_610
East
North
West
RJF
61.3
54.5
47.7
40.9
34.1
27.3
20.4
13.6
6.82
1e−06

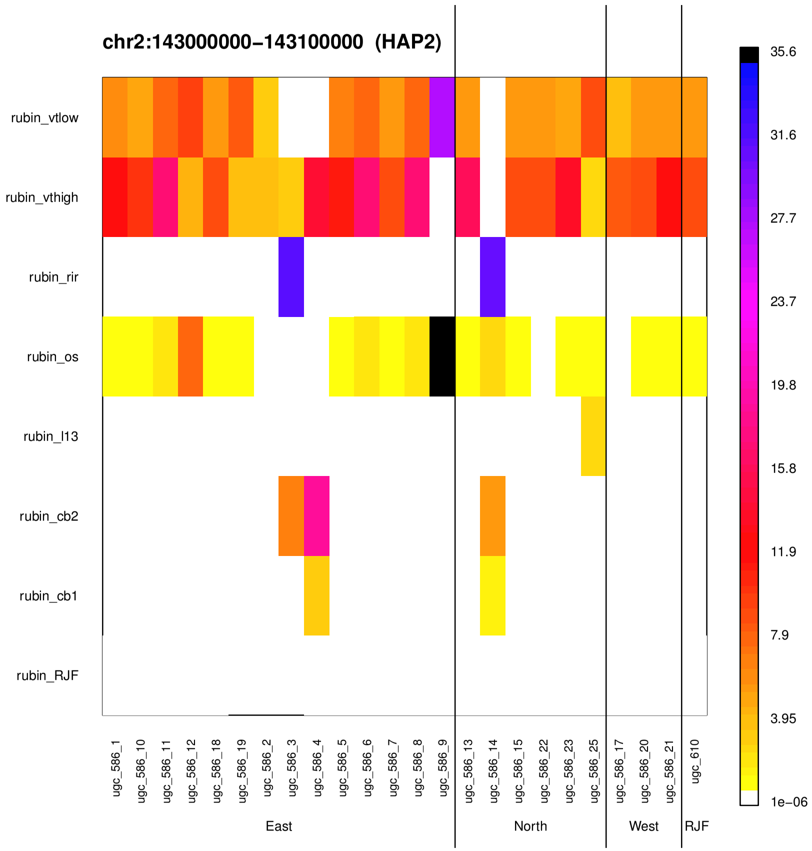
chr2:143000000–143100000 (HAP2)
rubin_vtlow
rubin_vthigh
rubin_rir
rubin_os
rubin_l13
rubin_cb2
rubin_cb1
rubin_RJF
ugc_586_1
ugc_586_10
ugc_586_11
ugc_586_12
ugc_586_18
ugc_586_19
ugc_586_2
ugc_586_3
ugc_586_4
ugc_586_5
ugc_586_6
ugc_586_7
ugc_586_8
ugc_586_9
ugc_586_13
ugc_586_14
ugc_586_15
ugc_586_22
ugc_586_23
ugc_586_25
ugc_586_17
ugc_586_20
ugc_586_21
ugc_610
East
North
West
RJF
35.6
31.6
27.7
23.7
19.8
15.8
11.9
7.9
3.95
1e–06

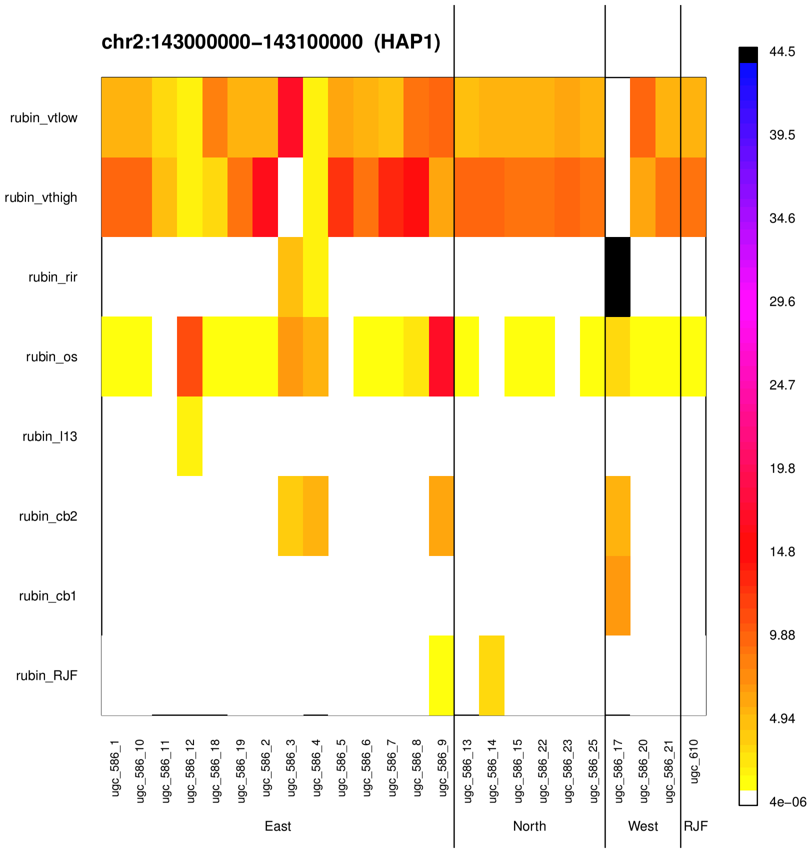
chr2:143000000–143100000 (HAP1)
rubin_vtlow
rubin_vthigh
rubin_rir
rubin_os
rubin_l13
rubin_cb2
rubin_cb1
rubin_RJF
ugc_586_1
ugc_586_10
ugc_586_11
ugc_586_12
ugc_586_18
ugc_586_19
ugc_586_2
ugc_586_3
ugc_586_4
ugc_586_5
ugc_586_6
ugc_586_7
ugc_586_8
ugc_586_9
ugc_586_13
ugc_586_14
ugc_586_15
ugc_586_22
ugc_586_23
ugc_586_25
ugc_586_17
ugc_586_20
ugc_586_21
ugc_610
East
North
West
RJF
44.5
39.5
34.6
29.6
24.7
19.8
14.8
9.88
4.94
4e–06

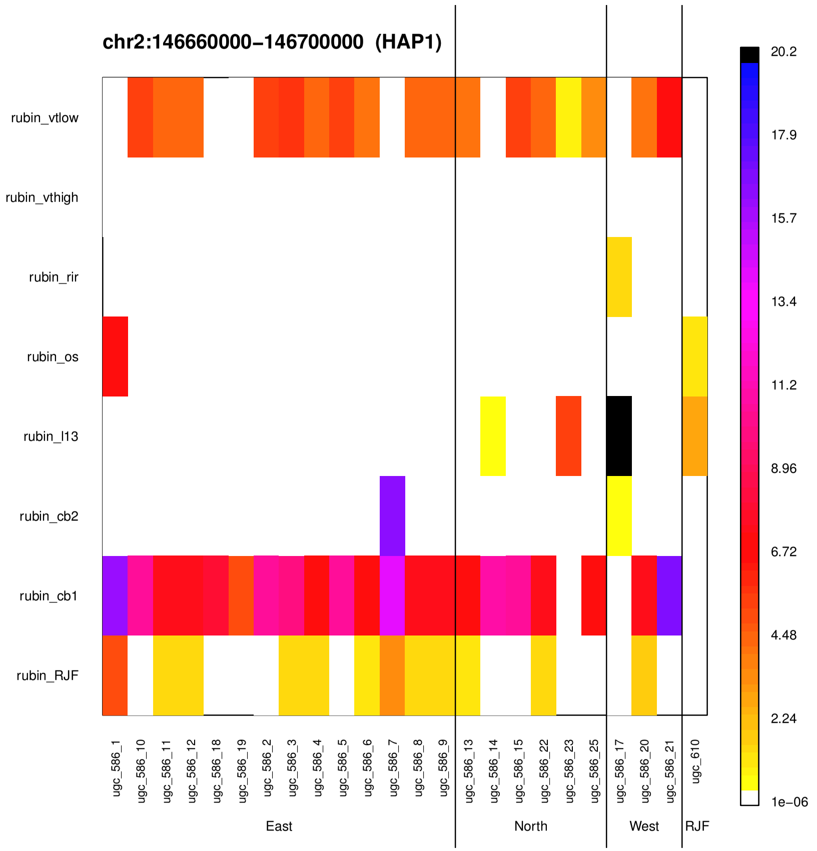
chr2:146660000–146700000 (HAP1)
rubin_vtlow
rubin_vthigh
rubin_rir
rubin_os
rubin_l13
rubin_cb2
rubin_cb1
rubin_RJF
ugc_586_1
ugc_586_10
ugc_586_11
ugc_586_12
ugc_586_18
ugc_586_19
ugc_586_2
ugc_586_3
ugc_586_4
ugc_586_5
ugc_586_6
ugc_586_7
ugc_586_8
ugc_586_9
ugc_586_13
ugc_586_14
ugc_586_15
ugc_586_22
ugc_586_23
ugc_586_25
ugc_586_17
ugc_586_20
ugc_586_21
ugc_610
East
North
West
RJF
20.2
17.9
15.7
13.4
11.2
8.96
6.72
4.48
2.24
1e−06

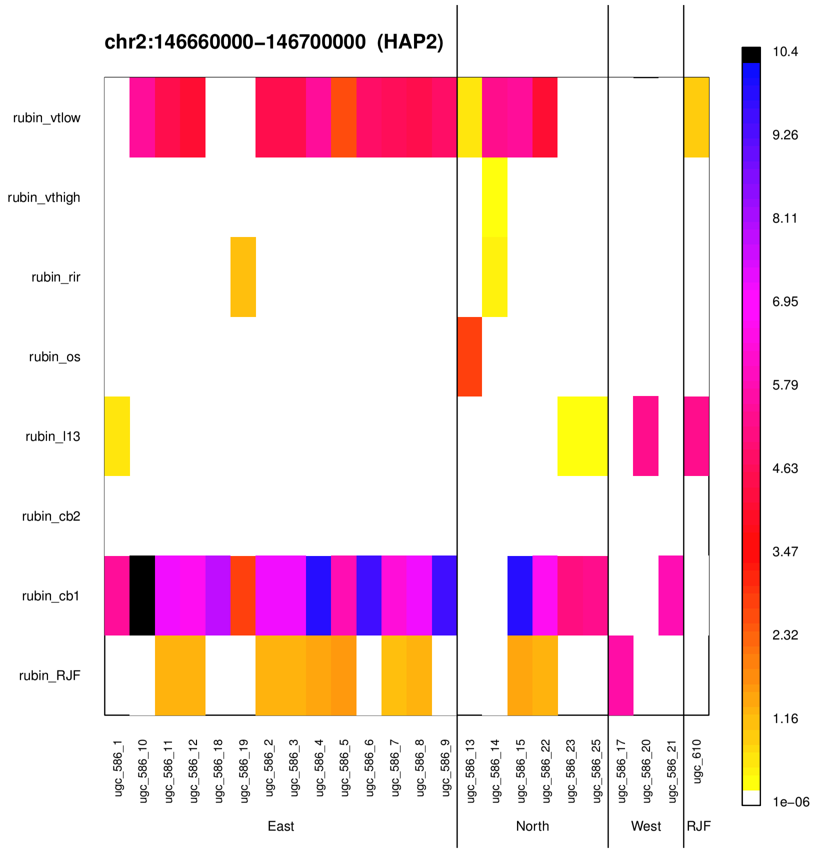
chr2:146660000–146700000 (HAP2)
rubin_vtlow
rubin_vthigh
rubin_rir
rubin_os
rubin_l13
rubin_cb2
rubin_cb1
rubin_RJF
ugc_586_1
ugc_586_10
ugc_586_11
ugc_586_12
ugc_586_18
ugc_586_19
ugc_586_2
ugc_586_3
ugc_586_4
ugc_586_5
ugc_586_6
ugc_586_7
ugc_586_8
ugc_586_9
ugc_586_13
ugc_586_14
ugc_586_15
ugc_586_22
ugc_586_23
ugc_586_25
ugc_586_17
ugc_586_20
ugc_586_21
ugc_610
East
North
West
RJF
10.4
9.26
8.11
6.95
5.79
4.63
3.47
2.32
1.16
1e−06

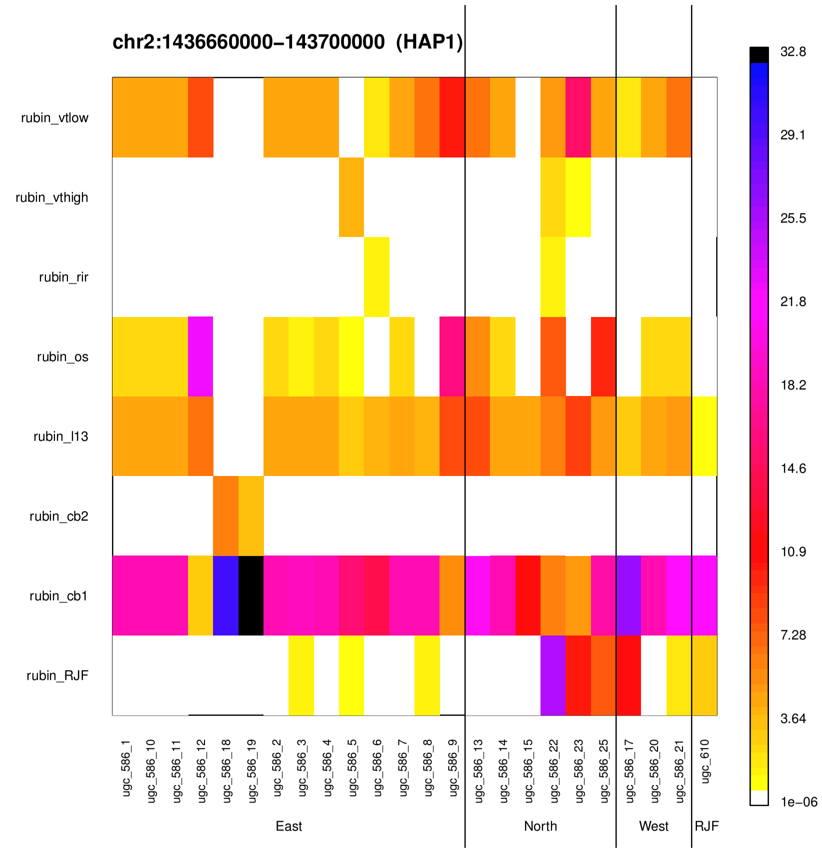
chr2:1436660000–143700000 (HAP1)
rubin_vtlow
rubin_vthigh
rubin_rir
rubin_os
rubin_l13
rubin_cb2
rubin_cb1
rubin_RJF
ugc_586_1
ugc_586_10
ugc_586_11
ugc_586_12
ugc_586_18
ugc_586_19
ugc_586_2
ugc_586_3
ugc_586_4
ugc_586_5
ugc_586_6
ugc_586_7
ugc_586_8
ugc_586_9
ugc_586_13
ugc_586_14
ugc_586_15
ugc_586_22
ugc_586_23
ugc_586_25
ugc_586_17
ugc_586_20
ugc_586_21
ugc_610
East
North
West
RJF
32.8
29.1
25.5
21.8
18.2
14.6
10.9
7.28
3.64
1e–06

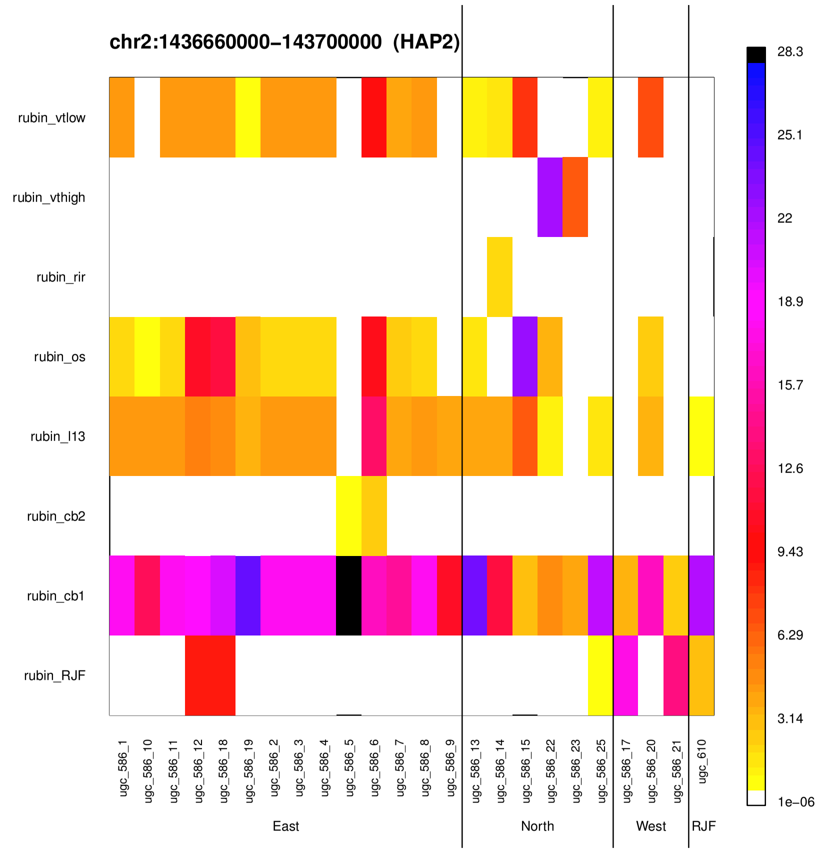
chr2:1436660000–143700000 (HAP2)
rubin_vtlow
rubin_vthigh
rubin_rir
rubin_os
rubin_l13
rubin_cb2
rubin_cb1
rubin_RJF
ugc_586_1
ugc_586_10
ugc_586_11
ugc_586_12
ugc_586_18
ugc_586_19
ugc_586_2
ugc_586_3
ugc_586_4
ugc_586_5
ugc_586_6
ugc_586_7
ugc_586_8
ugc_586_9
ugc_586_13
ugc_586_14
ugc_586_15
ugc_586_22
ugc_586_23
ugc_586_25
ugc_586_17
ugc_586_20
ugc_586_21
ugc_610
East
North
West
RJF
28.3
25.1
22
18.9
15.7
12.6
9.43
6.29
3.14
1e–06

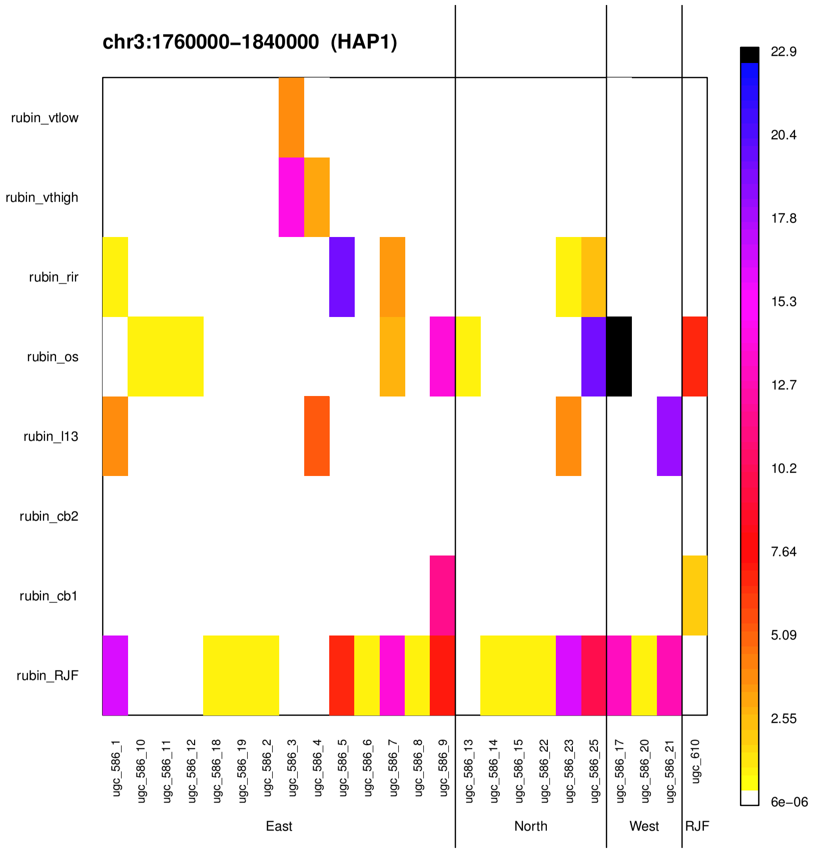
chr3:1760000–1840000 (HAP1)
rubin_vtlow
rubin_vthigh
rubin_rir
rubin_os
rubin_l13
rubin_cb2
rubin_cb1
rubin_RJF
ugc_586_1
ugc_586_10
ugc_586_11
ugc_586_12
ugc_586_18
ugc_586_19
ugc_586_2
ugc_586_3
ugc_586_4
ugc_586_5
ugc_586_6
ugc_586_7
ugc_586_8
ugc_586_9
ugc_586_13
ugc_586_14
ugc_586_15
ugc_586_22
ugc_586_23
ugc_586_25
ugc_586_17
ugc_586_20
ugc_586_21
ugc_610
East
North
West
RJF
22.9
20.4
17.8
15.3
12.7
10.2
7.64
5.09
2.55
6e–06

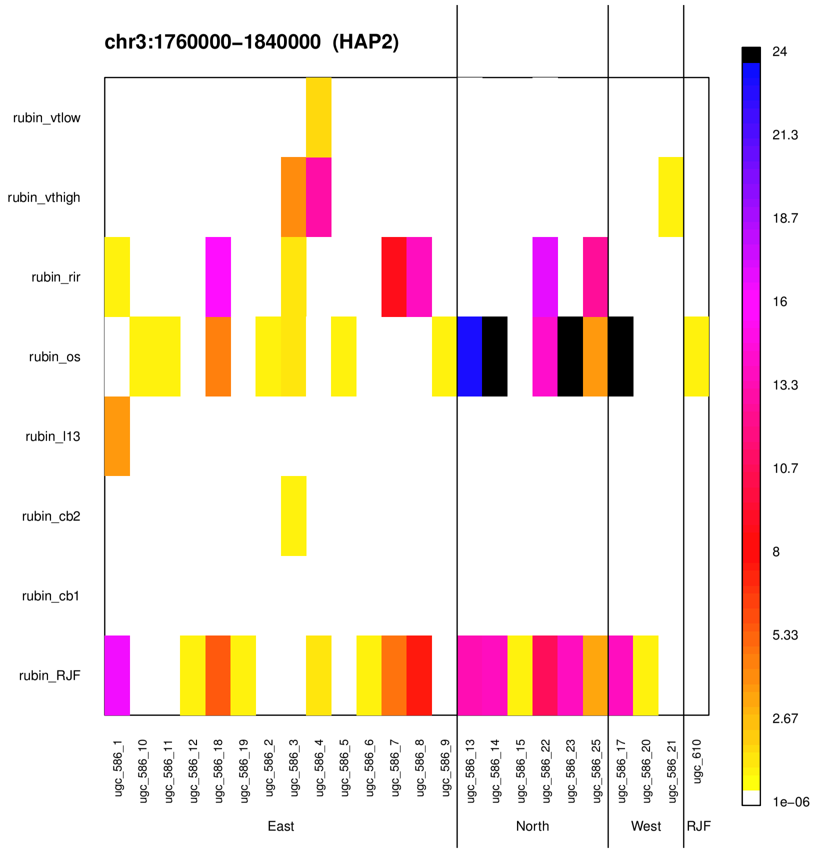
chr3:1760000–1840000 (HAP2)
rubin_vtlow
rubin_vthigh
rubin_rir
rubin_os
rubin_l13
rubin_cb2
rubin_cb1
rubin_RJF
ugc_586_1
ugc_586_10
ugc_586_11
ugc_586_12
ugc_586_18
ugc_586_19
ugc_586_2
ugc_586_3
ugc_586_4
ugc_586_5
ugc_586_6
ugc_586_7
ugc_586_8
ugc_586_9
ugc_586_13
ugc_586_14
ugc_586_15
ugc_586_22
ugc_586_23
ugc_586_25
ugc_586_17
ugc_586_20
ugc_586_21
ugc_610
East
North
West
RJF
24
21.3
18.7
16
13.3
10.7
8
5.33
2.67
1e–06

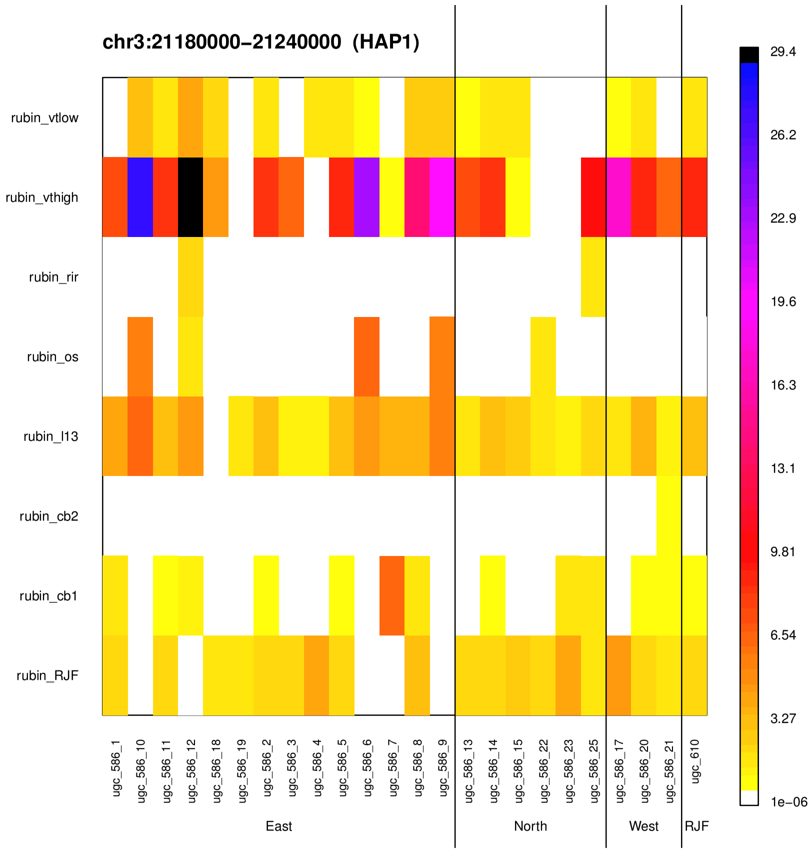
chr3:21180000-21240000 (HAP1)
rubin_vtlow
rubin_vthigh
rubin_rir
rubin_os
rubin_l13
rubin_cb2
rubin_cb1
rubin_RJF
ugc_586_1
ugc_586_10
ugc_586_11
ugc_586_12
ugc_586_18
ugc_586_19
ugc_586_2
ugc_586_3
ugc_586_4
ugc_586_5
ugc_586_6
ugc_586_7
ugc_586_8
ugc_586_9
ugc_586_13
ugc_586_14
ugc_586_15
ugc_586_22
ugc_586_23
ugc_586_25
ugc_586_17
ugc_586_20
ugc_586_21
ugc_610
East
North
West
RJF
29.4
26.2
22.9
19.6
16.3
13.1
9.81
6.54
3.27
1e-06

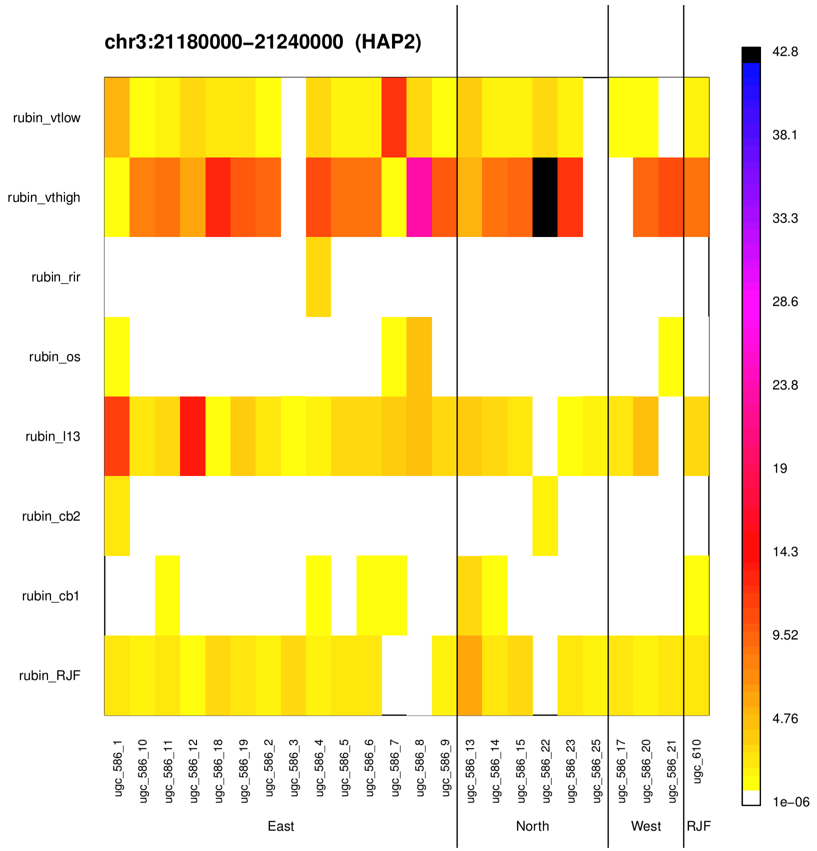
chr3:21180000-21240000 (HAP2)
rubin_vtlow
rubin_vthigh
rubin_rir
rubin_os
rubin_l13
rubin_cb2
rubin_cb1
rubin_RJF
ugc_586_1
ugc_586_10
ugc_586_11
ugc_586_12
ugc_586_18
ugc_586_19
ugc_586_2
ugc_586_3
ugc_586_4
ugc_586_5
ugc_586_6
ugc_586_7
ugc_586_8
ugc_586_9
ugc_586_13
ugc_586_14
ugc_586_15
ugc_586_22
ugc_586_23
ugc_586_25
ugc_586_17
ugc_586_20
ugc_586_21
ugc_610
East
North
West
RJF
42.8
38.1
33.3
28.6
23.8
19
14.3
9.52
4.76
1e-06

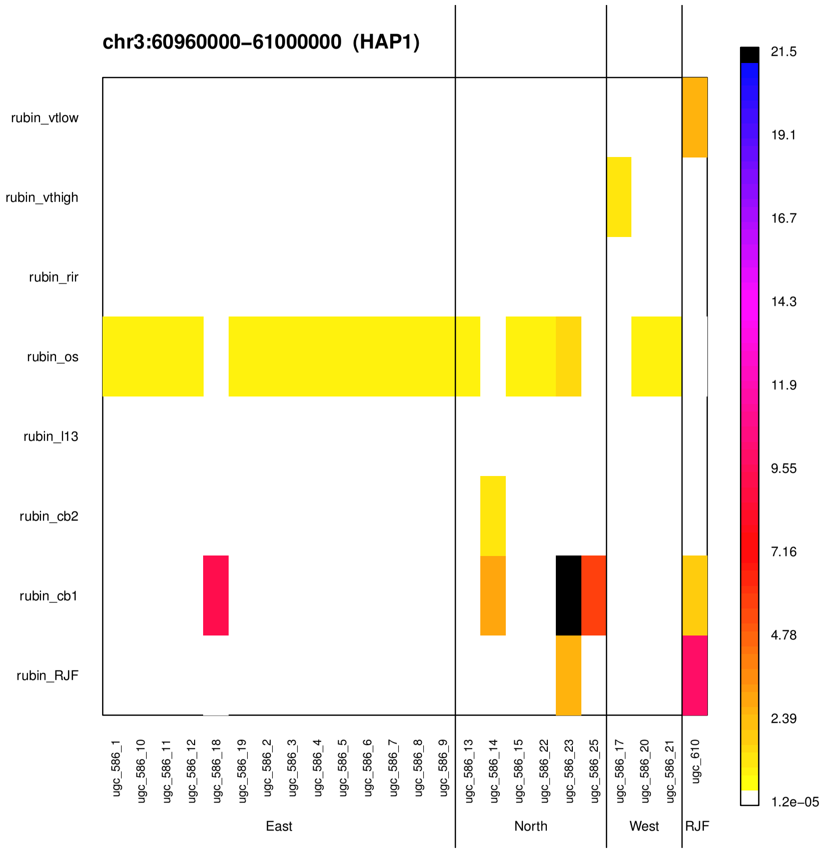
chr3:60960000–61000000 (HAP1)
rubin_vtlow
rubin_vthigh
rubin_rir
rubin_os
rubin_l13
rubin_cb2
rubin_cb1
rubin_RJF
ugc_586_1
ugc_586_10
ugc_586_11
ugc_586_12
ugc_586_18
ugc_586_19
ugc_586_2
ugc_586_3
ugc_586_4
ugc_586_5
ugc_586_6
ugc_586_7
ugc_586_8
ugc_586_9
ugc_586_13
ugc_586_14
ugc_586_15
ugc_586_22
ugc_586_23
ugc_586_25
ugc_586_17
ugc_586_20
ugc_586_21
ugc_610
East
North
West
RJF
21.5
19.1
16.7
14.3
11.9
9.55
7.16
4.78
2.39
1.2e–05

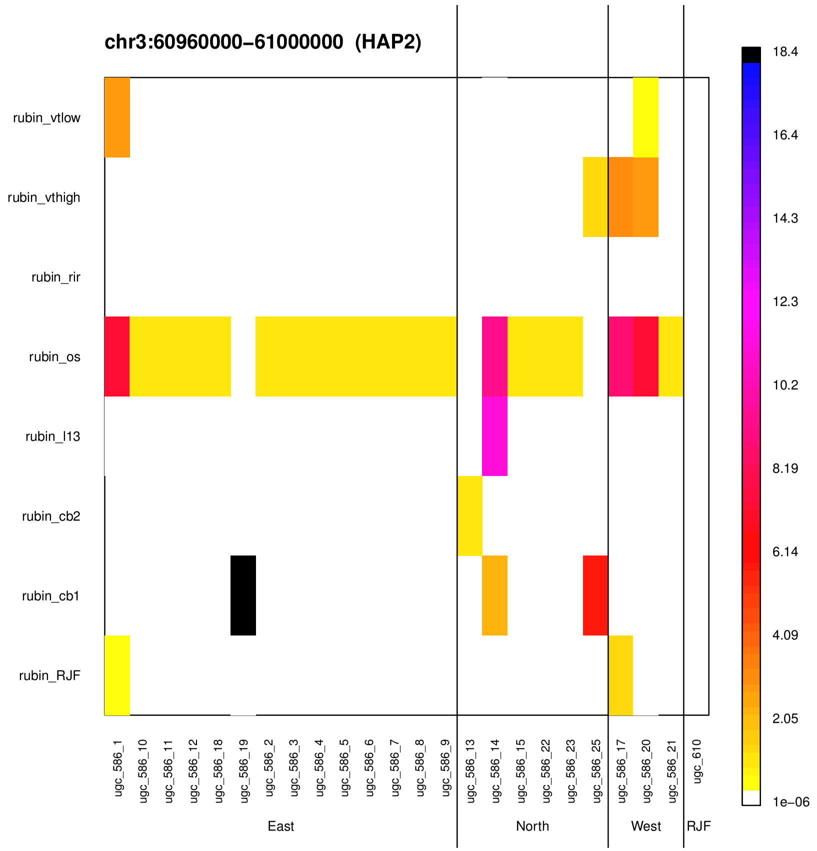
chr3:60960000–61000000 (HAP2)
rubin_vtlow
rubin_vthigh
rubin_rir
rubin_os
rubin_l13
rubin_cb2
rubin_cb1
rubin_RJF
ugc_586_1
ugc_586_10
ugc_586_11
ugc_586_12
ugc_586_18
ugc_586_19
ugc_586_2
ugc_586_3
ugc_586_4
ugc_586_5
ugc_586_6
ugc_586_7
ugc_586_8
ugc_586_9
ugc_586_13
ugc_586_14
ugc_586_15
ugc_586_22
ugc_586_23
ugc_586_25
ugc_586_17
ugc_586_20
ugc_586_21
ugc_610
East
North
West
RJF
18.4
16.4
14.3
12.3
10.2
8.19
6.14
4.09
2.05
1e–06

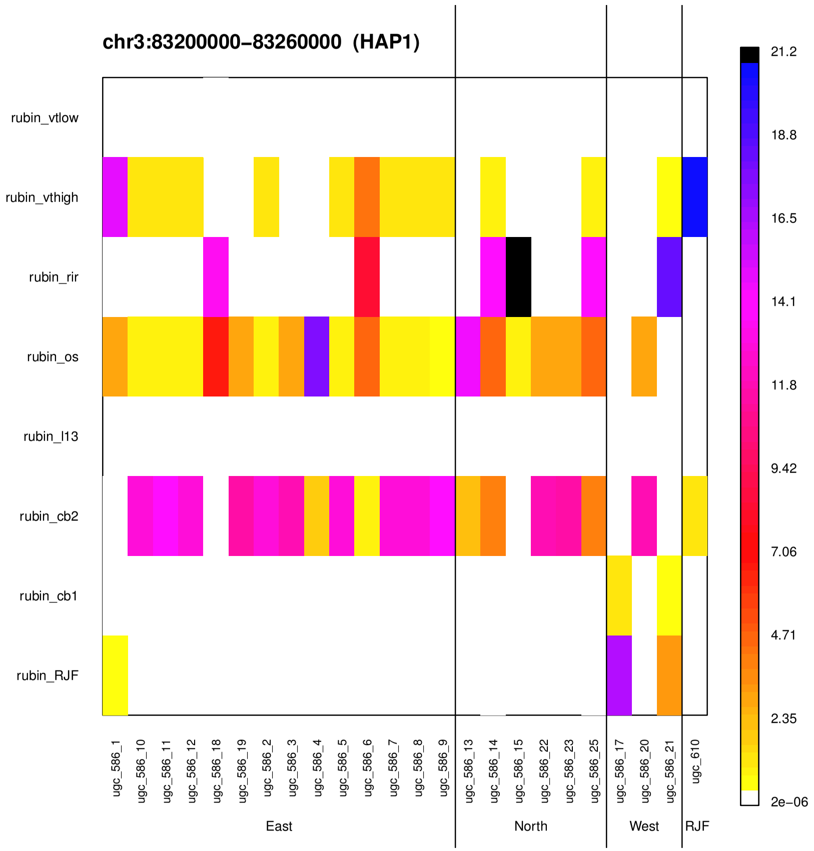
chr3:83200000–83260000 (HAP1)
rubin_vtlow
rubin_vthigh
rubin_rir
rubin_os
rubin_l13
rubin_cb2
rubin_cb1
rubin_RJF
ugc_586_1
ugc_586_10
ugc_586_11
ugc_586_12
ugc_586_18
ugc_586_19
ugc_586_2
ugc_586_3
ugc_586_4
ugc_586_5
ugc_586_6
ugc_586_7
ugc_586_8
ugc_586_9
ugc_586_13
ugc_586_14
ugc_586_15
ugc_586_22
ugc_586_23
ugc_586_25
ugc_586_17
ugc_586_20
ugc_586_21
ugc_610
East
North
West
RJF
21.2
18.8
16.5
14.1
11.8
9.42
7.06
4.71
2.35
2e–06

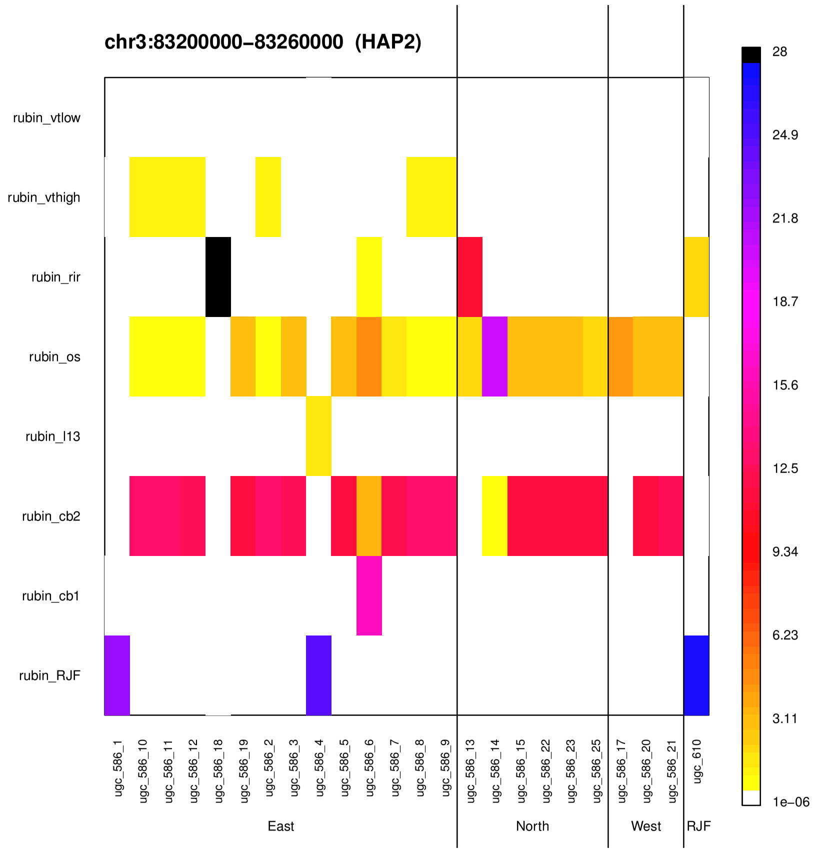
chr3:83200000–83260000 (HAP2)
rubin_vtlow
rubin_vthigh
rubin_rir
rubin_os
rubin_l13
rubin_cb2
rubin_cb1
rubin_RJF
ugc_586_1
ugc_586_10
ugc_586_11
ugc_586_12
ugc_586_18
ugc_586_19
ugc_586_2
ugc_586_3
ugc_586_4
ugc_586_5
ugc_586_6
ugc_586_7
ugc_586_8
ugc_586_9
ugc_586_13
ugc_586_14
ugc_586_15
ugc_586_22
ugc_586_23
ugc_586_25
ugc_586_17
ugc_586_20
ugc_586_21
ugc_610
East
North
West
RJF
28
24.9
21.8
18.7
15.6
12.5
9.34
6.23
3.11
1e–06

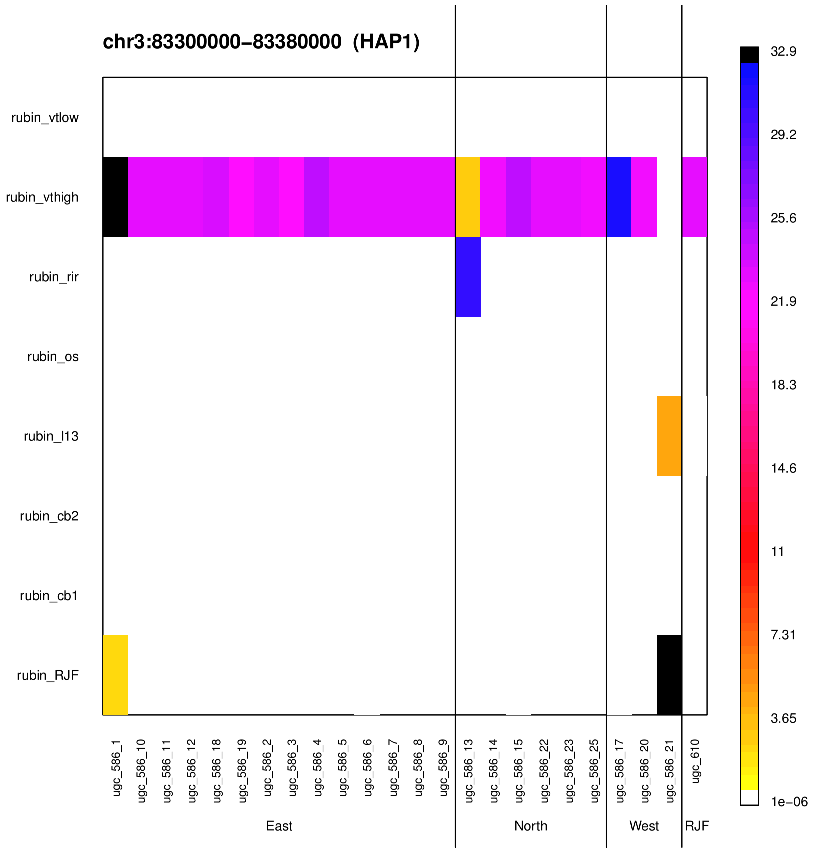
chr3:83300000–83380000 (HAP1)
rubin_vtlow
rubin_vthigh
rubin_rir
rubin_os
rubin_l13
rubin_cb2
rubin_cb1
rubin_RJF
ugc_586_1
ugc_586_10
ugc_586_11
ugc_586_12
ugc_586_18
ugc_586_19
ugc_586_2
ugc_586_3
ugc_586_4
ugc_586_5
ugc_586_6
ugc_586_7
ugc_586_8
ugc_586_9
ugc_586_13
ugc_586_14
ugc_586_15
ugc_586_22
ugc_586_23
ugc_586_25
ugc_586_17
ugc_586_20
ugc_586_21
ugc_610
East
North
West
RJF
32.9
29.2
25.6
21.9
18.3
14.6
11
7.31
3.65
1e–06

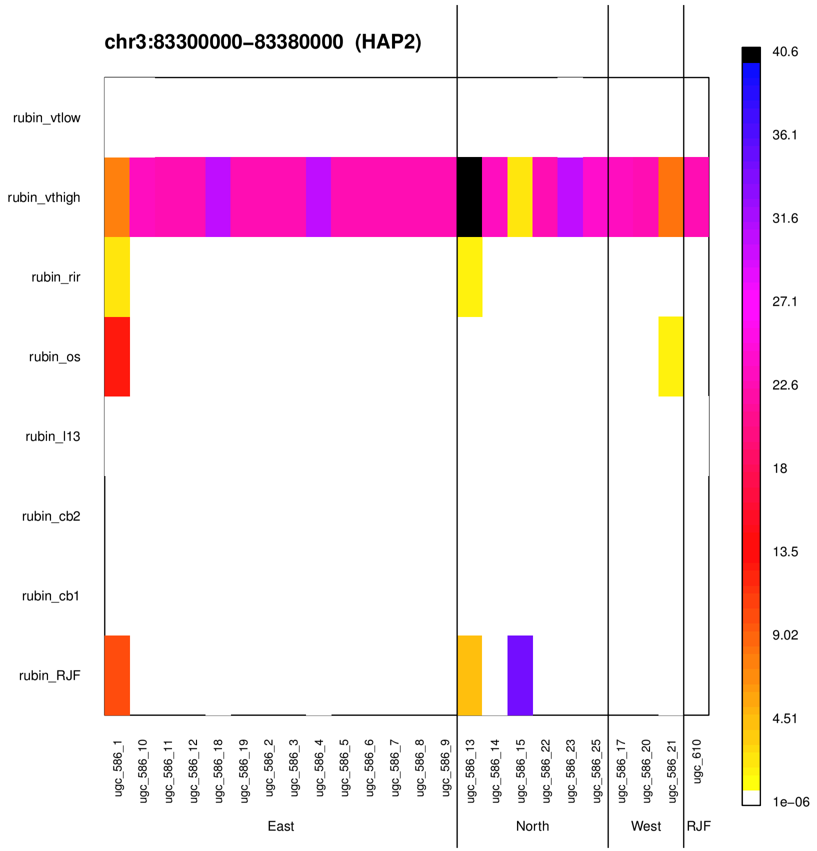
chr3:83300000–83380000 (HAP2)
rubin_vtlow
rubin_vthigh
rubin_rir
rubin_os
rubin_l13
rubin_cb2
rubin_cb1
rubin_RJF
ugc_586_1
ugc_586_10
ugc_586_11
ugc_586_12
ugc_586_18
ugc_586_19
ugc_586_2
ugc_586_3
ugc_586_4
ugc_586_5
ugc_586_6
ugc_586_7
ugc_586_8
ugc_586_9
ugc_586_13
ugc_586_14
ugc_586_15
ugc_586_22
ugc_586_23
ugc_586_25
ugc_586_17
ugc_586_20
ugc_586_21
ugc_610
East
North
West
RJF
40.6
36.1
31.6
27.1
22.6
18
13.5
9.02
4.51
1e–06

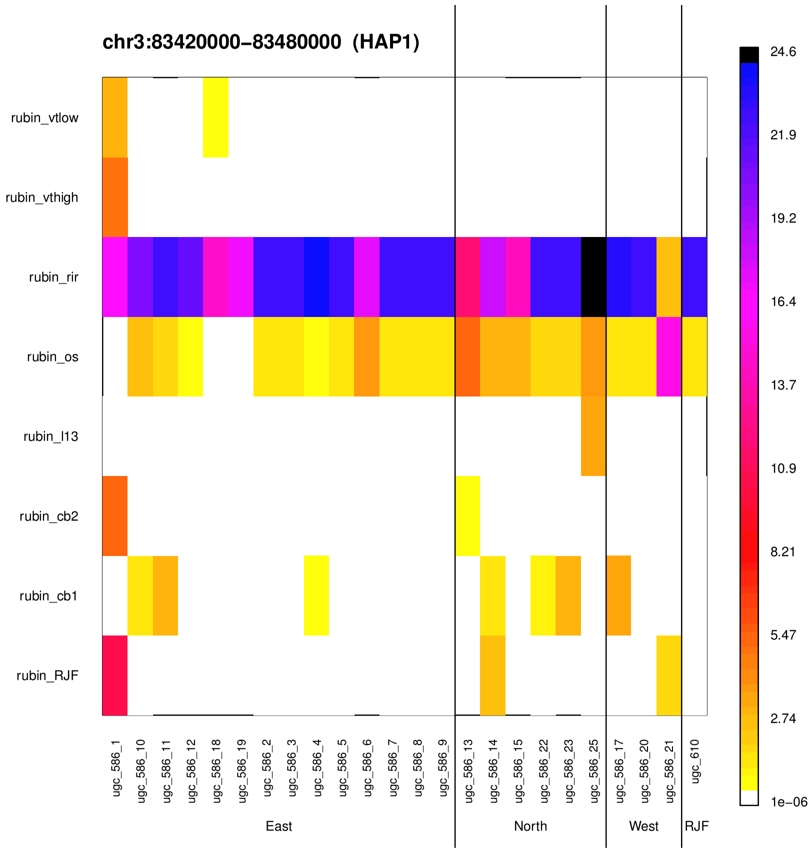
chr3:83420000–83480000 (HAP1)
rubin_vtlow
rubin_vthigh
rubin_rir
rubin_os
rubin_l13
rubin_cb2
rubin_cb1
rubin_RJF
ugc_586_1
ugc_586_10
ugc_586_11
ugc_586_12
ugc_586_18
ugc_586_19
ugc_586_2
ugc_586_3
ugc_586_4
ugc_586_5
ugc_586_6
ugc_586_7
ugc_586_8
ugc_586_9
ugc_586_13
ugc_586_14
ugc_586_15
ugc_586_22
ugc_586_23
ugc_586_25
ugc_586_17
ugc_586_20
ugc_586_21
ugc_610
East
North
West
RJF
24.6
21.9
19.2
16.4
13.7
10.9
8.21
5.47
2.74
1e−06

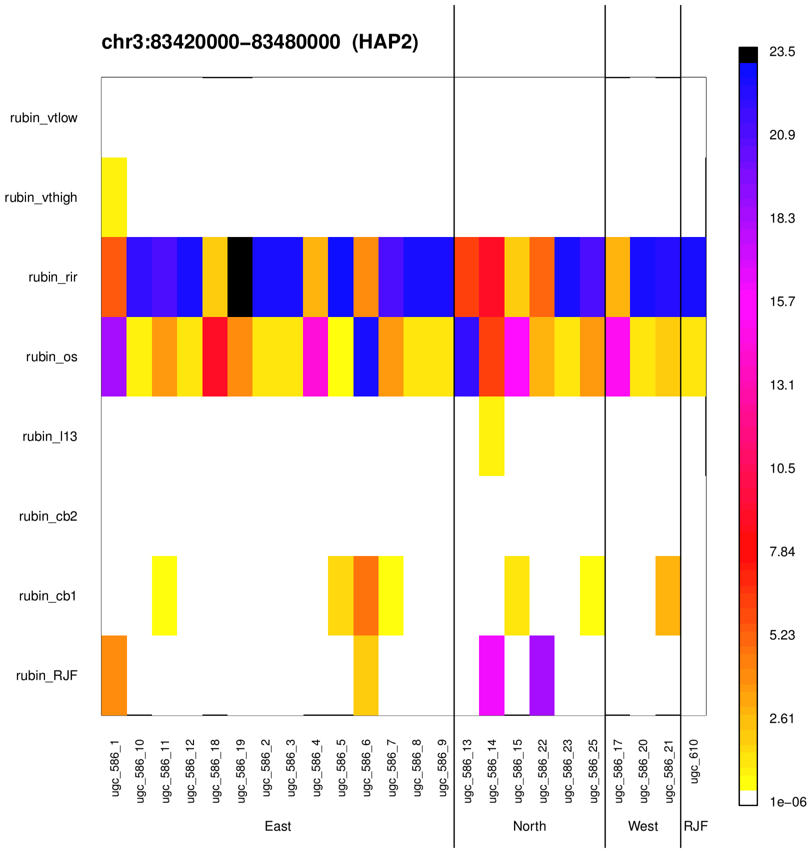
chr3:83420000–83480000 (HAP2)
rubin_vtlow
rubin_vthigh
rubin_rir
rubin_os
rubin_l13
rubin_cb2
rubin_cb1
rubin_RJF
ugc_586_1
ugc_586_10
ugc_586_11
ugc_586_12
ugc_586_18
ugc_586_19
ugc_586_2
ugc_586_3
ugc_586_4
ugc_586_5
ugc_586_6
ugc_586_7
ugc_586_8
ugc_586_9
ugc_586_13
ugc_586_14
ugc_586_15
ugc_586_22
ugc_586_23
ugc_586_25
ugc_586_17
ugc_586_20
ugc_586_21
ugc_610
East
North
West
RJF
23.5
20.9
18.3
15.7
13.1
10.5
7.84
5.23
2.61
1e−06

chr4:9140000–9180000 (HAP1)

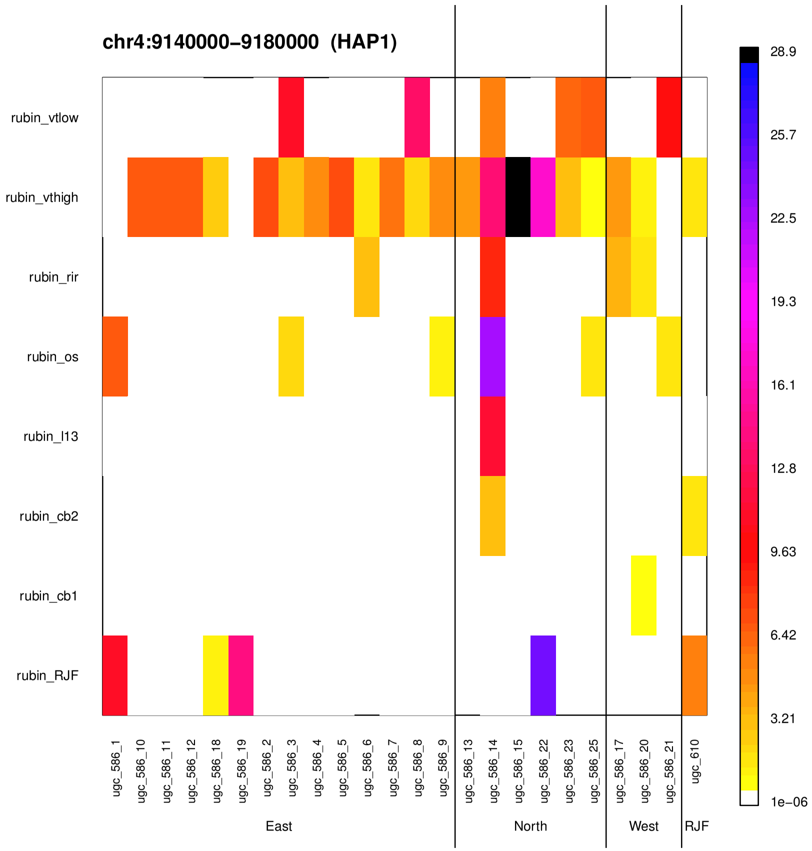

chr4:9140000–9180000 (HAP2)

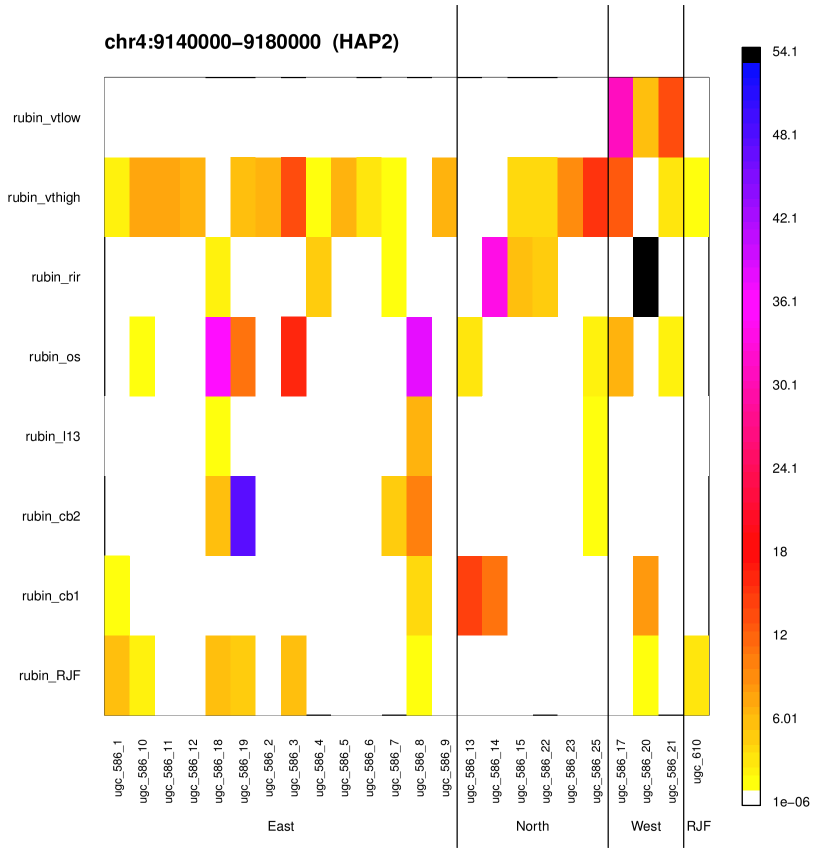

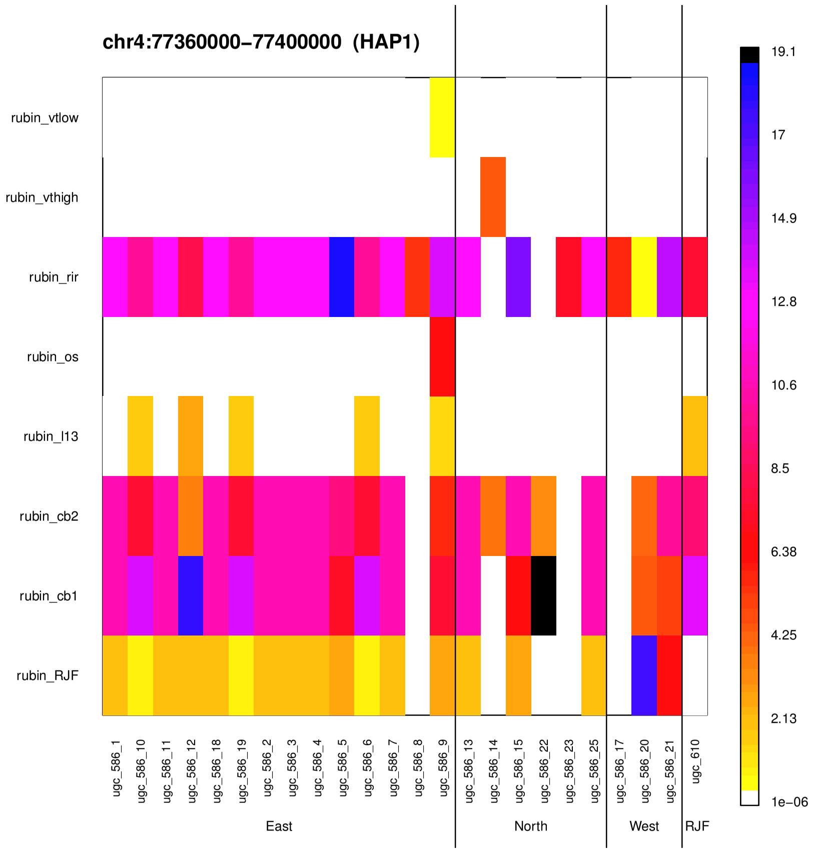
chr4:77360000-77400000 (HAP1)
rubin_vtlow
rubin_vthigh
rubin_rir
rubin_os
rubin_l13
rubin_cb2
rubin_cb1
rubin_RJF
ugc_586_1
ugc_586_10
ugc_586_11
ugc_586_12
ugc_586_18
ugc_586_19
ugc_586_2
ugc_586_3
ugc_586_4
ugc_586_5
ugc_586_6
ugc_586_7
ugc_586_8
ugc_586_9
ugc_586_13
ugc_586_14
ugc_586_15
ugc_586_22
ugc_586_23
ugc_586_25
ugc_586_17
ugc_586_20
ugc_586_21
ugc_610
East
North
West
RJF
19.1
17
14.9
12.8
10.6
8.5
6.38
4.25
2.13
1e−06

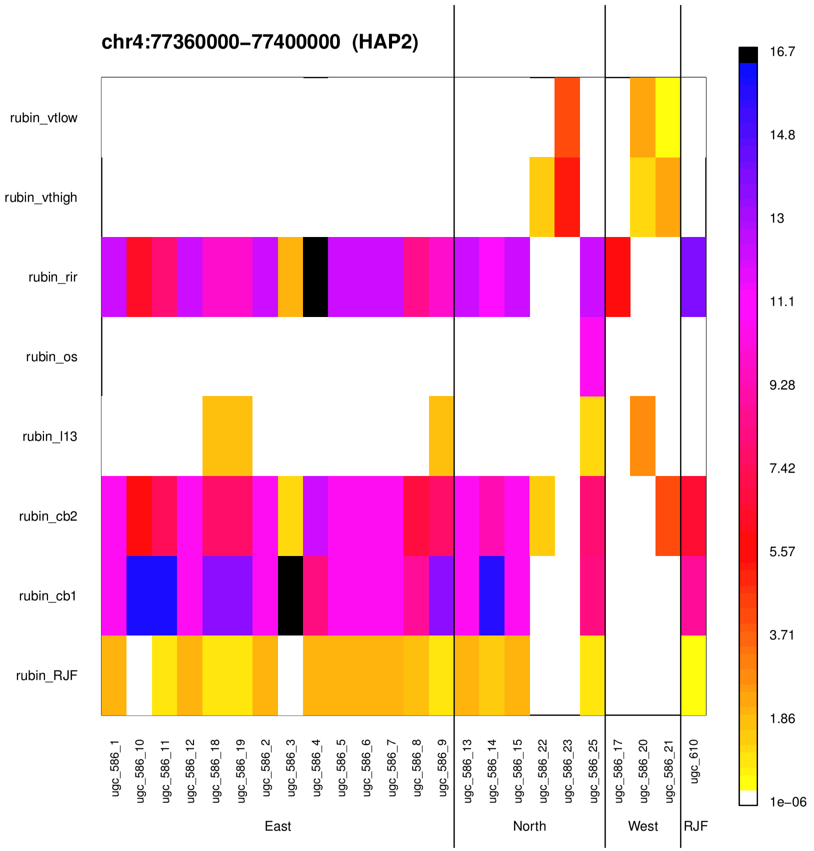
chr4:77360000-77400000 (HAP2)
rubin_vtlow
rubin_vthigh
rubin_rir
rubin_os
rubin_l13
rubin_cb2
rubin_cb1
rubin_RJF
ugc_586_1
ugc_586_10
ugc_586_11
ugc_586_12
ugc_586_18
ugc_586_19
ugc_586_2
ugc_586_3
ugc_586_4
ugc_586_5
ugc_586_6
ugc_586_7
ugc_586_8
ugc_586_9
ugc_586_13
ugc_586_14
ugc_586_15
ugc_586_22
ugc_586_23
ugc_586_25
ugc_586_17
ugc_586_20
ugc_586_21
ugc_610
East
North
West
RJF
16.7
14.8
13
11.1
9.28
7.42
5.57
3.71
1.86
1e−06

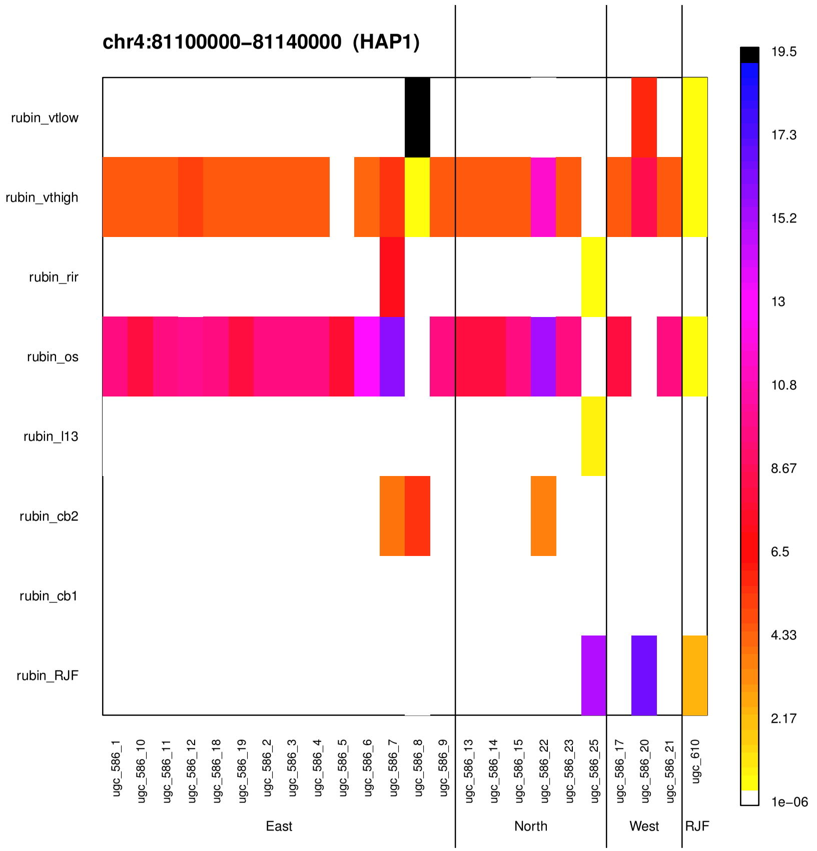
chr4:81100000–81140000 (HAP1)
rubin_vtlow
rubin_vthigh
rubin_rir
rubin_os
rubin_l13
rubin_cb2
rubin_cb1
rubin_RJF
ugc_586_1
ugc_586_10
ugc_586_11
ugc_586_12
ugc_586_18
ugc_586_19
ugc_586_2
ugc_586_3
ugc_586_4
ugc_586_5
ugc_586_6
ugc_586_7
ugc_586_8
ugc_586_9
ugc_586_13
ugc_586_14
ugc_586_15
ugc_586_22
ugc_586_23
ugc_586_25
ugc_586_17
ugc_586_20
ugc_586_21
ugc_610
East
North
West
RJF
19.5
17.3
15.2
13
10.8
8.67
6.5
4.33
2.17
1e−06

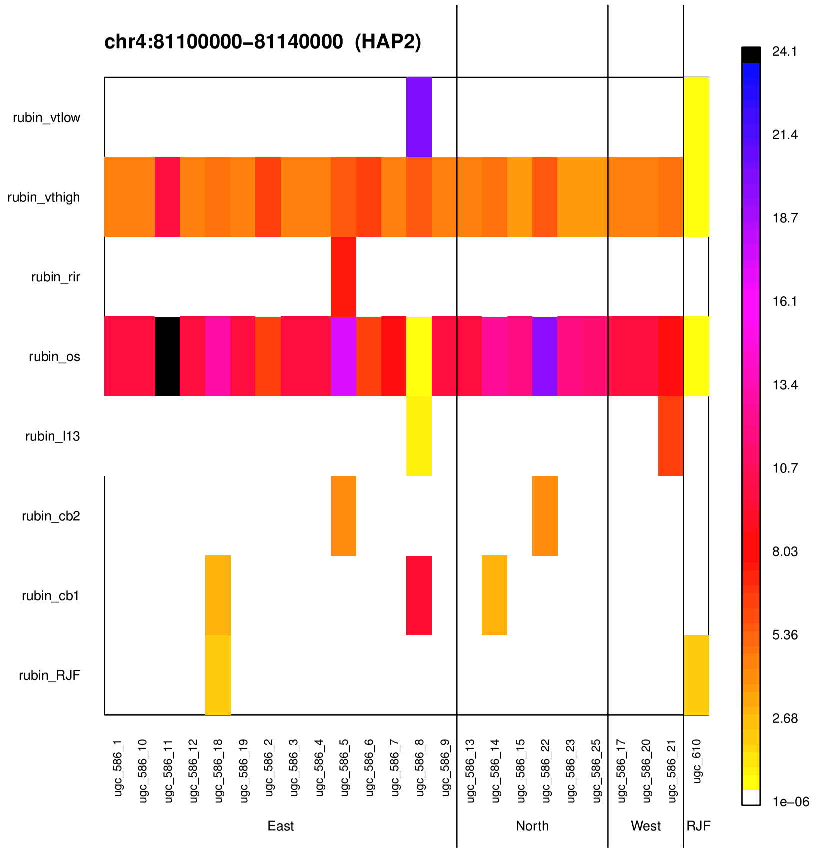
chr4:81100000–81140000 (HAP2)
rubin_vtlow
rubin_vthigh
rubin_rir
rubin_os
rubin_l13
rubin_cb2
rubin_cb1
rubin_RJF
ugc_586_1
ugc_586_10
ugc_586_11
ugc_586_12
ugc_586_18
ugc_586_19
ugc_586_2
ugc_586_3
ugc_586_4
ugc_586_5
ugc_586_6
ugc_586_7
ugc_586_8
ugc_586_9
ugc_586_13
ugc_586_14
ugc_586_15
ugc_586_22
ugc_586_23
ugc_586_25
ugc_586_17
ugc_586_20
ugc_586_21
ugc_610
East
North
West
RJF
24.1
21.4
18.7
16.1
13.4
10.7
8.03
5.36
2.68
1e−06

chr5:3840000–3880000 (HAP1)

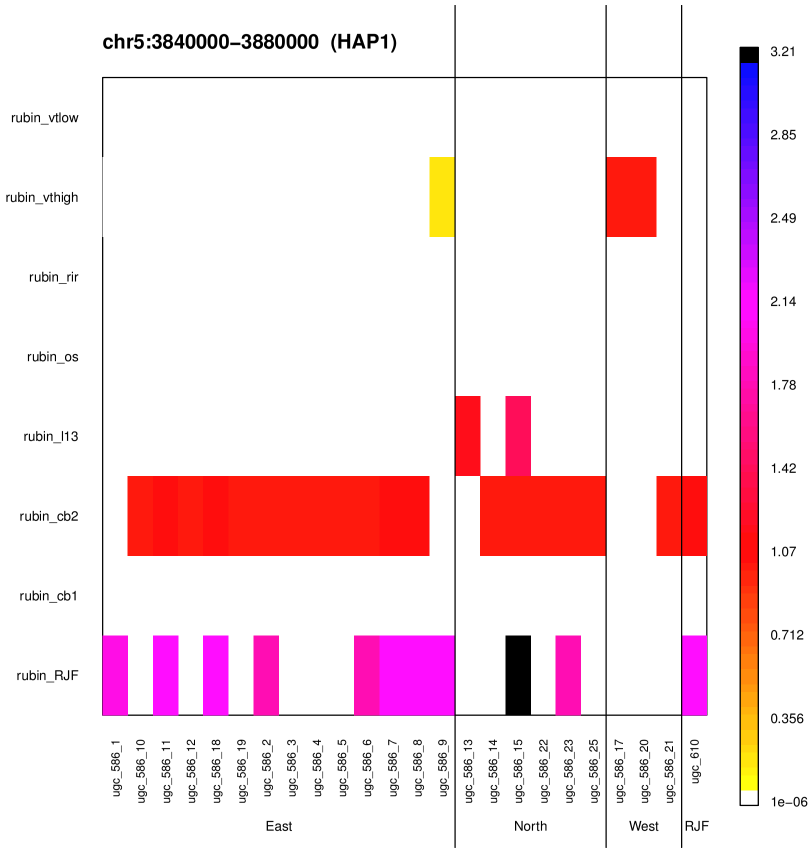

chr5:3840000–3880000 (HAP2)

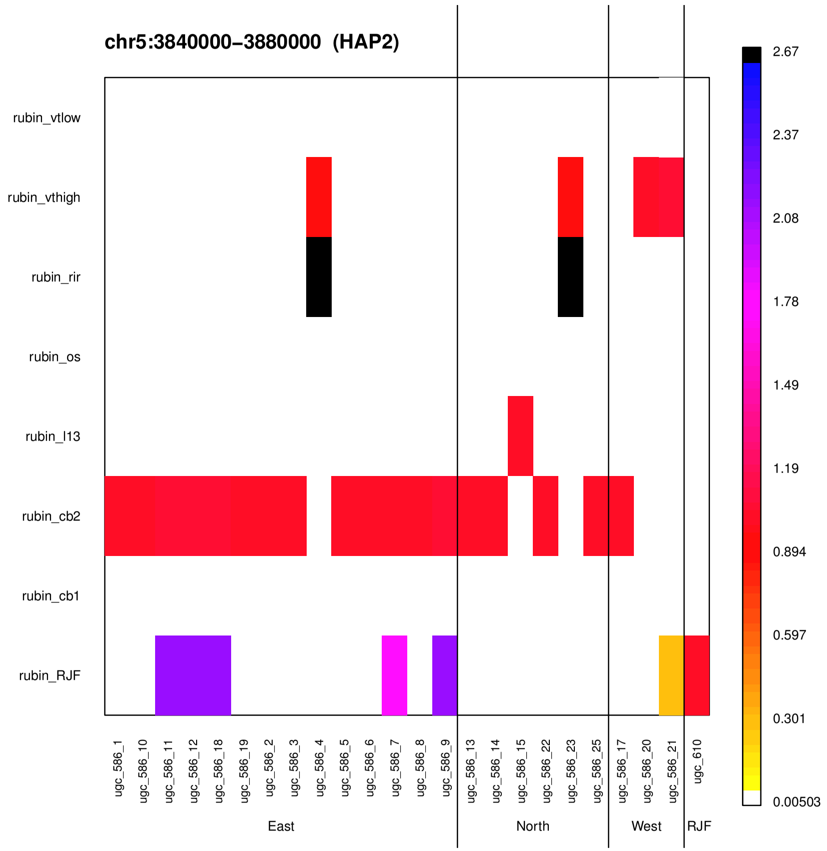

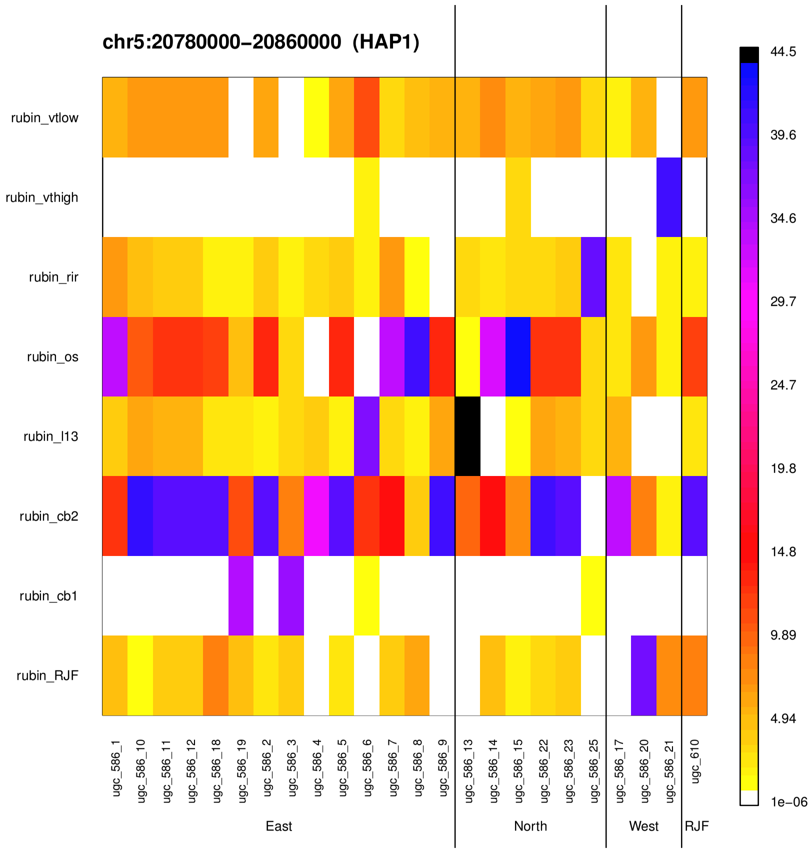
chr5:20780000-20860000 (HAP1)
rubin_vtlow
rubin_vthigh
rubin_rir
rubin_os
rubin_l13
rubin_cb2
rubin_cb1
rubin_RJF
ugc_586_1
ugc_586_10
ugc_586_11
ugc_586_12
ugc_586_18
ugc_586_19
ugc_586_2
ugc_586_3
ugc_586_4
ugc_586_5
ugc_586_6
ugc_586_7
ugc_586_8
ugc_586_9
ugc_586_13
ugc_586_14
ugc_586_15
ugc_586_22
ugc_586_23
ugc_586_25
ugc_586_17
ugc_586_20
ugc_586_21
ugc_610
East
North
West
RJF
44.5
39.6
34.6
29.7
24.7
19.8
14.8
9.89
4.94
1e−06

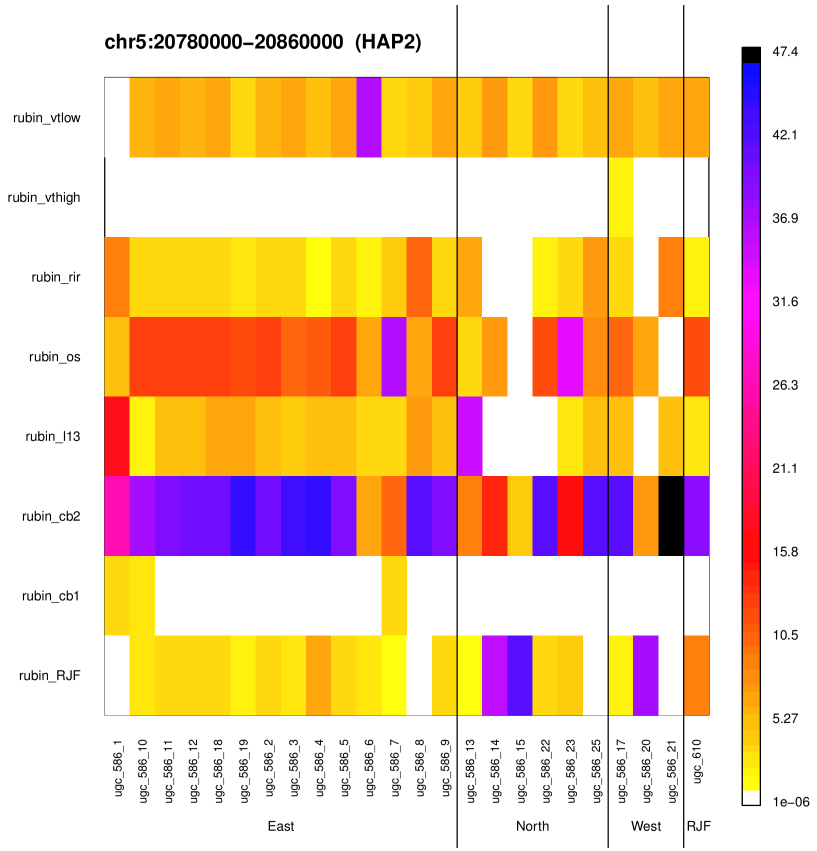
chr5:20780000-20860000 (HAP2)
rubin_vtlow
rubin_vthigh
rubin_rir
rubin_os
rubin_l13
rubin_cb2
rubin_cb1
rubin_RJF
ugc_586_1
ugc_586_10
ugc_586_11
ugc_586_12
ugc_586_18
ugc_586_19
ugc_586_2
ugc_586_3
ugc_586_4
ugc_586_5
ugc_586_6
ugc_586_7
ugc_586_8
ugc_586_9
ugc_586_13
ugc_586_14
ugc_586_15
ugc_586_22
ugc_586_23
ugc_586_25
ugc_586_17
ugc_586_20
ugc_586_21
ugc_610
East
North
West
RJF
47.4
42.1
36.9
31.6
26.3
21.1
15.8
10.5
5.27
1e−06

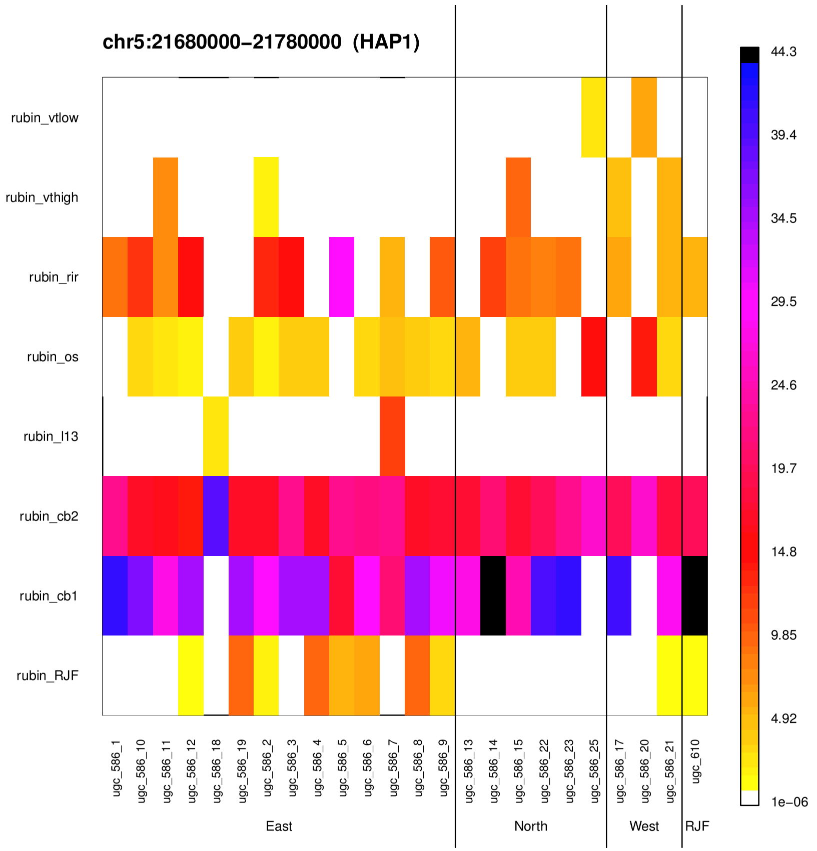
chr5:21680000-21780000 (HAP1)
rubin_vtlow
rubin_vthigh
rubin_rir
rubin_os
rubin_l13
rubin_cb2
rubin_cb1
rubin_RJF
ugc_586_1
ugc_586_10
ugc_586_11
ugc_586_12
ugc_586_18
ugc_586_19
ugc_586_2
ugc_586_3
ugc_586_4
ugc_586_5
ugc_586_6
ugc_586_7
ugc_586_8
ugc_586_9
ugc_586_13
ugc_586_14
ugc_586_15
ugc_586_22
ugc_586_23
ugc_586_25
ugc_586_17
ugc_586_20
ugc_586_21
ugc_610
East
North
West
RJF
44.3
39.4
34.5
29.5
24.6
19.7
14.8
9.85
4.92
1e-06

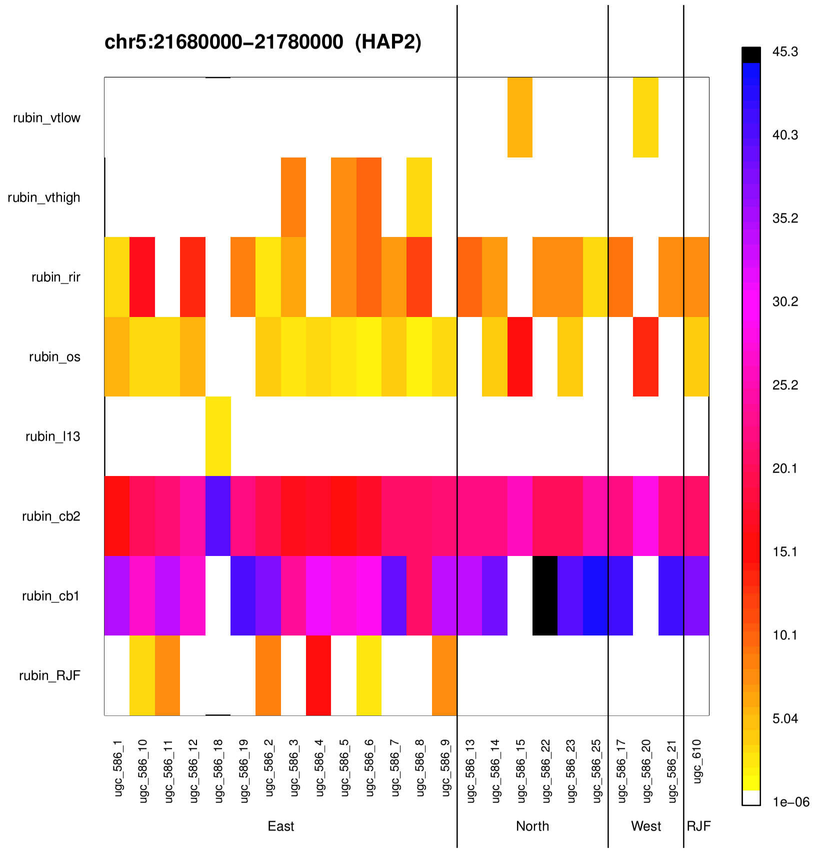
chr5:21680000-21780000 (HAP2)
rubin_vtlow
rubin_vthigh
rubin_rir
rubin_os
rubin_l13
rubin_cb2
rubin_cb1
rubin_RJF
ugc_586_1
ugc_586_10
ugc_586_11
ugc_586_12
ugc_586_18
ugc_586_19
ugc_586_2
ugc_586_3
ugc_586_4
ugc_586_5
ugc_586_6
ugc_586_7
ugc_586_8
ugc_586_9
ugc_586_13
ugc_586_14
ugc_586_15
ugc_586_22
ugc_586_23
ugc_586_25
ugc_586_17
ugc_586_20
ugc_586_21
ugc_610
East
North
West
RJF
45.3
40.3
35.2
30.2
25.2
20.1
15.1
10.1
5.04
1e-06

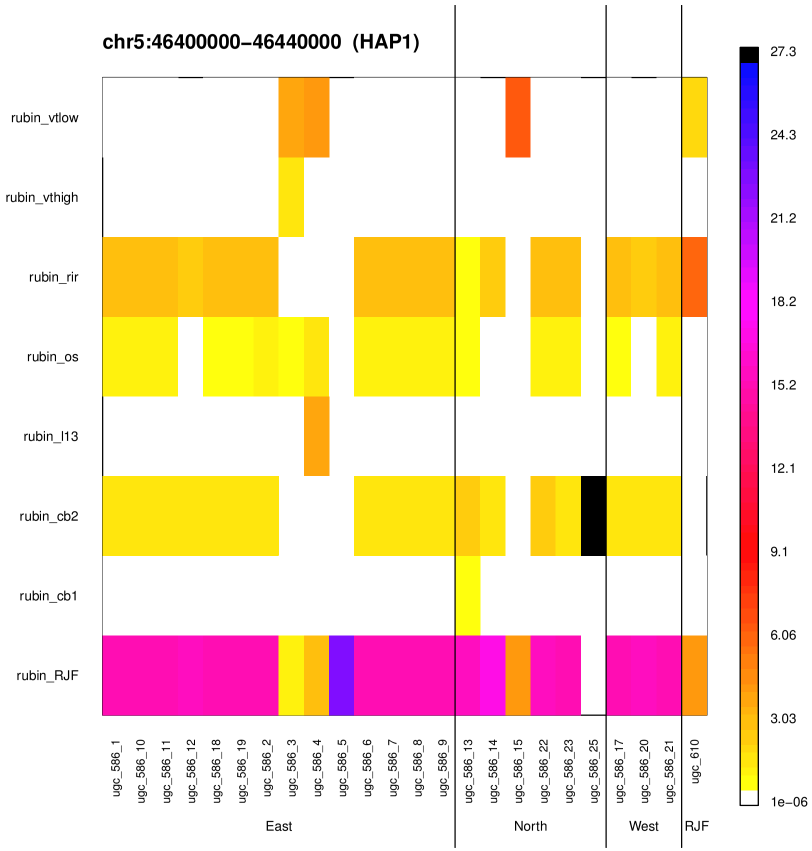
chr5:46400000–46440000 (HAP1)
rubin_vtlow
rubin_vthigh
rubin_rir
rubin_os
rubin_l13
rubin_cb2
rubin_cb1
rubin_RJF
ugc_586_1
ugc_586_10
ugc_586_11
ugc_586_12
ugc_586_18
ugc_586_19
ugc_586_2
ugc_586_3
ugc_586_4
ugc_586_5
ugc_586_6
ugc_586_7
ugc_586_8
ugc_586_9
ugc_586_13
ugc_586_14
ugc_586_15
ugc_586_22
ugc_586_23
ugc_586_25
ugc_586_17
ugc_586_20
ugc_586_21
ugc_610
East
North
West
RJF
27.3
24.3
21.2
18.2
15.2
12.1
9.1
6.06
3.03
1e–06

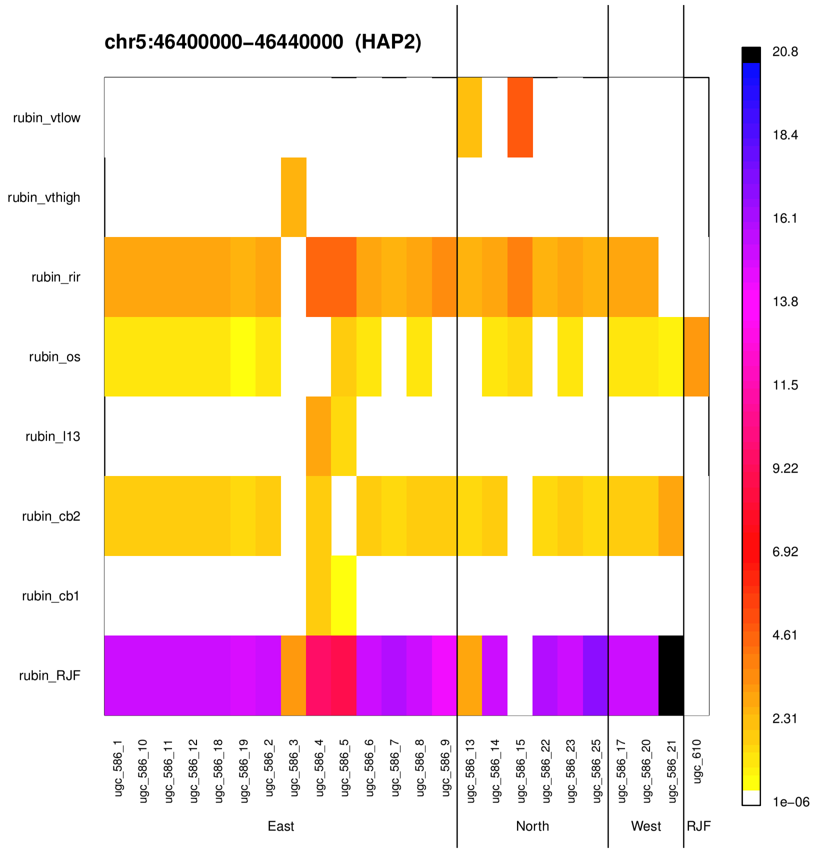
chr5:46400000–46440000 (HAP2)
rubin_vtlow
rubin_vthigh
rubin_rir
rubin_os
rubin_l13
rubin_cb2
rubin_cb1
rubin_RJF
ugc_586_1
ugc_586_10
ugc_586_11
ugc_586_12
ugc_586_18
ugc_586_19
ugc_586_2
ugc_586_3
ugc_586_4
ugc_586_5
ugc_586_6
ugc_586_7
ugc_586_8
ugc_586_9
ugc_586_13
ugc_586_14
ugc_586_15
ugc_586_22
ugc_586_23
ugc_586_25
ugc_586_17
ugc_586_20
ugc_586_21
ugc_610
East
North
West
RJF
20.8
18.4
16.1
13.8
11.5
9.22
6.92
4.61
2.31
1e–06

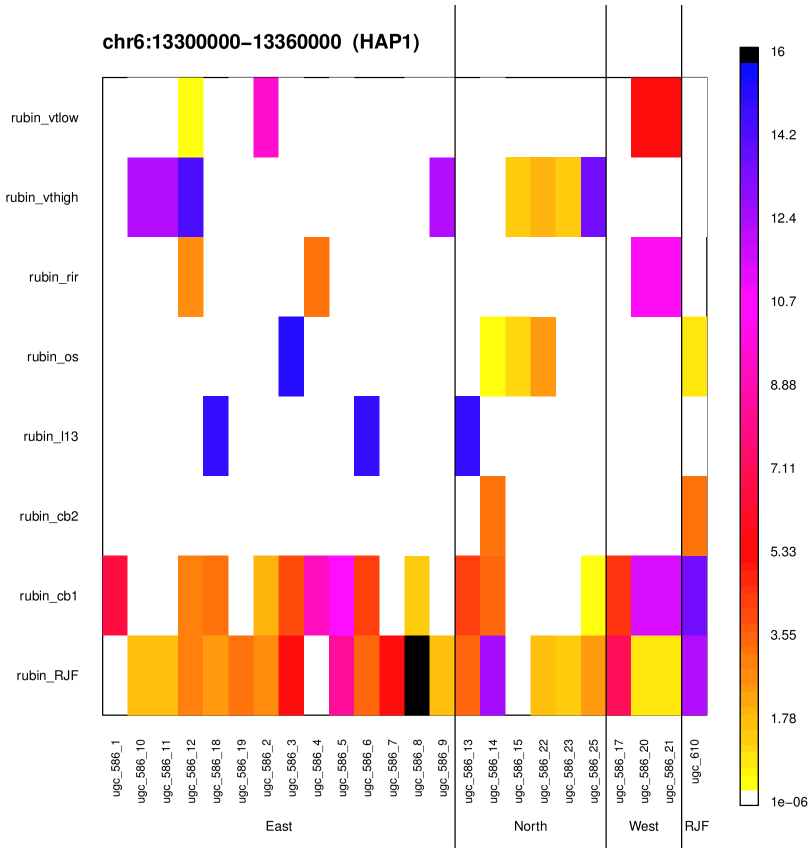
chr6:13300000–13360000 (HAP1)
rubin_vtlow
rubin_vthigh
rubin_rir
rubin_os
rubin_l13
rubin_cb2
rubin_cb1
rubin_RJF
ugc_586_1
ugc_586_10
ugc_586_11
ugc_586_12
ugc_586_18
ugc_586_19
ugc_586_2
ugc_586_3
ugc_586_4
ugc_586_5
ugc_586_6
ugc_586_7
ugc_586_8
ugc_586_9
ugc_586_13
ugc_586_14
ugc_586_15
ugc_586_22
ugc_586_23
ugc_586_25
ugc_586_17
ugc_586_20
ugc_586_21
ugc_610
East
North
West
RJF
16
14.2
12.4
10.7
8.88
7.11
5.33
3.55
1.78
1e−06

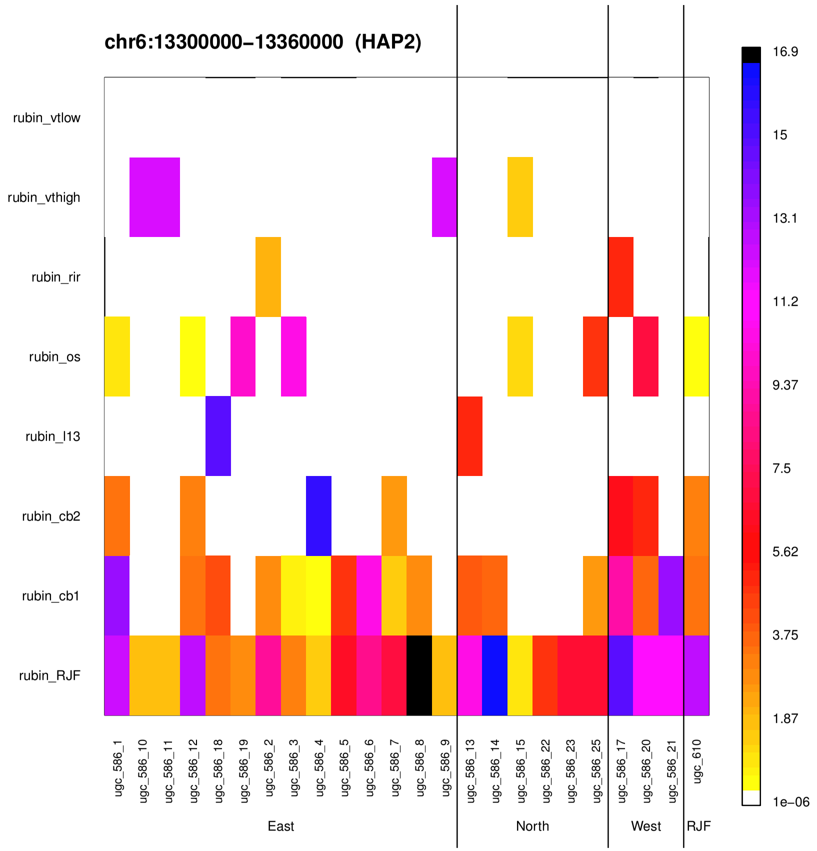
chr6:13300000–13360000 (HAP2)
rubin_vtlow
rubin_vthigh
rubin_rir
rubin_os
rubin_l13
rubin_cb2
rubin_cb1
rubin_RJF
ugc_586_1
ugc_586_10
ugc_586_11
ugc_586_12
ugc_586_18
ugc_586_19
ugc_586_2
ugc_586_3
ugc_586_4
ugc_586_5
ugc_586_6
ugc_586_7
ugc_586_8
ugc_586_9
ugc_586_13
ugc_586_14
ugc_586_15
ugc_586_22
ugc_586_23
ugc_586_25
ugc_586_17
ugc_586_20
ugc_586_21
ugc_610
East
North
West
RJF
16.9
15
13.1
11.2
9.37
7.5
5.62
3.75
1.87
1e−06

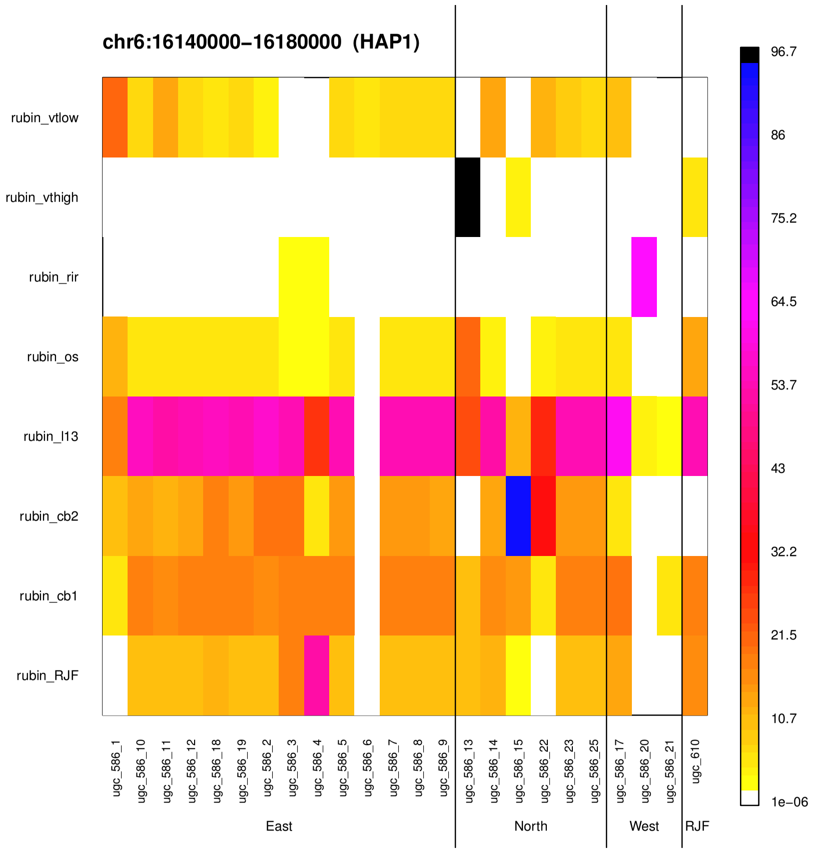
chr6:16140000–16180000 (HAP1)
rubin_vtlow
rubin_vthigh
rubin_rir
rubin_os
rubin_l13
rubin_cb2
rubin_cb1
rubin_RJF
ugc_586_1
ugc_586_10
ugc_586_11
ugc_586_12
ugc_586_18
ugc_586_19
ugc_586_2
ugc_586_3
ugc_586_4
ugc_586_5
ugc_586_6
ugc_586_7
ugc_586_8
ugc_586_9
ugc_586_13
ugc_586_14
ugc_586_15
ugc_586_22
ugc_586_23
ugc_586_25
ugc_586_17
ugc_586_20
ugc_586_21
ugc_610
East
North
West
RJF
96.7
86
75.2
64.5
53.7
43
32.2
21.5
10.7
1e−06

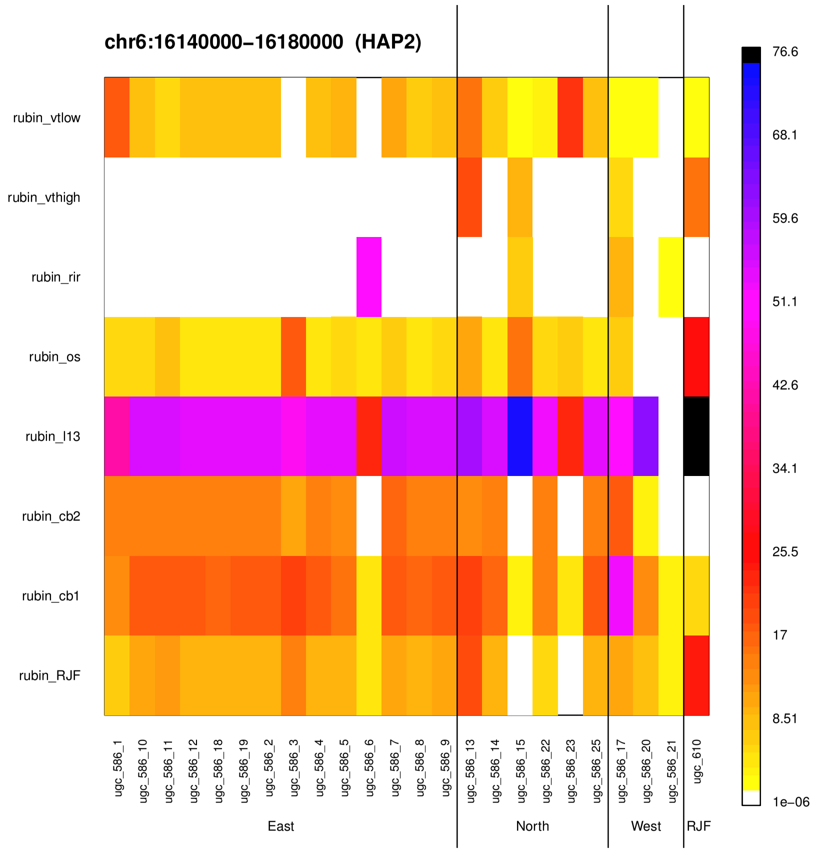
chr6:16140000–16180000 (HAP2)
rubin_vtlow
rubin_vthigh
rubin_rir
rubin_os
rubin_l13
rubin_cb2
rubin_cb1
rubin_RJF
ugc_586_1
ugc_586_10
ugc_586_11
ugc_586_12
ugc_586_18
ugc_586_19
ugc_586_2
ugc_586_3
ugc_586_4
ugc_586_5
ugc_586_6
ugc_586_7
ugc_586_8
ugc_586_9
ugc_586_13
ugc_586_14
ugc_586_15
ugc_586_22
ugc_586_23
ugc_586_25
ugc_586_17
ugc_586_20
ugc_586_21
ugc_610
East
North
West
RJF
76.6
68.1
59.6
51.1
42.6
34.1
25.5
17
8.51
1e−06

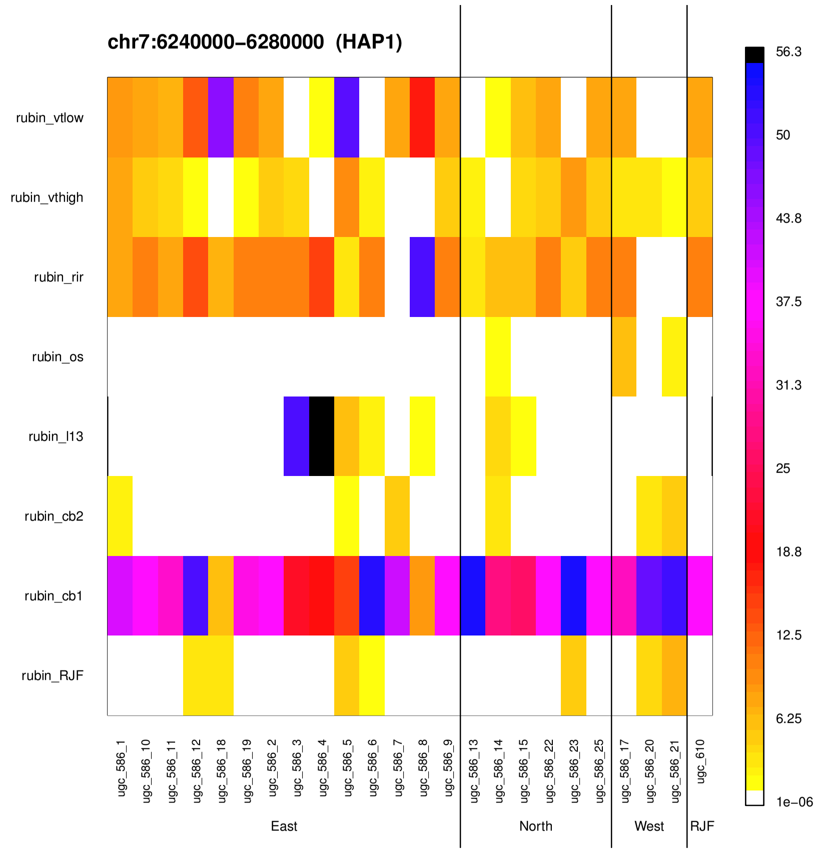
chr7:6240000–6280000 (HAP1)
rubin_vtlow
rubin_vthigh
rubin_rir
rubin_os
rubin_l13
rubin_cb2
rubin_cb1
rubin_RJF
ugc_586_1
ugc_586_10
ugc_586_11
ugc_586_12
ugc_586_18
ugc_586_19
ugc_586_2
ugc_586_3
ugc_586_4
ugc_586_5
ugc_586_6
ugc_586_7
ugc_586_8
ugc_586_9
ugc_586_13
ugc_586_14
ugc_586_15
ugc_586_22
ugc_586_23
ugc_586_25
ugc_586_17
ugc_586_20
ugc_586_21
ugc_610
East
North
West
RJF
56.3
50
43.8
37.5
31.3
25
18.8
12.5
6.25
1e−06

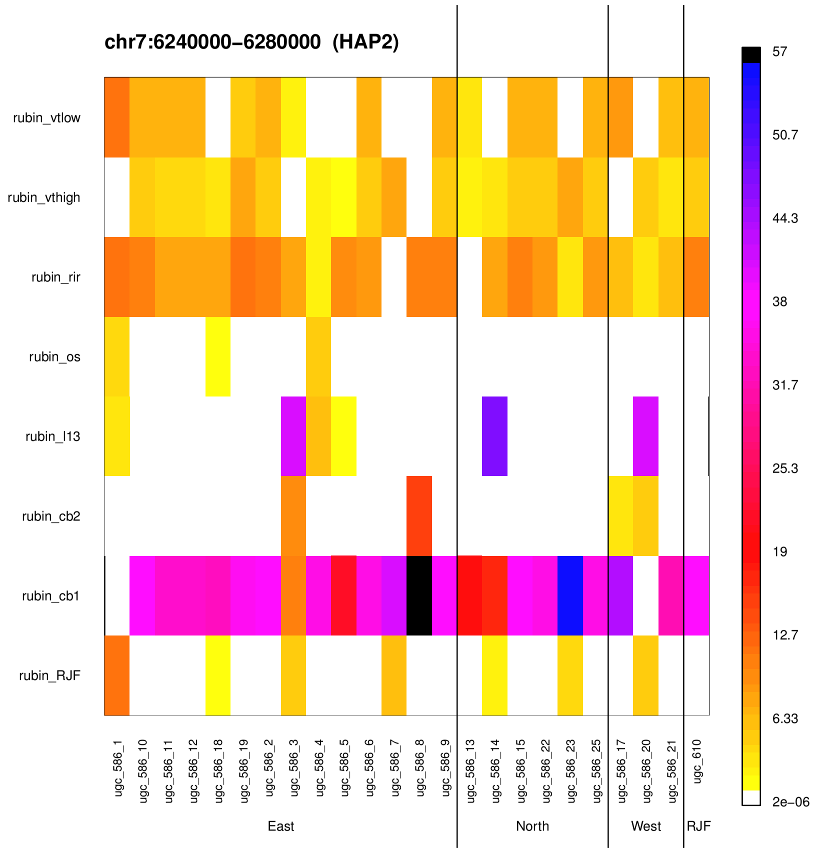
chr7:6240000–6280000 (HAP2)
rubin_vtlow
rubin_vthigh
rubin_rir
rubin_os
rubin_l13
rubin_cb2
rubin_cb1
rubin_RJF
ugc_586_1
ugc_586_10
ugc_586_11
ugc_586_12
ugc_586_18
ugc_586_19
ugc_586_2
ugc_586_3
ugc_586_4
ugc_586_5
ugc_586_6
ugc_586_7
ugc_586_8
ugc_586_9
ugc_586_13
ugc_586_14
ugc_586_15
ugc_586_22
ugc_586_23
ugc_586_25
ugc_586_17
ugc_586_20
ugc_586_21
ugc_610
East
North
West
RJF
57
50.7
44.3
38
31.7
25.3
19
12.7
6.33
2e−06

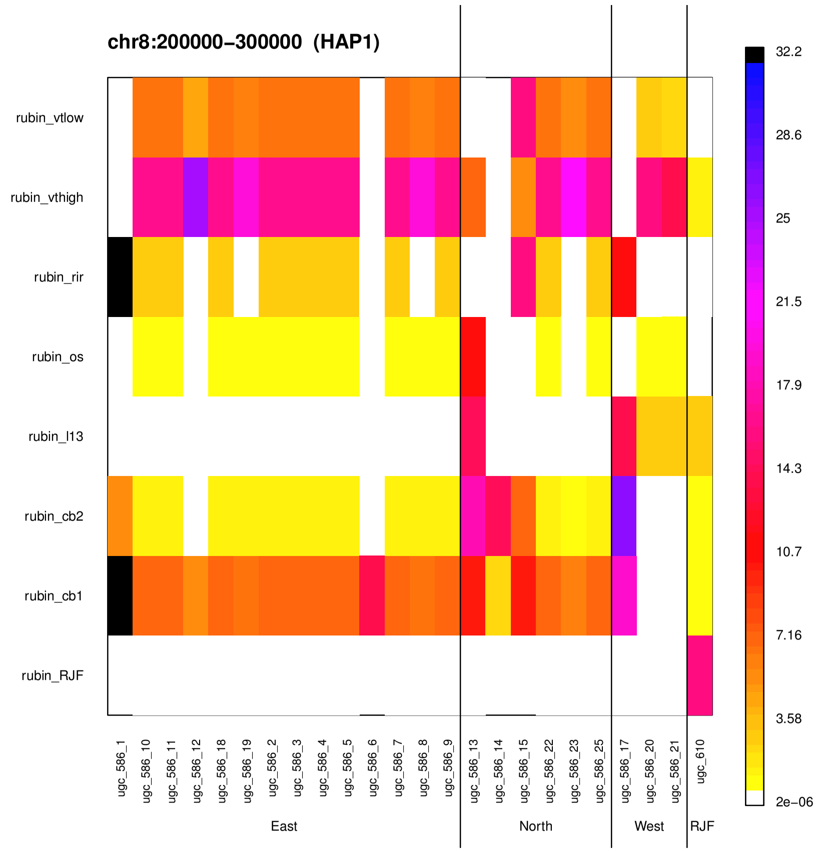
chr8:200000-300000 (HAP1)
rubin_vtlow
rubin_vthigh
rubin_rir
rubin_os
rubin_l13
rubin_cb2
rubin_cb1
rubin_RJF
ugc_586_1
ugc_586_10
ugc_586_11
ugc_586_12
ugc_586_18
ugc_586_19
ugc_586_2
ugc_586_3
ugc_586_4
ugc_586_5
ugc_586_6
ugc_586_7
ugc_586_8
ugc_586_9
ugc_586_13
ugc_586_14
ugc_586_15
ugc_586_22
ugc_586_23
ugc_586_25
ugc_586_17
ugc_586_20
ugc_586_21
ugc_610
East
North
West
RJF
32.2
28.6
25
21.5
17.9
14.3
10.7
7.16
3.58
2e-06

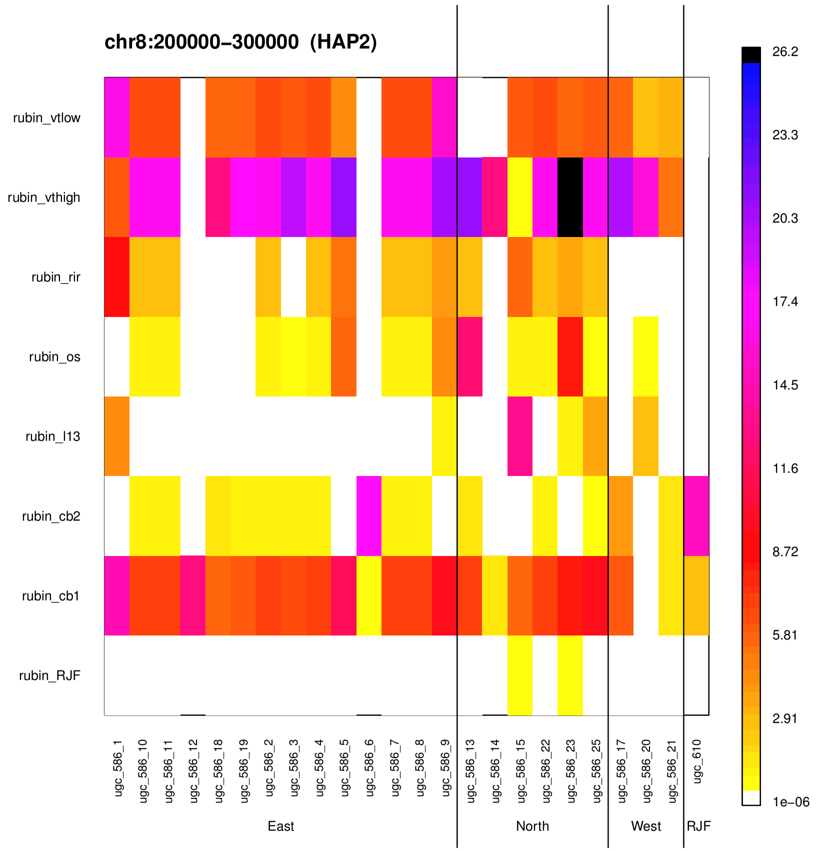
chr8:200000-300000 (HAP2)
rubin_vtlow
rubin_vthigh
rubin_rir
rubin_os
rubin_l13
rubin_cb2
rubin_cb1
rubin_RJF
ugc_586_1
ugc_586_10
ugc_586_11
ugc_586_12
ugc_586_18
ugc_586_19
ugc_586_2
ugc_586_3
ugc_586_4
ugc_586_5
ugc_586_6
ugc_586_7
ugc_586_8
ugc_586_9
ugc_586_13
ugc_586_14
ugc_586_15
ugc_586_22
ugc_586_23
ugc_586_25
ugc_586_17
ugc_586_20
ugc_586_21
ugc_610
East
North
West
RJF
26.2
23.3
20.3
17.4
14.5
11.6
8.72
5.81
2.91
1e-06

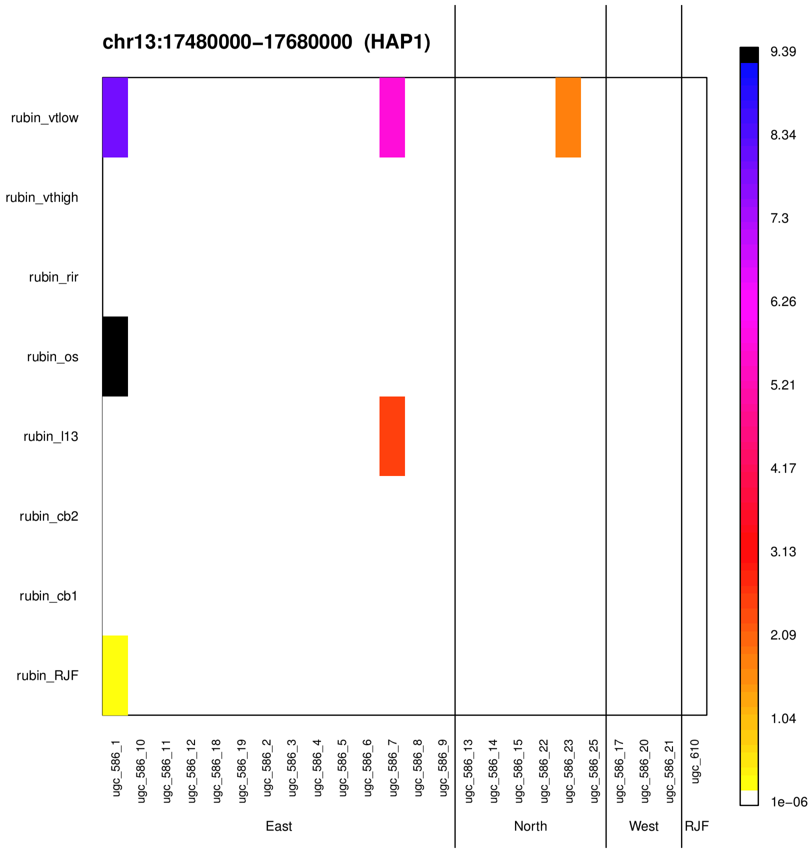
chr13:17480000–17680000 (HAP1)
rubin_vtlow
rubin_vthigh
rubin_rir
rubin_os
rubin_l13
rubin_cb2
rubin_cb1
rubin_RJF
ugc_586_1
ugc_586_10
ugc_586_11
ugc_586_12
ugc_586_18
ugc_586_19
ugc_586_2
ugc_586_3
ugc_586_4
ugc_586_5
ugc_586_6
ugc_586_7
ugc_586_8
ugc_586_9
ugc_586_13
ugc_586_14
ugc_586_15
ugc_586_22
ugc_586_23
ugc_586_25
ugc_586_17
ugc_586_20
ugc_586_21
ugc_610
East
North
West
RJF
9.39
8.34
7.3
6.26
5.21
4.17
3.13
2.09
1.04
1e−06

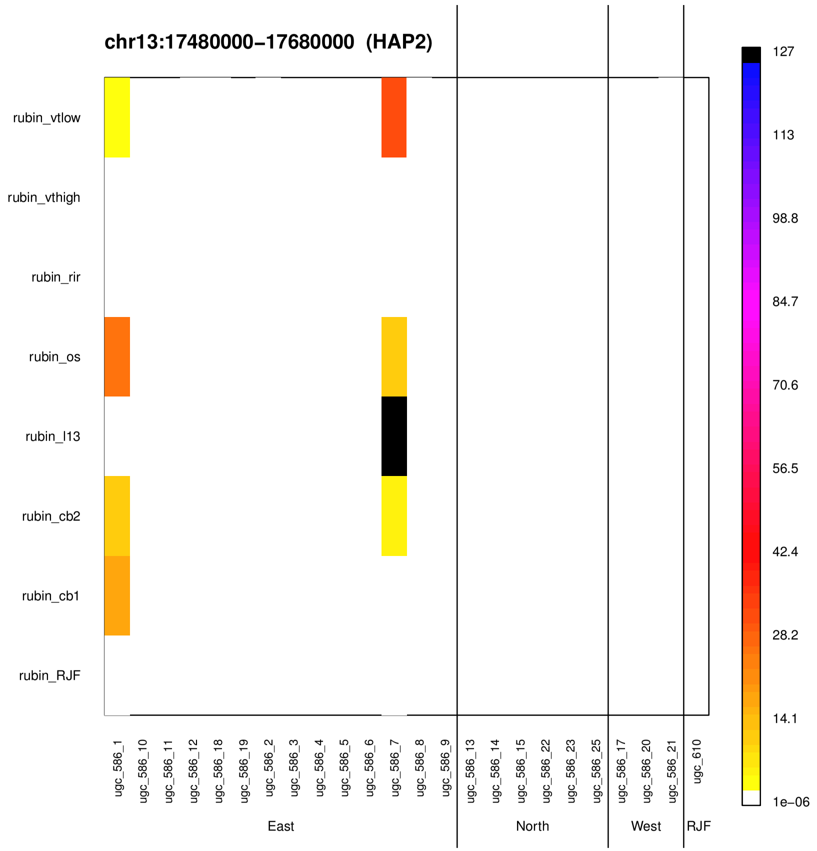
chr13:17480000–17680000 (HAP2)
rubin_vtlow
rubin_vthigh
rubin_rir
rubin_os
rubin_l13
rubin_cb2
rubin_cb1
rubin_RJF
ugc_586_1
ugc_586_10
ugc_586_11
ugc_586_12
ugc_586_18
ugc_586_19
ugc_586_2
ugc_586_3
ugc_586_4
ugc_586_5
ugc_586_6
ugc_586_7
ugc_586_8
ugc_586_9
ugc_586_13
ugc_586_14
ugc_586_15
ugc_586_22
ugc_586_23
ugc_586_25
ugc_586_17
ugc_586_20
ugc_586_21
ugc_610
East
North
West
RJF
127
113
98.8
84.7
70.6
56.5
42.4
28.2
14.1
1e−06

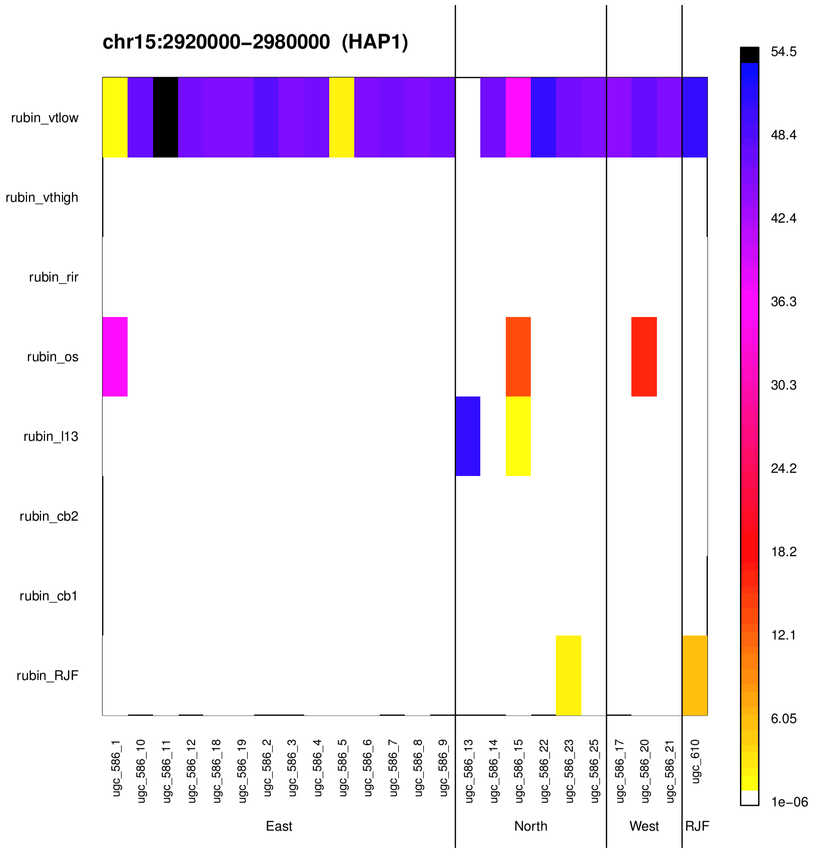
chr15:2920000–2980000 (HAP1)
rubin_vtlow
rubin_vthigh
rubin_rir
rubin_os
rubin_l13
rubin_cb2
rubin_cb1
rubin_RJF
ugc_586_1
ugc_586_10
ugc_586_11
ugc_586_12
ugc_586_18
ugc_586_19
ugc_586_2
ugc_586_3
ugc_586_4
ugc_586_5
ugc_586_6
ugc_586_7
ugc_586_8
ugc_586_9
ugc_586_13
ugc_586_14
ugc_586_15
ugc_586_22
ugc_586_23
ugc_586_25
ugc_586_17
ugc_586_20
ugc_586_21
ugc_610
East
North
West
RJF
54.5
48.4
42.4
36.3
30.3
24.2
18.2
12.1
6.05
1e−06

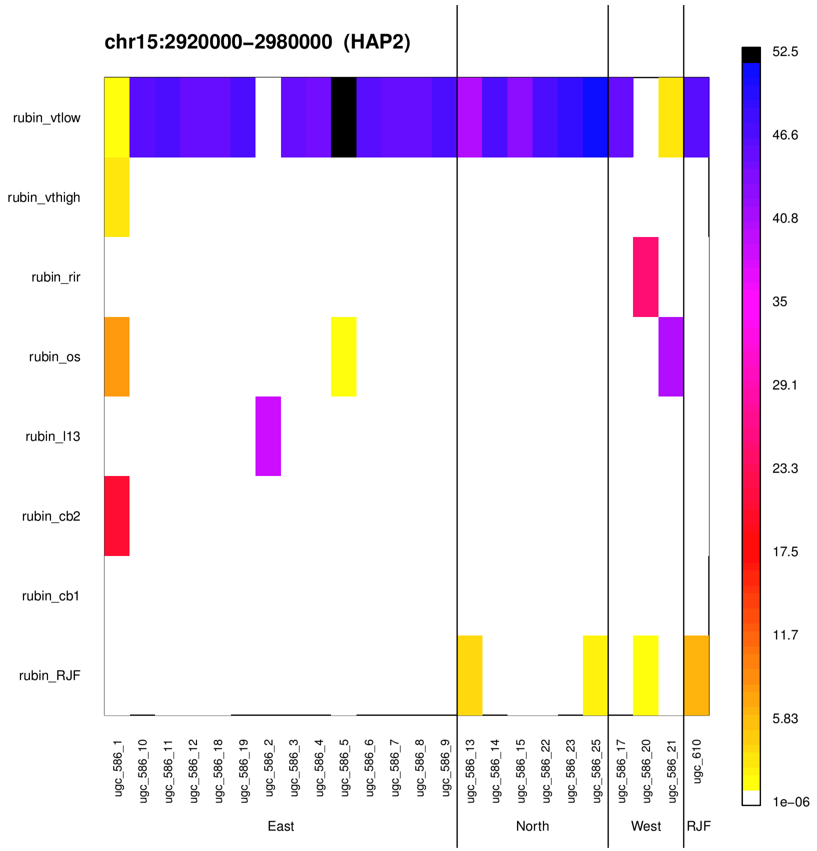
chr15:2920000–2980000 (HAP2)
rubin_vtlow
rubin_vthigh
rubin_rir
rubin_os
rubin_l13
rubin_cb2
rubin_cb1
rubin_RJF
ugc_586_1
ugc_586_10
ugc_586_11
ugc_586_12
ugc_586_18
ugc_586_19
ugc_586_2
ugc_586_3
ugc_586_4
ugc_586_5
ugc_586_6
ugc_586_7
ugc_586_8
ugc_586_9
ugc_586_13
ugc_586_14
ugc_586_15
ugc_586_22
ugc_586_23
ugc_586_25
ugc_586_17
ugc_586_20
ugc_586_21
ugc_610
East
North
West
RJF
52.5
46.6
40.8
35
29.1
23.3
17.5
11.7
5.83
1e−06

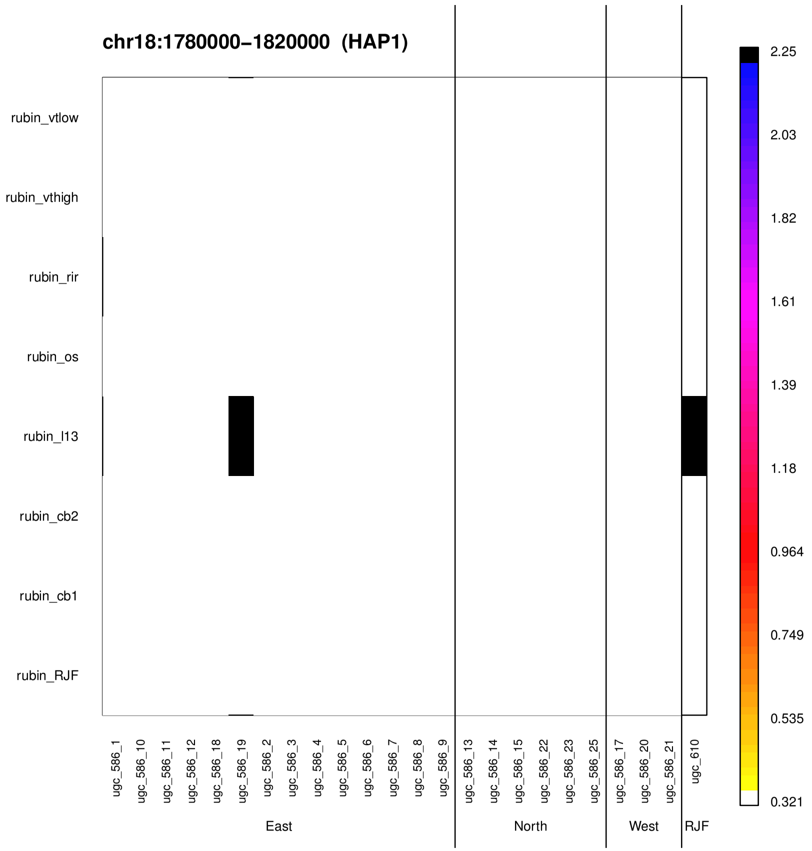
chr18:1780000−1820000 (HAP1)
rubin_vtlow
rubin_vthigh
rubin_rir
rubin_os
rubin_l13
rubin_cb2
rubin_cb1
rubin_RJF
ugc_586_1
ugc_586_10
ugc_586_11
ugc_586_12
ugc_586_18
ugc_586_19
ugc_586_2
ugc_586_3
ugc_586_4
ugc_586_5
ugc_586_6
ugc_586_7
ugc_586_8
ugc_586_9
ugc_586_13
ugc_586_14
ugc_586_15
ugc_586_22
ugc_586_23
ugc_586_25
ugc_586_17
ugc_586_20
ugc_586_21
ugc_610
East
North
West
RJF
2.25
2.03
1.82
1.61
1.39
1.18
0.964
0.749
0.535
0.321

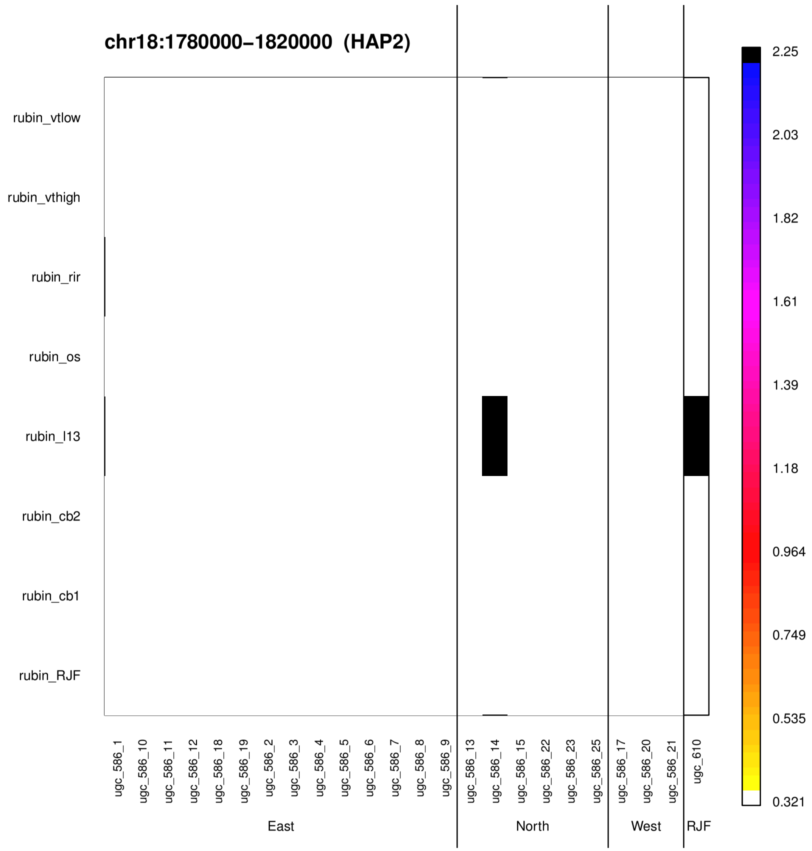
chr18:1780000−1820000 (HAP2)
rubin_vtlow
rubin_vthigh
rubin_rir
rubin_os
rubin_l13
rubin_cb2
rubin_cb1
rubin_RJF
ugc_586_1
ugc_586_10
ugc_586_11
ugc_586_12
ugc_586_18
ugc_586_19
ugc_586_2
ugc_586_3
ugc_586_4
ugc_586_5
ugc_586_6
ugc_586_7
ugc_586_8
ugc_586_9
ugc_586_13
ugc_586_14
ugc_586_15
ugc_586_22
ugc_586_23
ugc_586_25
ugc_586_17
ugc_586_20
ugc_586_21
ugc_610
East
North
West
RJF
2.25
2.03
1.82
1.61
1.39
1.18
0.964
0.749
0.535
0.321

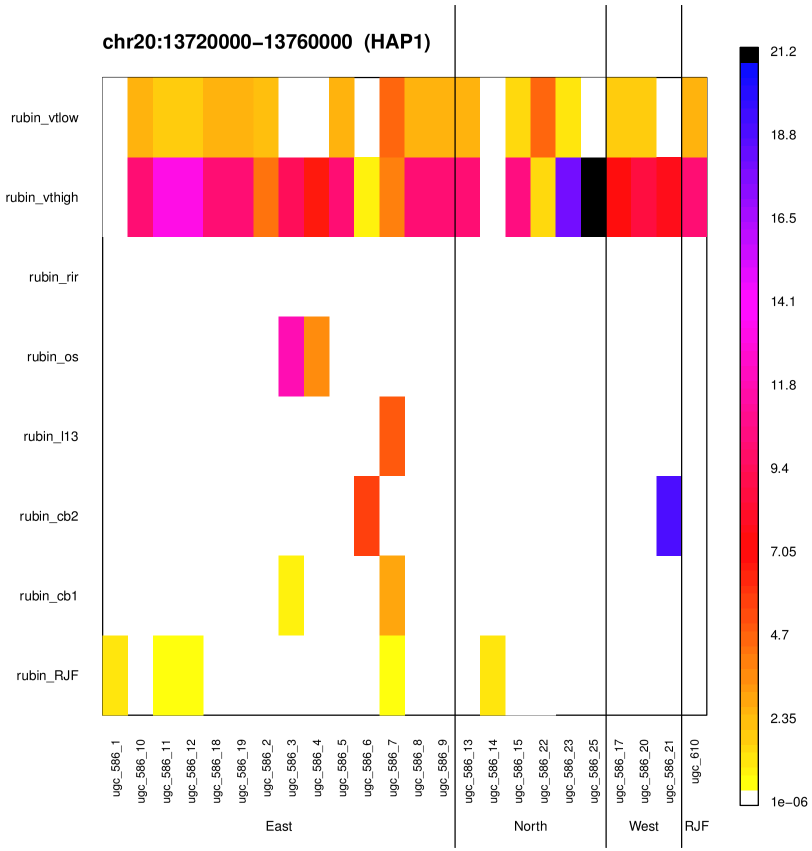
chr20:13720000–13760000 (HAP1)
rubin_vtlow
rubin_vthigh
rubin_rir
rubin_os
rubin_l13
rubin_cb2
rubin_cb1
rubin_RJF
ugc_586_1
ugc_586_10
ugc_586_11
ugc_586_12
ugc_586_18
ugc_586_19
ugc_586_2
ugc_586_3
ugc_586_4
ugc_586_5
ugc_586_6
ugc_586_7
ugc_586_8
ugc_586_9
ugc_586_13
ugc_586_14
ugc_586_15
ugc_586_22
ugc_586_23
ugc_586_25
ugc_586_17
ugc_586_20
ugc_586_21
ugc_610
East
North
West
RJF
21.2
18.8
16.5
14.1
11.8
9.4
7.05
4.7
2.35
1e−06

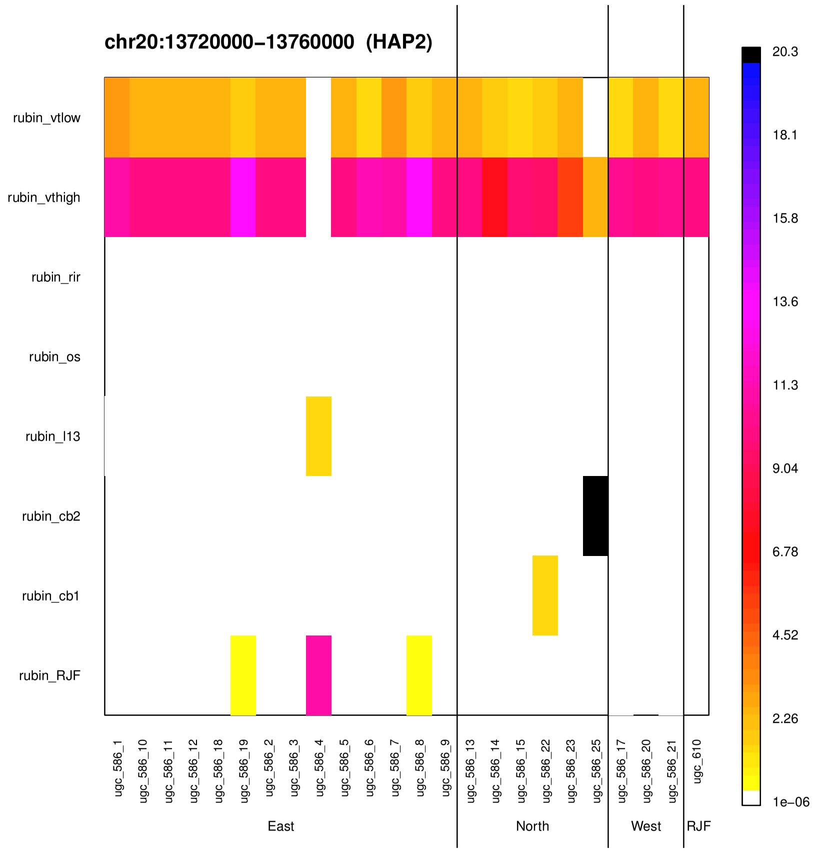
chr20:13720000–13760000 (HAP2)
rubin_vtlow
rubin_vthigh
rubin_rir
rubin_os
rubin_l13
rubin_cb2
rubin_cb1
rubin_RJF
ugc_586_1
ugc_586_10
ugc_586_11
ugc_586_12
ugc_586_18
ugc_586_19
ugc_586_2
ugc_586_3
ugc_586_4
ugc_586_5
ugc_586_6
ugc_586_7
ugc_586_8
ugc_586_9
ugc_586_13
ugc_586_14
ugc_586_15
ugc_586_22
ugc_586_23
ugc_586_25
ugc_586_17
ugc_586_20
ugc_586_21
ugc_610
East
North
West
RJF
20.3
18.1
15.8
13.6
11.3
9.04
6.78
4.52
2.26
1e−06

chr20:14140000–14200000 (HAP2)

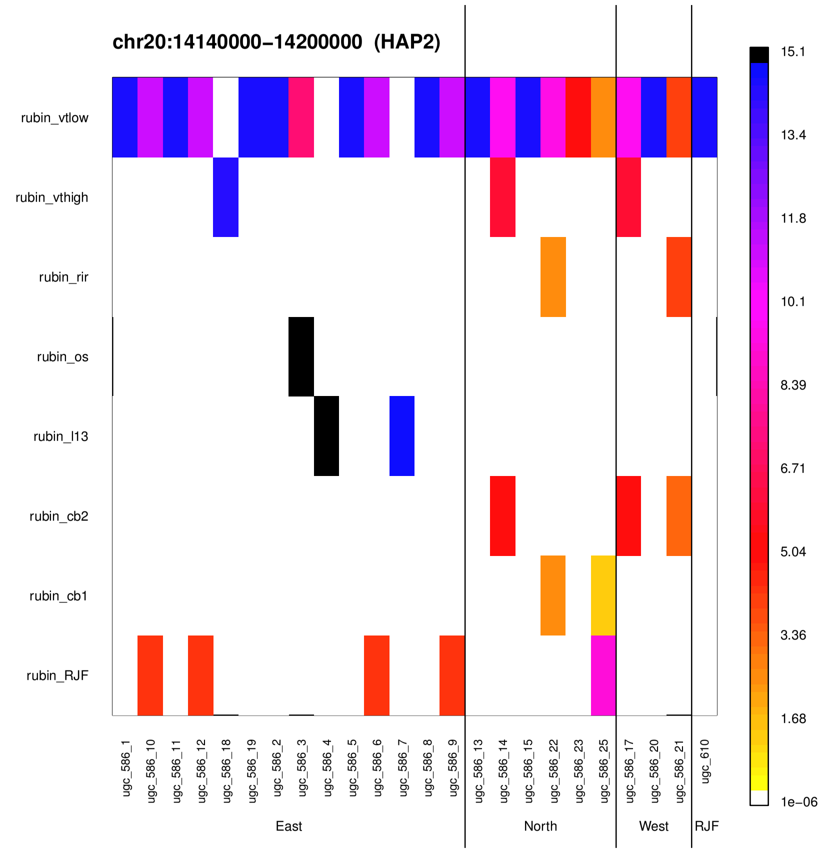

chr20:14140000–14200000 (HAP1)

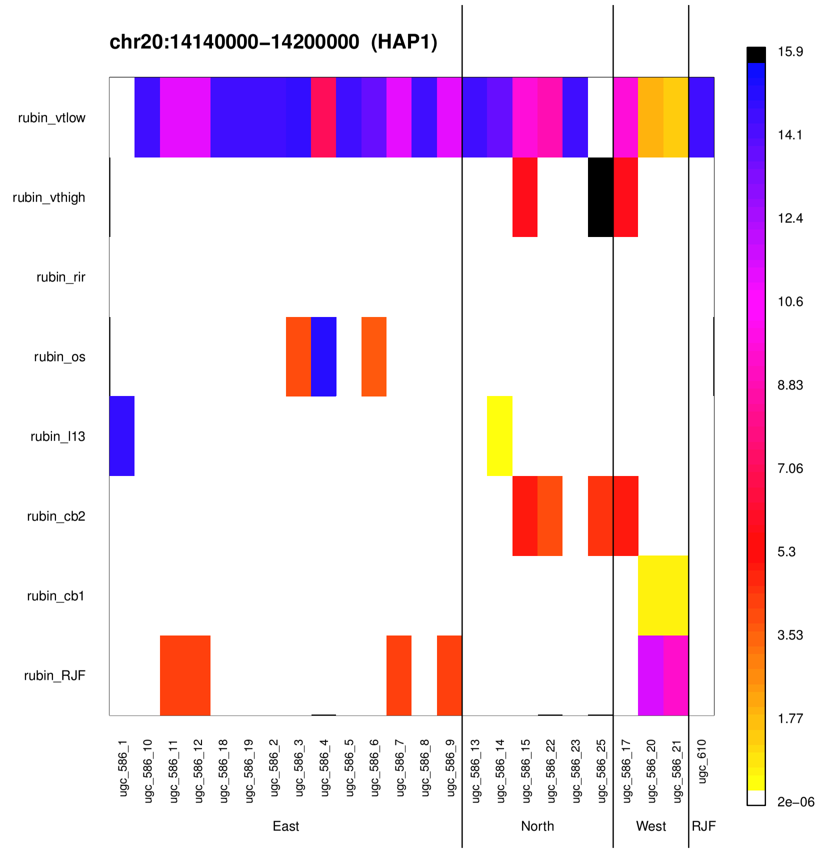

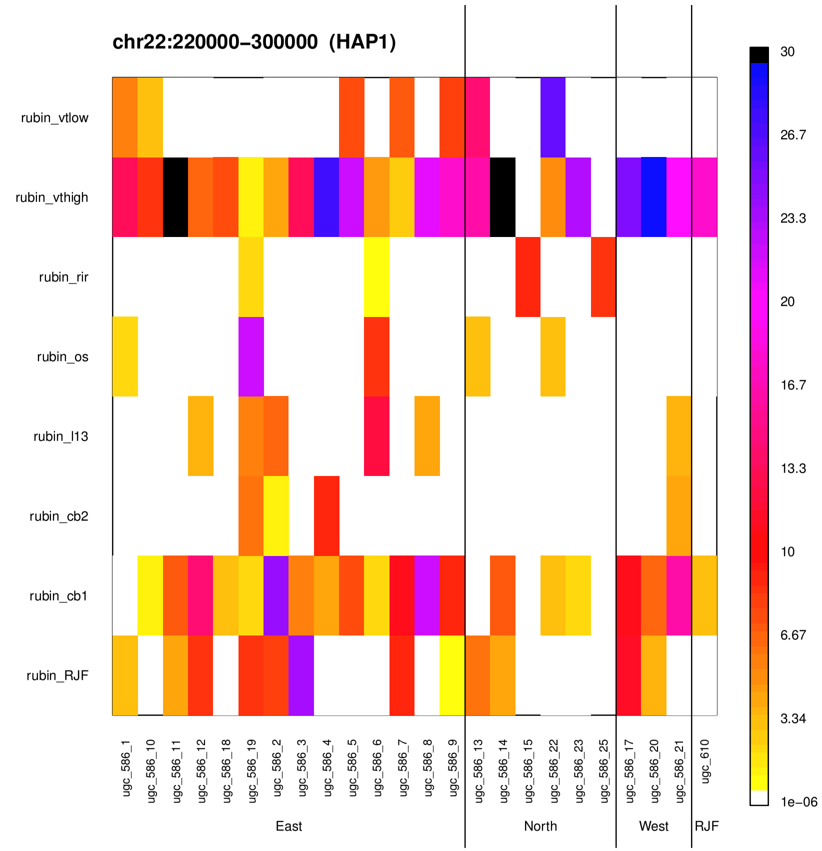
chr22:220000-300000 (HAP1)
rubin_vtlow
rubin_vthigh
rubin_rir
rubin_os
rubin_l13
rubin_cb2
rubin_cb1
rubin_RJF
ugc_586_1
ugc_586_10
ugc_586_11
ugc_586_12
ugc_586_18
ugc_586_19
ugc_586_2
ugc_586_3
ugc_586_4
ugc_586_5
ugc_586_6
ugc_586_7
ugc_586_8
ugc_586_9
ugc_586_13
ugc_586_14
ugc_586_15
ugc_586_22
ugc_586_23
ugc_586_25
ugc_586_17
ugc_586_20
ugc_586_21
ugc_610
East
North
West
RJF
30
26.7
23.3
20
16.7
13.3
10
6.67
3.34
1e-06

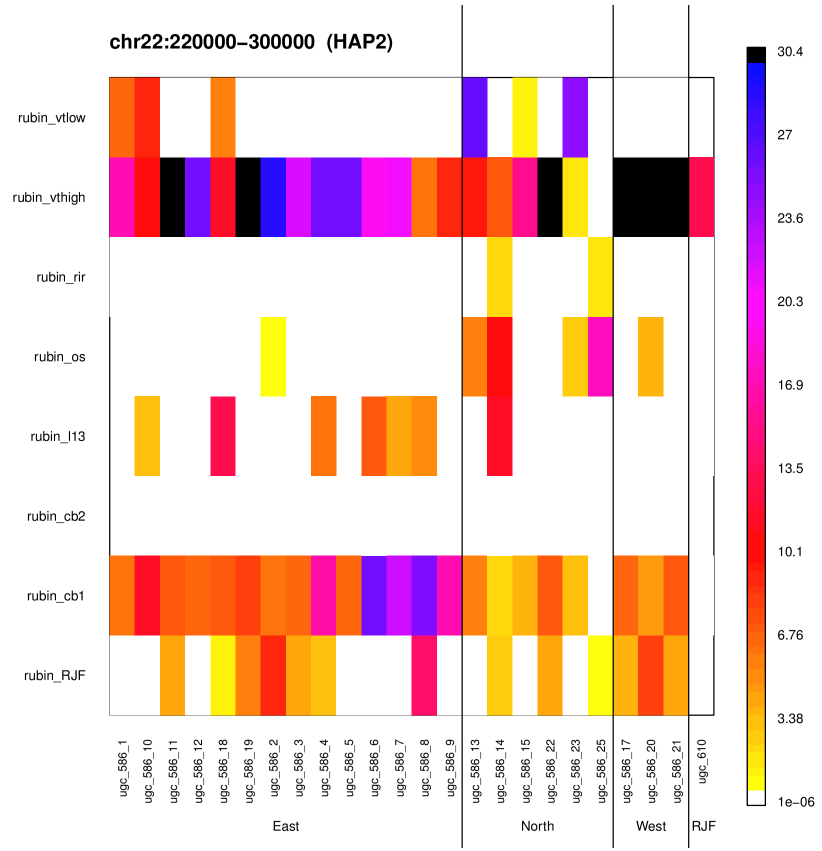
chr22:220000-300000 (HAP2)
rubin_vtlow
rubin_vthigh
rubin_rir
rubin_os
rubin_l13
rubin_cb2
rubin_cb1
rubin_RJF
ugc_586_1
ugc_586_10
ugc_586_11
ugc_586_12
ugc_586_18
ugc_586_19
ugc_586_2
ugc_586_3
ugc_586_4
ugc_586_5
ugc_586_6
ugc_586_7
ugc_586_8
ugc_586_9
ugc_586_13
ugc_586_14
ugc_586_15
ugc_586_22
ugc_586_23
ugc_586_25
ugc_586_17
ugc_586_20
ugc_586_21
ugc_610
East
North
West
RJF
30.4
27
23.6
20.3
16.9
13.5
10.1
6.76
3.38
1e-06

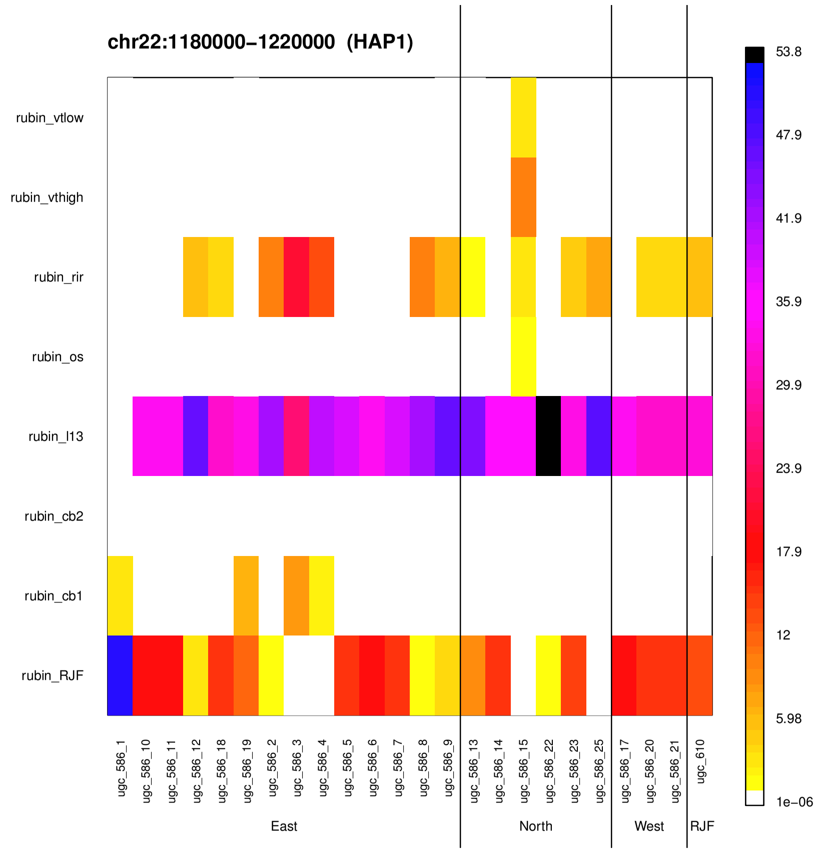
chr22:1180000–1220000 (HAP1)
rubin_vtlow
rubin_vthigh
rubin_rir
rubin_os
rubin_l13
rubin_cb2
rubin_cb1
rubin_RJF
ugc_586_1
ugc_586_10
ugc_586_11
ugc_586_12
ugc_586_18
ugc_586_19
ugc_586_2
ugc_586_3
ugc_586_4
ugc_586_5
ugc_586_6
ugc_586_7
ugc_586_8
ugc_586_9
ugc_586_13
ugc_586_14
ugc_586_15
ugc_586_22
ugc_586_23
ugc_586_25
ugc_586_17
ugc_586_20
ugc_586_21
ugc_610
East
North
West
RJF
53.8
47.9
41.9
35.9
29.9
23.9
17.9
12
5.98
1e–06

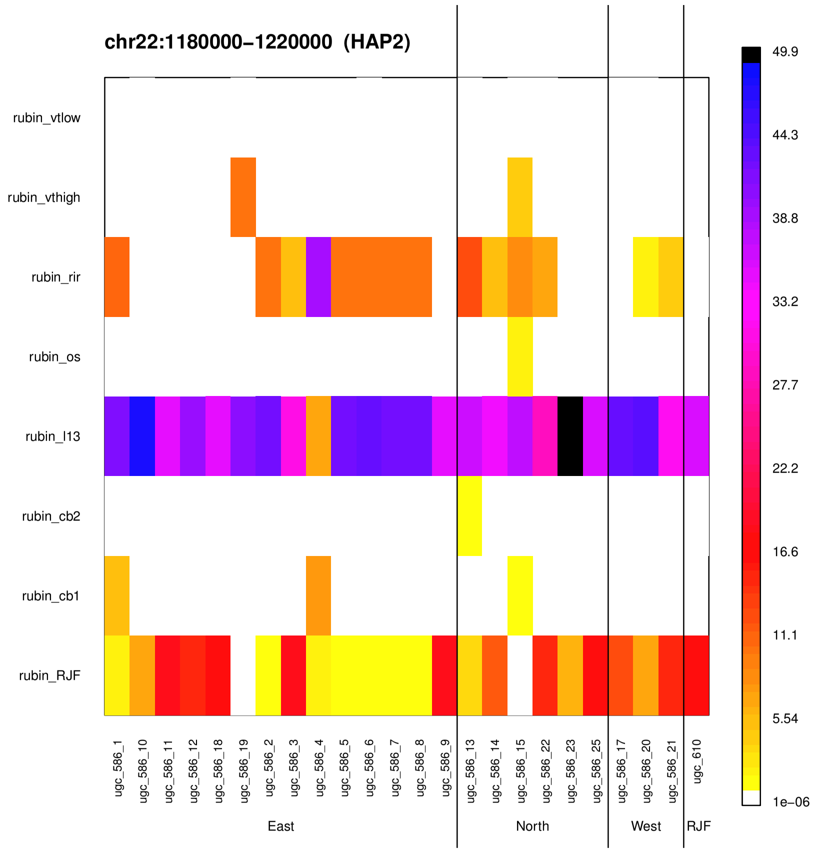
chr22:1180000–1220000 (HAP2)
rubin_vtlow
rubin_vthigh
rubin_rir
rubin_os
rubin_l13
rubin_cb2
rubin_cb1
rubin_RJF
ugc_586_1
ugc_586_10
ugc_586_11
ugc_586_12
ugc_586_18
ugc_586_19
ugc_586_2
ugc_586_3
ugc_586_4
ugc_586_5
ugc_586_6
ugc_586_7
ugc_586_8
ugc_586_9
ugc_586_13
ugc_586_14
ugc_586_15
ugc_586_22
ugc_586_23
ugc_586_25
ugc_586_17
ugc_586_20
ugc_586_21
ugc_610
East
North
West
RJF
49.9
44.3
38.8
33.2
27.7
22.2
16.6
11.1
5.54
1e–06

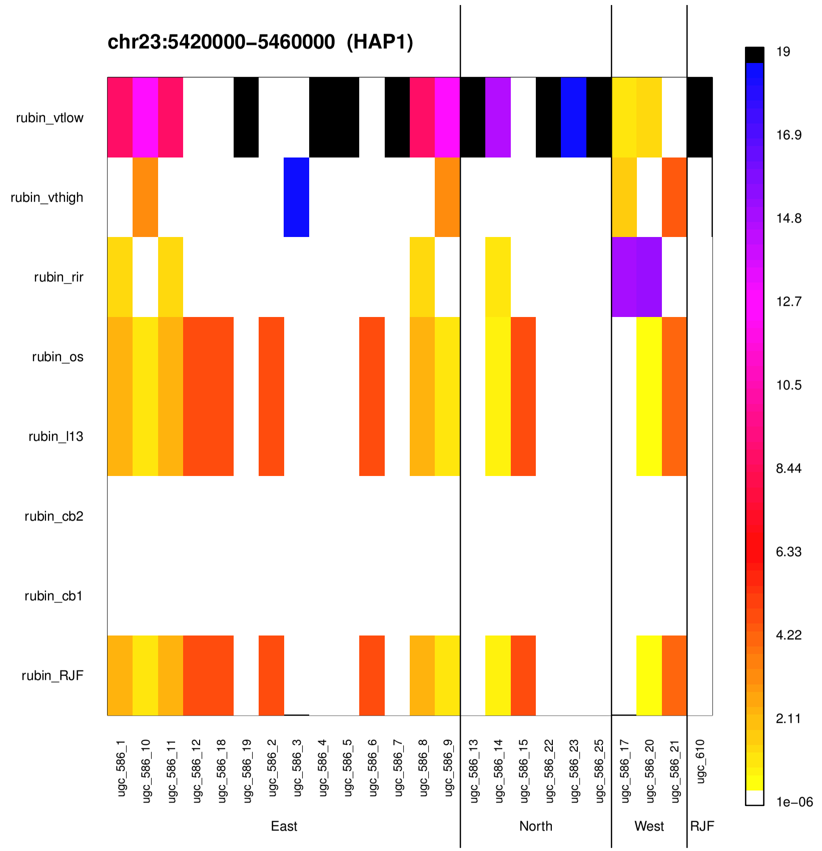
chr23:5420000–5460000 (HAP1)
rubin_vtlow
rubin_vthigh
rubin_rir
rubin_os
rubin_l13
rubin_cb2
rubin_cb1
rubin_RJF
ugc_586_1
ugc_586_10
ugc_586_11
ugc_586_12
ugc_586_18
ugc_586_19
ugc_586_2
ugc_586_3
ugc_586_4
ugc_586_5
ugc_586_6
ugc_586_7
ugc_586_8
ugc_586_9
ugc_586_13
ugc_586_14
ugc_586_15
ugc_586_22
ugc_586_23
ugc_586_25
ugc_586_17
ugc_586_20
ugc_586_21
ugc_610
East
North
West
RJF
19
16.9
14.8
12.7
10.5
8.44
6.33
4.22
2.11
1e–06

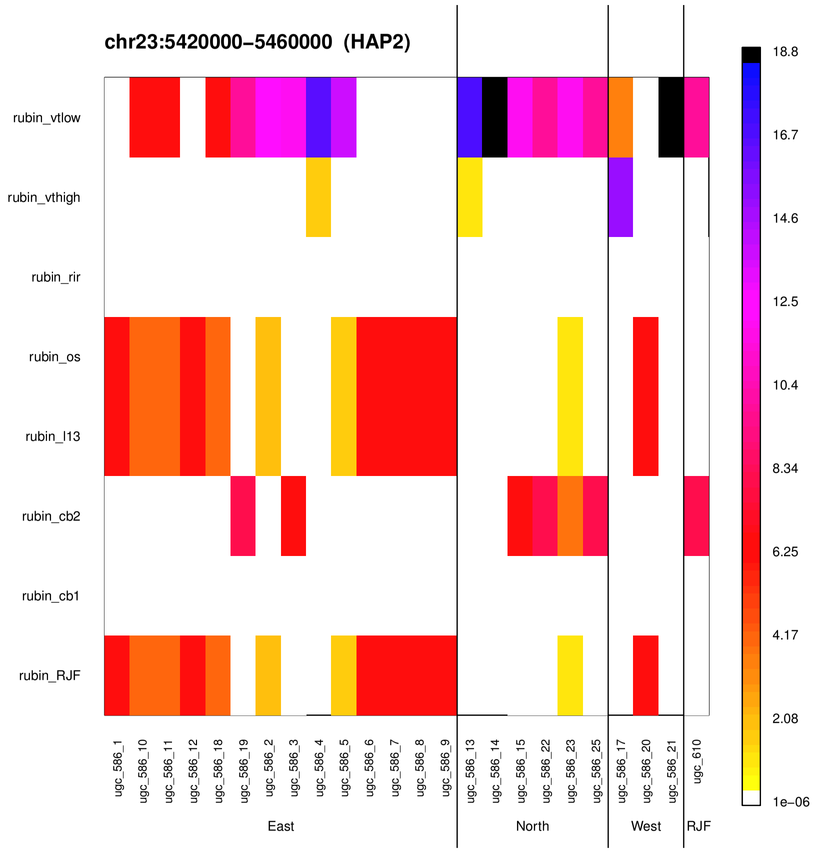
chr23:5420000–5460000 (HAP2)
rubin_vtlow
rubin_vthigh
rubin_rir
rubin_os
rubin_l13
rubin_cb2
rubin_cb1
rubin_RJF
ugc_586_1
ugc_586_10
ugc_586_11
ugc_586_12
ugc_586_18
ugc_586_19
ugc_586_2
ugc_586_3
ugc_586_4
ugc_586_5
ugc_586_6
ugc_586_7
ugc_586_8
ugc_586_9
ugc_586_13
ugc_586_14
ugc_586_15
ugc_586_22
ugc_586_23
ugc_586_25
ugc_586_17
ugc_586_20
ugc_586_21
ugc_610
East
North
West
RJF
18.8
16.7
14.6
12.5
10.4
8.34
6.25
4.17
2.08
1e–06

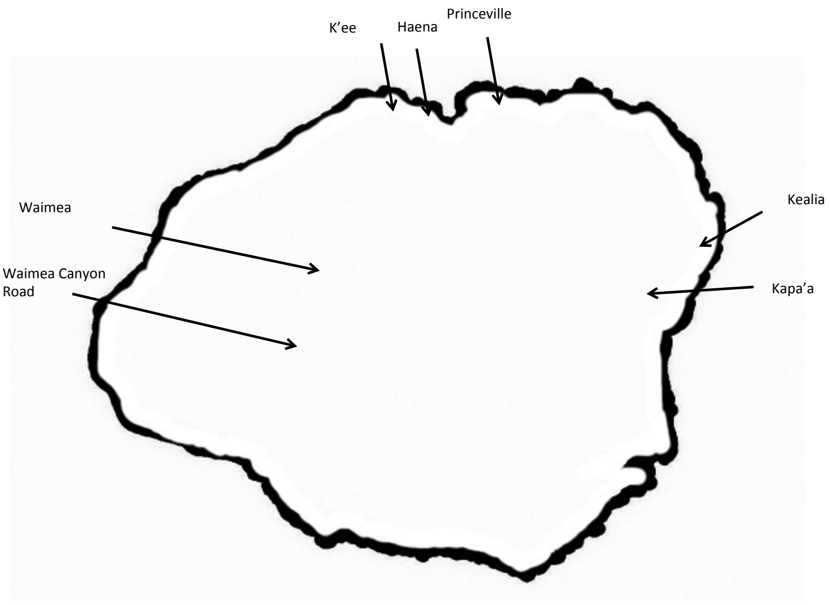

Supplementary Figure 3. Map showing locations of sample collection.

sample	id	mapped_reads	read_length (bp)	coverage (X)	location
KAP001	ugc_586_1	41391302	75	3.0	Kapa'a
KAP002	ugc_586_2	51671738	75	3.7	Kapa'a
KAP003	ugc_586_3	48602159	75	3.5	Kapa'a
KAP004	ugc_586_4	58513864	75	4.2	Kapa'a
KAP006	ugc_586_5	50013629	75	3.6	Kapa'a
KAP007	ugc_586_6	52935023	75	3.8	Kapa'a
KAP008	ugc_586_7	60669889	75	4.3	Kapa'a
KAP009	ugc_586_8	82543042	75	5.9	Kapa'a
KAP011	ugc_586_9	51622278	75	3.7	Kapa'a
KAP012	ugc_586_10	73735939	75	5.3	Kapa'a
KAP013	ugc_586_11	48661720	75	3.5	Kapa'a
KAP014	ugc_586_12	85453975	75	6.1	Kapa'a
RK001	ugc_586_13	47104494	75	3.4	Haena
RK002	ugc_586_14	75716532	75	5.4	Princeville_West
RK003	ugc_586_15	94987243	75	6.8	Kilauea
WCR001	ugc_586_17	42016743	75	3.0	Waimea_Canyon_Road
TON001	ugc_586_18	85916590	75	6.1	Kealia
TON003	ugc_586_19	68830773	75	4.9	Kealia
WAI001	ugc_586_20	88586975	75	6.3	Waimea
WAI002	ugc_586_21	91862350	75	6.6	Waimea
PV001	ugc_586_22	54632484	75	3.9	Princeville
PV002	ugc_586_23	93214209	75	6.7	Princeville
HAE001	ugc_586_25	89872084	75	6.4	K'ee
Götala RJF	ugc_610	55532954	75	4.0	Thailand
SRR035383+SRR035384	rubin_rjf	188196258	35	6.3	NA
SRR035375+SRR035389 +SRR035390	rubin_l13	126644229	35	4.2	NA
SRR035385	rubin_os	96525559	35	3.2	NA
SRR035376	rubin_wlb	106335483	35	3.5	NA
SRR035388	rubin_rir	82260768	35	2.7	NA
SRR035381+SRR035382	rubin_vtlow	153622683	35	5.1	NA
SRR035379+SRR035380	rubin_vthigh	144020959	35	4.8	NA
SRR035387	rubin_cb2	103701990	35	3.5	NA
SRR035377+SRR035378	rubin_cb1	146554680	35	4.9	NA

Supplementary Table 1. Sample and sequencing information.

Chromosome	Start	End	Kauai ZHp	LR ZHp	CB ZHp	AD ZHp
chr1	8480000	8520000	-5,0	-0,7	-0,3	-4,0
chr1	8520000	8560000	-4,3	-0,9	-4,1	-5,1
chr1	8540000	8580000	-5,3	-0,9	-5,2	-6,0
chr1	8560000	8600000	-4,3	-1,3	-5,5	-6,1
chr1	8580000	8620000	-4,2	-1,8	-4,7	-4,9
chr1	147700000	147740000	-4,2	-4,5	-4,5	-6,4
chr1	159880000	159920000	-4,8	-3,1	0,3	-2,0
chr1	190580000	190620000	-4,0	0,3	0,5	3,0
chr1	190600000	190640000	-4,3	-0,3	1,0	1,9
chr1	190620000	190660000	-4,4	-0,7	1,0	1,7
chr1	190640000	190680000	-4,1	-0,6	0,6	2,0
chr13	17480000	17520000	-4,3	-0,3	-1,1	-1,6
chr13	17500000	17540000	-5,0	-0,6	-0,5	-2,1
chr13	17520000	17560000	-5,7	-0,9	-0,9	-2,7
chr13	17540000	17580000	-4,4	-0,5	-0,7	-2,5
chr13	17580000	17620000	-4,8	0,3	-0,6	-1,7
chr13	17600000	17640000	-5,3	0,2	-2,4	-2,7
chr13	17620000	17660000	-4,6	0,1	-1,7	-2,5
chr13	17640000	17680000	-4,2	1,0	-1,0	-1,6
chr15	2920000	2960000	-4,0	-1,9	1,6	2,1
chr15	2940000	2980000	-4,1	-3,1	-0,3	-0,1
chr18	1780000	1820000	-4,6	2,1	0,0	-0,5
chr2	280000	320000	-4,0	-0,2	-1,0	-1,0
chr2	72860000	72900000	-4,1	-2,8	-1,7	-0,4
chr2	73260000	73300000	-4,7	-2,8	2,5	-1,2
chr2	73560000	73600000	-4,2	-0,8	1,9	0,3
chr2	140960000	141000000	-4,2	0,1	-2,9	-2,7
chr2	140980000	141020000	-4,1	0,0	-3,5	-3,5
chr2	141000000	141040000	-4,0	0,5	-2,7	-3,1
chr2	143000000	143040000	-5,1	-2,9	1,5	-1,2
chr2	143020000	143060000	-5,5	-3,2	1,7	-2,5
chr2	143040000	143080000	-5,4	-3,4	2,5	-2,6
chr2	143060000	143100000	-4,3	-3,7	3,6	-1,3
chr2	143660000	143700000	-4,7	0,7	1,5	1,0
chr2	146660000	146700000	-4,3	-2,0	0,2	-4,0
chr20	13720000	13760000	-4,3	-0,9	1,1	-1,2
chr20	14140000	14180000	-4,8	-2,4	-0,6	-3,0
chr20	14160000	14200000	-5,1	-2,0	-0,4	-3,8
chr22	220000	260000	-4,1	-1,6	0,9	-1,4
chr22	240000	280000	-6,1	-4,2	0,0	-4,8
chr22	260000	300000	-5,2	-4,4	-0,5	-4,8
chr22	1180000	1220000	-4,2	-0,8	-1,7	1,1
chr23	5420000	5460000	-4,5	-5,2	0,9	-2,2
chr3	1760000	1800000	-4,4	0,1	0,6	-0,4

chr3	1780000	1820000	-4,5	0,0	0,3	0,9
chr3	1800000	1840000	-4,4	-0,1	0,5	1,0
chr3	21180000	21220000	-4,7	-0,9	0,7	-3,1
chr3	21200000	21240000	-5,0	-2,0	-1,1	-2,9
chr3	60960000	61000000	-4,4	-3,6	-0,3	-3,3
chr3	83200000	83240000	-4,1	1,0	0,3	0,2
chr3	83220000	83260000	-4,2	0,3	-1,1	-0,1
chr3	83300000	83340000	-4,6	-1,0	-4,3	-3,6
chr3	83320000	83360000	-5,3	-1,3	-5,1	-4,2
chr3	83340000	83380000	-5,2	-1,2	-4,0	-3,3
chr3	83420000	83460000	-4,7	0,6	-3,9	-0,4
chr3	83440000	83480000	-4,7	0,9	-4,4	-1,4
chr4	9140000	9180000	-4,3	-1,6	-1,0	-1,5
chr4	77360000	77400000	-4,3	0,9	0,9	-1,3
chr4	81100000	81140000	-4,2	-5,0	-2,6	-4,2
chr5	3840000	3880000	-4,3	1,6	0,4	-2,3
chr5	20780000	20820000	-4,4	-0,2	2,2	0,7
chr5	20800000	20840000	-4,5	0,6	1,7	-1,4
chr5	20820000	20860000	-4,1	1,2	-0,8	-2,5
chr5	21680000	21720000	-4,9	-2,3	1,3	-2,0
chr5	21700000	21740000	-4,8	-1,7	1,9	-1,9
chr5	21720000	21760000	-5,4	-2,5	1,9	-3,1
chr5	21740000	21780000	-4,6	-3,2	2,4	-2,6
chr5	46400000	46440000	-4,1	1,1	2,3	2,5
chr6	13300000	13340000	-4,3	-3,0	1,8	-1,0
chr6	13320000	13360000	-4,0	-1,9	0,4	-1,7
chr6	16140000	16180000	-4,4	-0,2	0,3	-0,9
chr7	6240000	6280000	-4,1	1,8	0,5	-0,2
chr8	200000	240000	-4,2	0,4	2,3	1,0
chr8	220000	260000	-4,8	-1,9	0,4	-2,1
chr8	240000	280000	-4,4	-3,1	-0,8	-3,8
chr8	260000	300000	-4,5	-1,6	0,1	-2,1

Supplementary Table 2. Table comparing heterozygosity scores in the sweep regions detected in the Kauai population with the same regions in layer, broiler and domestic chicken pools.

Term	GO ID	Count
protein binding	GO:0005515	22
cytoplasm	GO:0005737	13
nucleus	GO:0005634	13
membrane	GO:0016020	10
plasma membrane	GO:0005886	9
integral component of membrane	GO:0016021	8
DNA binding	GO:0003677	7
metal ion binding	GO:0046872	7
extracellular exosome	GO:0070062	6
potassium ion transmembrane transport	GO:0071805	5

Supplementary Table 3. Table of common Gene Ontology terms in Kauai sweep genes.
